# *Seazzadactylus venieri* gen. et sp. nov., a new pterosaur (Diapsida: Pterosauria) from the Upper Triassic (Norian) of northeastern Italy

**DOI:** 10.7717/peerj.7363

**Published:** 2019-07-25

**Authors:** Fabio Marco Dalla Vecchia

**Affiliations:** 1 Research Group of Mesozoic Faunas, Institut Català de Paleontologia Miquel Crusafont (ICP), Sabadell, Catalonia, Spain; 2 Museo Friulano di Storia Naturale, Udine, Italy

**Keywords:** Vertebrate palaeontology, Pterosauria, New taxon, Anatomy, Taxonomy, Phylogeny, Diversity, Triassic

## Abstract

A new non-monofenestratan pterosaur with multicusped dentition, *Seazzadactylus venieri*, is described from the Upper Triassic (middle-upper Norian) of the Carnian Prealps (northeastern Italy). The holotype of *S. venieri* preserves a complete mandibular and maxillary dentition, along with a nearly complete premaxillary one, showing unique features. Furthermore, the arrangement of the premaxillary teeth and the shape of jugal, pterygoid, ectopterygoid, scapula and pteroid are unique within non-monofenestratan pterosaurs. *S. venieri* is similar and closely related to *Carniadactylus rosenfeldi* and *Austriadraco dallavecchiai*, which are also from the Alpine middle-upper Norian of Italy and Austria, respectively. In a parsimony-based phylogenetic analysis, *S. venieri* is found to nest within a clade of Triassic pterosaurs composed of *Arcticodactylus cromptonellus*, *Austriadraco dallavecchiai, Carniadactylus rosenfeldi* and a trichotomy of *Raeticodactylus filisurensis*, *Caviramus schesaplanensis* and MCSNB 8950. This unnamed clade is basal within the Pterosauria, but is not the basalmost clade. *Eudimorphodon ranzii* lies outside this clade and is more derived, making the Eudimorphodontidae paraphyletic. *S. venieri* increases the diversity of Triassic pterosaurs and brings the number of pterosaur genera and species in the Dolomia di Forni Formation to four.

## Introduction

Late Triassic (Norian) pterosaurs are the oldest ones found to date (Dalla Vecchia, 2013). They are represented by about 30 unequivocal remains, including fragmentary specimens and single isolated bones and teeth (Dalla Vecchia, 2013, 2014). Their record is rather sparse and each new find has therefore an impact upon our understanding of early pterosaur history and phylogenetic relationships.

*Eudimorphodon ranzii* from the Upper Triassic of Italy was the first valid Triassic pterosaur species to be named (Zambelli, 1973). It appeared to be characterised by tri- to pentacuspid maxillary and mandibular teeth. A relatively high number of skeletal remains from Italy, Austria, Greenland and USA, as well as many isolated teeth from Europe and North America, have subsequently been referred to this genus, based mainly on the presence of two to four accessory cusps in the tooth crowns (Dalla Vecchia, 2013, 2014). These specimens were initially referred to *E. ranzii* (MPUM 6009; Wild, 1979; MCSNB 8950; Wild, 1994; and BSP 1994 I 51; Wellnhofer, 2003), to a new *Eudimorphodon* species (MFSN 1797, holotype of *E. rosenfeldi* (see Dalla Vecchia, 1995) and MGUH VP 3393, holotype of *E. cromptonellus* (see Jenkins et al., 2001)), or just to the genus *Eudimorphodon* (Clemens, 1980; Hahn, Lepage & Wouters, 1984; Chatterjee, 1986; Murry, 1986; Cuny, 1995; Cuny, Godefroit & Martin, 1995; Godefroit, 1997; Godefroit & Cuny, 1997; Godefroit et al., 1998, Dalla Vecchia, 2003, 2004a, 2004b, 2006; Andres, 2006; Andres & Myers, 2013). However, most of the isolated teeth are probably referable to cynodont therapsids (Andres, 2006; Dalla Vecchia, 2013, 2014). Isolated multicusped teeth from Triassic rocks cannot be unequivocally referred to pterosaurs because of the convergent morphology of the teeth of some pterosaurs, cynodonts and also tanystropheid archosauromorphs. Furthermore, the discovery of *Caviramus schesaplanensis* (see Fröbisch & Fröbisch, 2006) and *Raeticodactylus filisurensis* (see Stecher, 2008) from the Upper Triassic of Switzerland showed that tri- to pentacuspid teeth occur also in other Triassic pterosaur taxa. As a consequence, multicusped teeth can no longer be considered a diagnostic feature of *Eudimorphodon*. Dalla Vecchia (2009a) referred *E. rosenfeldi* to a new genus *Carniadactylus* as *Carniadactylus rosenfeldi*. Dalla Vecchia (2009a, 2014) also suggested that the remains of *E. cromptonellus*, BSP 1994 I 51 and MCSNB 8950, belong to three distinct taxa that are different from *E. ranzii* (holotype, MCSNB 2888) because of the absence of shared apomorphies with the taxon and their morphological differences from it. Furthermore, MGUH VP 3393, BSP 1994 I 51, MCSNB 8950, MFSN 1797 and *E. ranzii* did not form a clade in the phylogenetic analyses of Dalla Vecchia (2009a, 2009b). *Eudimorphodon* as conceived by Wild (1979, 1994), Wellnhofer (2003), and Jenkins et al. (2001) is also paraphyletic within the phylogenetic analyses of Kellner (2003), Wang et al. (2009) and Ősi (2010). Kellner (2015) made BSP 1994 I 51 the holotype of *Austriadraco dallavecchiai*, and referred *E. cromptonellus* to the new genus *Arcticodactylus* as *Arcticodactylus cromptonellus*. Kellner (2015) made MPUM 6009 the holotype of *Bergamodactylus wildi* but Dalla Vecchia (2018) retained MPUM 6009 in *Carniadactylus rosenfeldi*.

**Figure 1:**
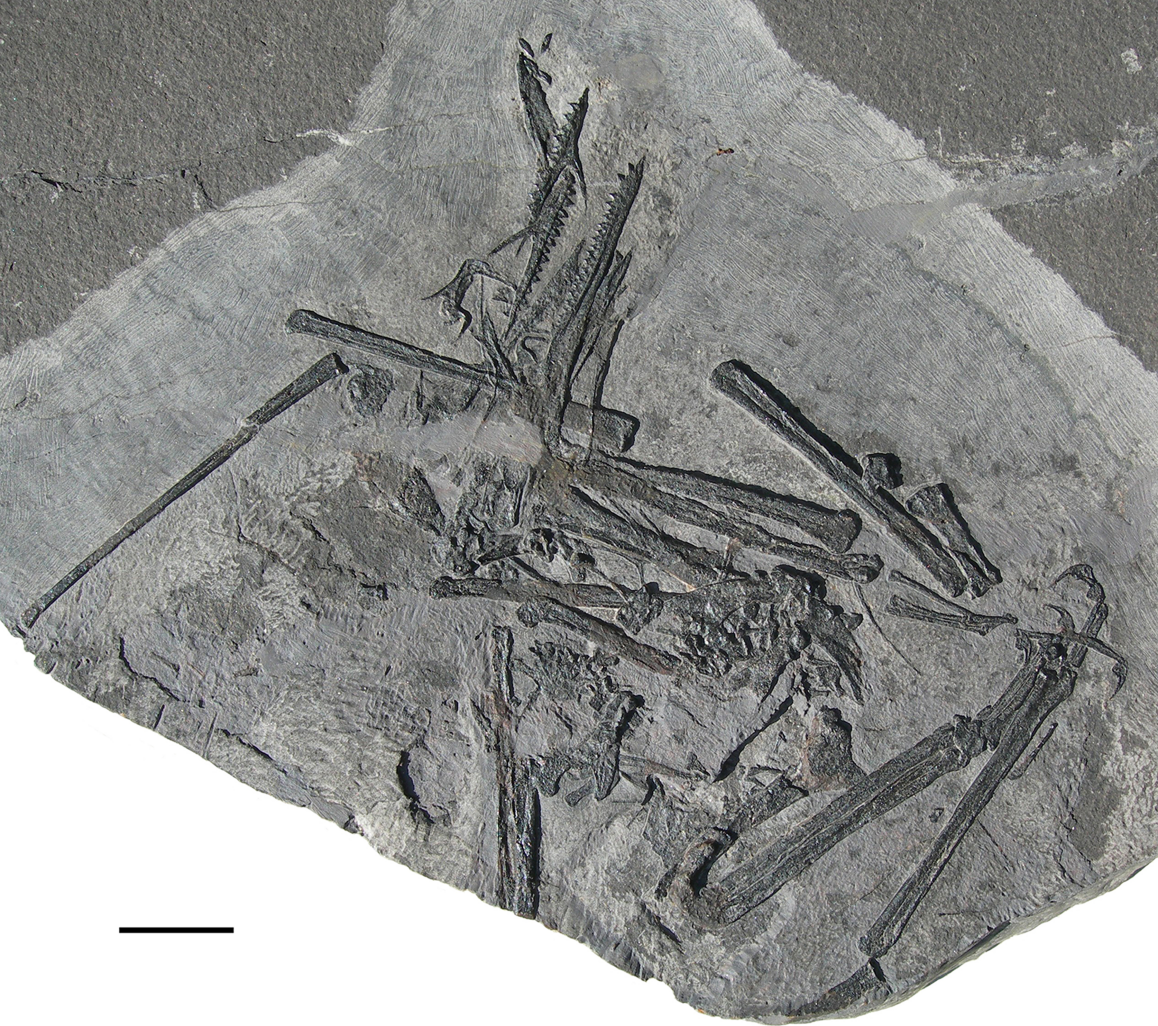
*Seazzadactylus venieri*, MFSN 21545 (holotype). Photograph. Scale bar equals 20 mm.

**Figure 2:**
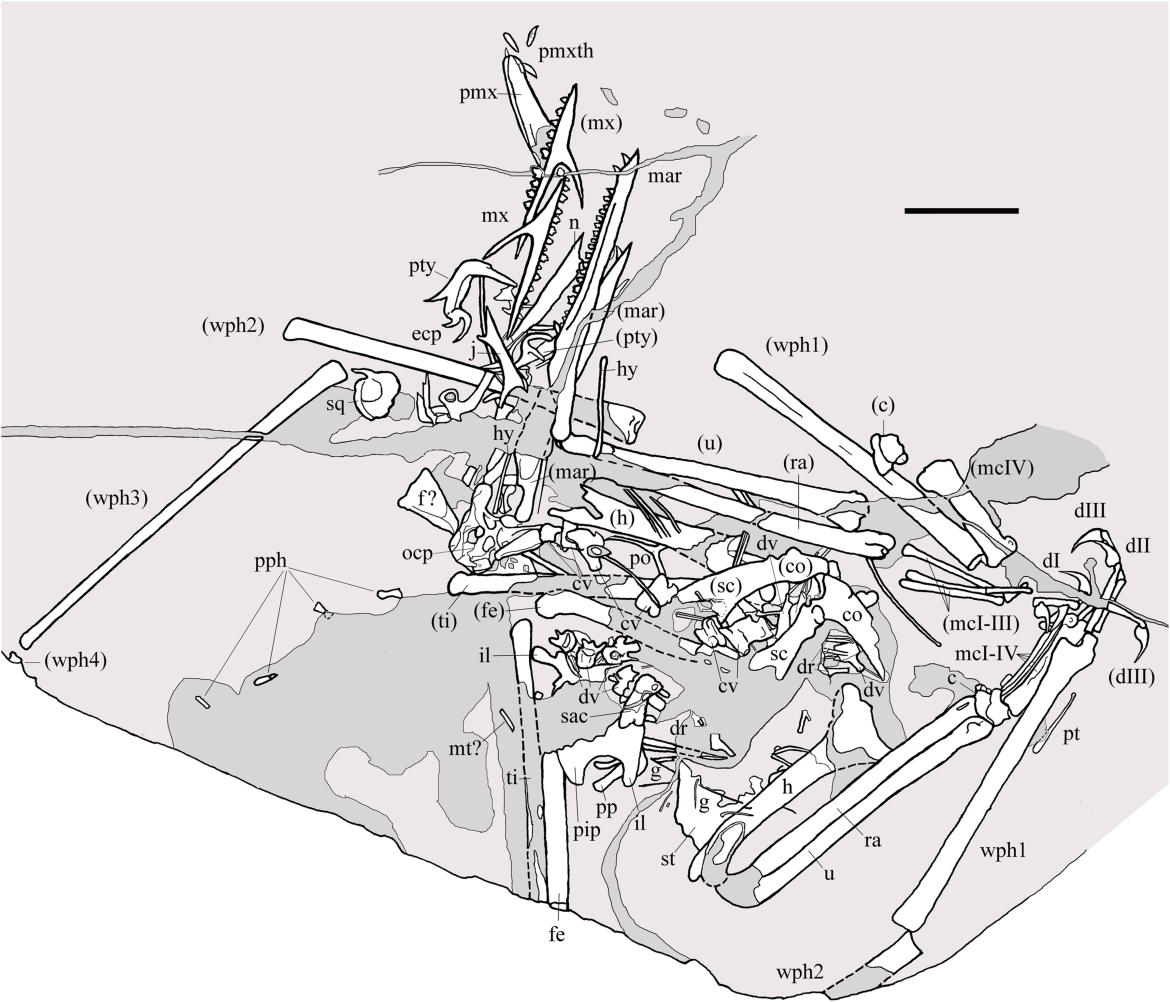
*Seazzadactylus venieri*, MFSN 21545 (holotype). Drawing. Abbreviations: c, carpus; co, coracoid; cv, cervical vertebra; dI–III, manus digits I–III; dr, dorsal rib; dv, dorsal vertebra; ecp, ectopterygoid; f, frontal; fe, femur; g, gastralia; h, humerus; hy, ceratobranchial I (hyoid apparatus); j, jugal; il, ilium; mar, mandibular ramus; mcI–IV, metacarpals I–IV; mt, metatarsal; mx, maxilla; n, nasal; ocp, occiput; pip, puboischiadic plate; pmx, premaxillae; pmxth, premaxillary teeth; po, postorbital; pp, prepubis; pph, pes phalanges; pt, pteroid; pty, pterygoid; ra, radius; sac, sacrum; sc, scapula; sq, squamosal; st, sternum; ti, tibiotarsus; u, ulna; wph1–4, wing phalanges 1–4. When it was possible to distinguish between right and left elements, elements in parentheses are from the left side. Scale bar equals 20 mm.

Another pterosaur specimen, MFSN 21545 (Figs. 1, 2; Fig. S1), was mentioned in literature (see the list of synonyms below), but it was never described in detail. Initially, it was provisionally referred to the genus *Eudimorphodon* because of its ‘eudimorphodontid’ dentition (Dalla Vecchia, 2003, 2004a, 2004b, 2006, 2008), but was later considered to represent a yet unnamed taxon distinct from *E. ranzii* and *Carniadactylus rosenfeldi* (see Dalla Vecchia, 2009a, 2010, 2012, 2013, 2014). It was not included in Dalla Vecchia’s (2009a, 2009b) phylogenetic analyses because at the time some skeletal elements of the specimen were still under preparation.

Here, MFSN 21545 is described in detail and named, and the phylogenetic position of this new taxon is investigated.

### Locality and geological setting

According to the discoverer, Mr. Umberto Venier, MFSN 21545 was preserved in a loose boulder in the bed of the Seazza Brook (Preone Municipality, Friuli Venezia Giulia Autonomous Region, NE Italy; Fig. S2) at ca. 435 m above the sea level, just upstream of the angle bend in the final tract of the brook before it issues into the Tagliamento River.

The boulder lithology (dark grey laminated dolostone) and the local stratigraphy (Dalla Vecchia, 2012), as well as geomorphologic and topographic constraints, indicate that the specimen comes from the lower member of the Dolomia di Forni Formation (sensu Dalla Vecchia, 1991; see also Dalla Vecchia, 2012), possibly from its lower portion. The fossiliferous portion of the Dolomia di Forni Formation was dated to the late middle to late Norian (Alaunian 3-Sevatian) on the basis of its conodont assemblages (Dalla Vecchia, 2014).

## Materials, Terminology and Methods

MFSN 21545 is the only known specimen of the new taxon here described. It is a disarticulated partial skeleton preserving skull elements, both mandibular rami with teeth, the ossified hyoid elements, part of the cervical, dorsal and sacral vertebral column, most of the pectoral girdle and forelimbs and part of the pelvic girdles with hind limbs (Figs. 1 and 2).

The term ‘non-monofenestratan pterosaur’ is used for all the genera once included in the Suborder Rhamphorhynchoidea of the traditional Linnean classification (see Wellnhofer, 1978), which is now a paraphyletic group according to multiple phylogenetic analyses (Kellner, 2003; Unwin, 2003; Dalla Vecchia, 2009a). Enclosure in single quotation marks in the following part of the text indicates that the validity of the taxon is doubtful or in need of a formal revision.

Following Dalla Vecchia (2009a), *E. ranzii* is considered to be represented by the only holotype (MCSNB 2888) and MPUM 6009 is retained in *Carniadactylus rosenfeldi* (according to Dalla Vecchia, 2018 and contra Kellner, 2015). *Raeticodactylus filisurensis* is probably congeneric with *Caviramus schesaplanensis* (see Dalla Vecchia, 2009a); however, I followed Dalla Vecchia (2014) in keeping distinct the two taxa, pending their formal revision hopefully based on further specimens. Specimen MCSNB 8950 (*E. ranzii* for Wild, 1994) does not belong to *E. ranzii* and represents a distinct, still unnamed taxon according to Dalla Vecchia (2009a, 2014); it is used here as a terminal taxon in the phylogenetic analysis. Specimen MCSNB 2887 (*E. ranzii* for Wild, 1979) is considered to belong to an indeterminate pterosaur taxon following Dalla Vecchia (2014); it was used in the taxonomic comparison but not in the phylogenetic analysis.

The orientation of the forelimb bones is in the flight position and the terminology used by Bennett (2001) was followed for the orientation of the bones in the space, but ‘cranial’ and ‘caudal’ are preferred to ‘anterior’ and ‘posterior’. The anatomical terminology for the skeleton is that of Romer (1956), unless specified otherwise. The terminology used for teeth and dentition is in general that suggested by Edmund (1969). The term ‘cusps’ indicates topographically separate elevations along the cutting margins of a tooth crown that are few in number. A tooth is considered serrated when those elevations (denticles) are small, of similar sizes, and set close to one other in a row along most of the cutting margins of the crown. Crenulations are low, blunt, well-spaced and barely distinguishable elevations along the cutting margins of the crown.

The specimen was studied at the MFSN using a Wild M3 binocular microscope. Photographs of the individual skeletal elements were sometimes taken in ethanol immersion to enhance the contrast between the specimen and the matrix. When paired elements have different lengths, the mean was used in the calculation of the long bone length ratios. In the drawings of the whole specimen and of details of the specimen, the rock is shown pale grey, the parts reconstructed in resin are dark grey and the skeletal elements are white, unless specified otherwise.

The phylogenetic relationships of *Seazzadactylus venieri* were investigated using the data matrix of Britt et al. (2018). *Seazzadactylus venieri* was added to the version of this data matrix that is inclusive of MCSNB 8950, and the resulting dataset was then used to perform parsimony-based phylogenetic analysis by PAUP 4.0b10 for Microsoft Windows (Swofford, 2002) using the default search parameters plus the instruction hsearch addseq=random nreps=1000 nchuck=100 chuckscore=1 for the heuristic search. The analysis was subsequently performed also by TNT (Goloboff & Catalano, 2016). The matrix contains 93 characters; three are ordered and 90 unordered. The total number of operational taxonomic units is 30 (three outgroup and 27 ingroup). *Macrocnemus bassanii, Postosuchus kirkpatricki* and *Herrerasaurus ischigualastensis* were chosen as outgroup taxa. Nodal support was calculated by TNT using the Bremer function, replicating the analysis and saving all trees up to 10 steps longer than the shortest topologies.

The electronic version of this article in portable document format will represent a published work according to the International Commission on Zoological Nomenclature (ICZN), and hence the new names contained in the electronic version are effectively published under that Code from the electronic edition alone. This published work and the nomenclatural acts it contains have been registered in ZooBank, the online registration system for the ICZN. The ZooBank Life Science Identifiers (LSIDs) can be resolved and the associated information viewed through any standard web browser by appending the LSID to the prefix http://zoobank.org/. The LSID for this publication is: urn:lsid:zoobank.org:pub:5F0C4B84-F39D-436F-93FD-858B323C6A15. The online version of this work is archived and available from the following digital repositories: PeerJ, PubMed Central and CLOCKSS.

## Systematic Palaeontology

Reptilia Laurenti, 1768 sensu Modesto & Anderson (2004)
Diapsida Osborn, 1903
Pterosauria Kaup, 1834
*Seazzadactylus venieri* gen. et sp. nov.
(Figs. 1–6, 7A, 7B, 8, 9A, 9B, 10A–10D, 11–22, 23A and 23B)

2000 a partial skeleton still to be prepared: Dalla Vecchia, p. 229.
2003 *Eudimorphodon*: Dalla Vecchia, p. 25.
2004a *Eudimorphodon*: Dalla Vecchia, p. 48, figs 1 and 5E.
2004b *Eudimorphodon*: Dalla Vecchia, p. 19, fig. 14.
2006 *Eudimorphodon* sp.: Dalla Vecchia, p. 436, fig. 12 left.
2006 *Eudimorphodon*: Fröbisch & Fröbisch, p. 1087.
2008 *Eudimorphodon*: Dalla Vecchia, p. 185, fig. 182.
2009a neither *Eudimorphodon ranzii* nor *Carniadactylus rosenfeldi*: Dalla Vecchia, p. 164.
2010 a distinct taxon (with respect to *Eudimorphodon*): Dalla Vecchia, p. 183.
2012 una specie distinta da *Carniadactylus rosenfeldi*: Dalla Vecchia, p. 185, fig. 8.141.
2013 probably (it) represents a new genus and species: Dalla Vecchia, p. 133.
2014 Genere e specie senza nome: Dalla Vecchia, p. 227, fig. 4.1.164.
2015 a new and still unnamed taxon: Dalla Vecchia and Cau, p. 685, fig. 2H.
2018 a still unnamed taxon with multicusped teeth: Dalla Vecchia, p. 333.

**Zoobank.** urn:lsid:zoobank.org:act:1B567D5D-E9BC-41A0-BA73-04A29F496989; urn:lsid:zoobank.org:act:02CB1E39-1338-49F7-8493-37C474ED7663.

**Etymology.** ‘*Seazza*’ after Seazza Brook where the holotype was found and ‘*dactylus’*, from Greek ‘*daktylos*’ for ‘digit’. The specific name pays hommage to Umberto Venier, who found the specimen.

**Holotype.** MFSN 21545, disarticulated but associated partial skeleton including skull and mandible elements (Figs. 1 and 2).

**Locality and Stratigraphic horizon.** Seazza Brook, Preone municipality, Friuli Venezia Giulia Autonomous Region, Italy; Dolomia di Forni Formation (Alaunian 3- Sevatian, middle-upper Norian).

**Diagnosis.** Non-monofenestratan pterosaur with multicusped dentition and the following apomorphic features: teeth restricted to the rostral half of the body of the premaxilla; deep maxillary process of jugal that tapers to a needle-like point ventrodistally; large foramen in the middle of the jugal body; pterygoid with rostral ramus bent 90° laterally; ectopterygoid caudal to the pterygoid and with recurved lateral (jugal) and caudal processes; multicusped dentition in the dentary and maxilla that includes hexa- and heptacuspid crowns and no fully grown tricuspid teeth; recurved maxillary crowns 1–3 with curvature decreasing from tooth 1 to 3; flared and fan-like scapular blade; small and slender exclamation-mark-shaped pteroid.

## Description

Most of the skeleton was preserved in the slab, but the caudal segment of the vertebral column is missing and only very small portions of the feet are present (Figs. 1 and 2). The most disarticulated part of the skeleton is the vertebral column. The skull is disarticulated, but its elements are closely associated, as are the mandibular rami that are paired and still parallel to one other. The scapulocoracoids are also close and parallel to one other. The bones of the right forelimb are articulated at least up to the wing phalanx 2, whereas the nearly complete left forelimb is slightly disarticulated. Tibiotarsi and femora of both hind limbs are closely associated and parallel to one other. The feet are completely disarticulated and no metatarsals and metatarsal-like phalanges are preserved. Before burial, the carcass probably macerated on a low-energy sea bottom without significative water currents, which prevented bone dispersal.

Comparison with other pterosaur taxa is employed here when it is necessary for the identification of the elements of MFSN 21545; comparison for systematic purposes is reported in the Discussion section.

### Cranial bones

**Figure 3:**
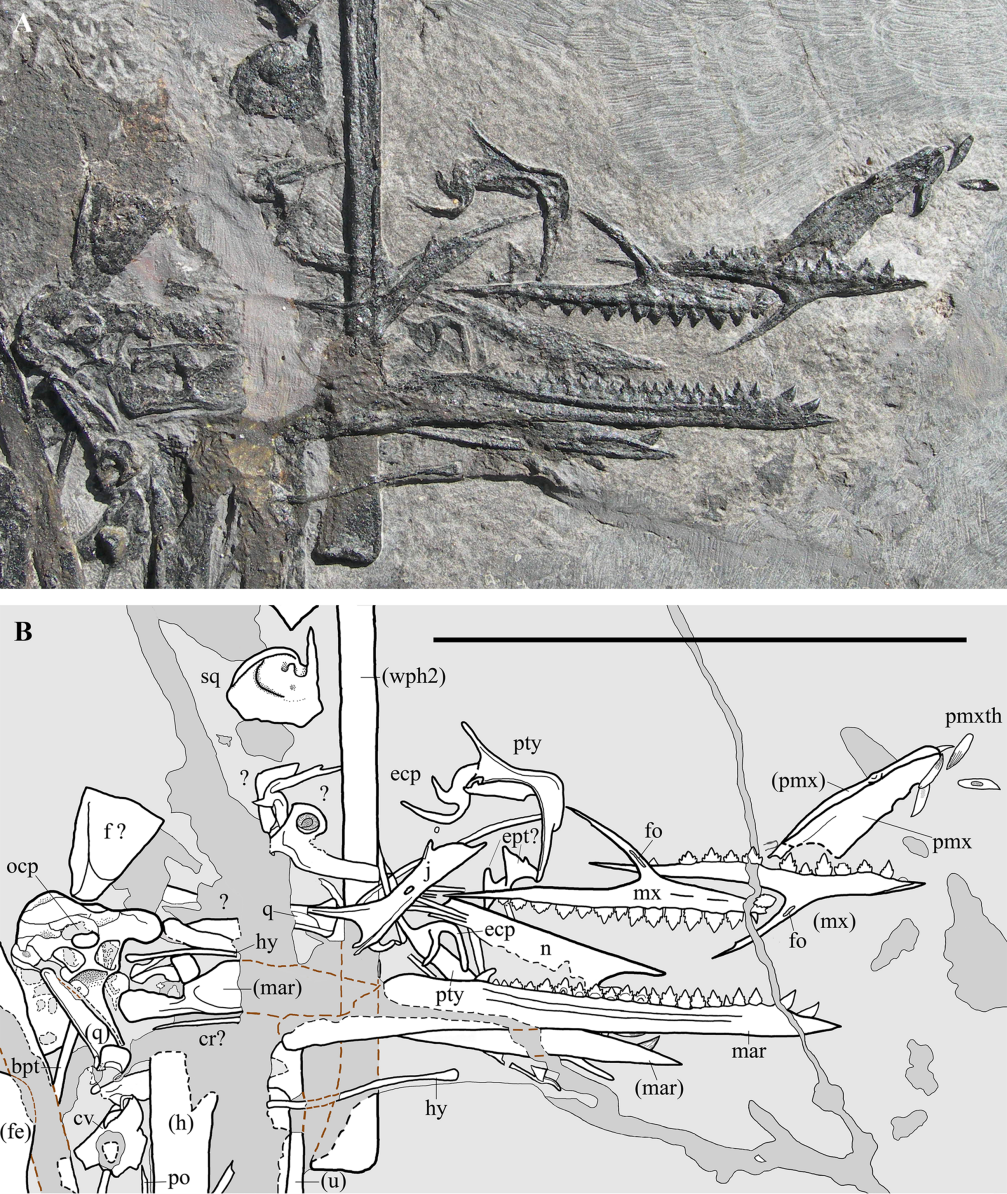
*Seazzadactylus venieri*, MFSN 21545 (holotype), skull and mandible. (A) Photograph; (B) drawing. The postorbital is the only skull bone that is partially outside of the photograph, extending further downwards from the lower left corner. Black dashed lines mark the broken margins of the bones where they can be identified as such; brown dashed lines mark the reconstructed margin of the bones. Abbreviations: bpt, basipterygoid process; cr, cervical rib; cv, cervical vertebra; ecp, ectopterygoid; ept, epipterygoid; f, frontal; fe, femur; fo, foramen; h, humerus; hy, ceratobranchial I (hyoid apparatus); j, jugal; mar, mandibular ramus; mx, maxilla; n, nasal; ocp, occiput; pmx, premaxilla; pmxth, premaxillary teeth; po, postorbital; pty, pterygoid; q, quadrate; sq, squamosal; u, ulna; wph2, wing phalanx 2. Elements in parentheses are from the left side. Scale bar equals 50 mm.

Many skull elements are preserved and can, because of their disarticulated state, be observed in aspects not visible in articulated skulls (Fig. 3). Unfortunately, a wide fracture crosses the caudal part of the skull and some bones, mainly those of the skull roof, were either lost or incompletely preserved.

**Figure 4:**
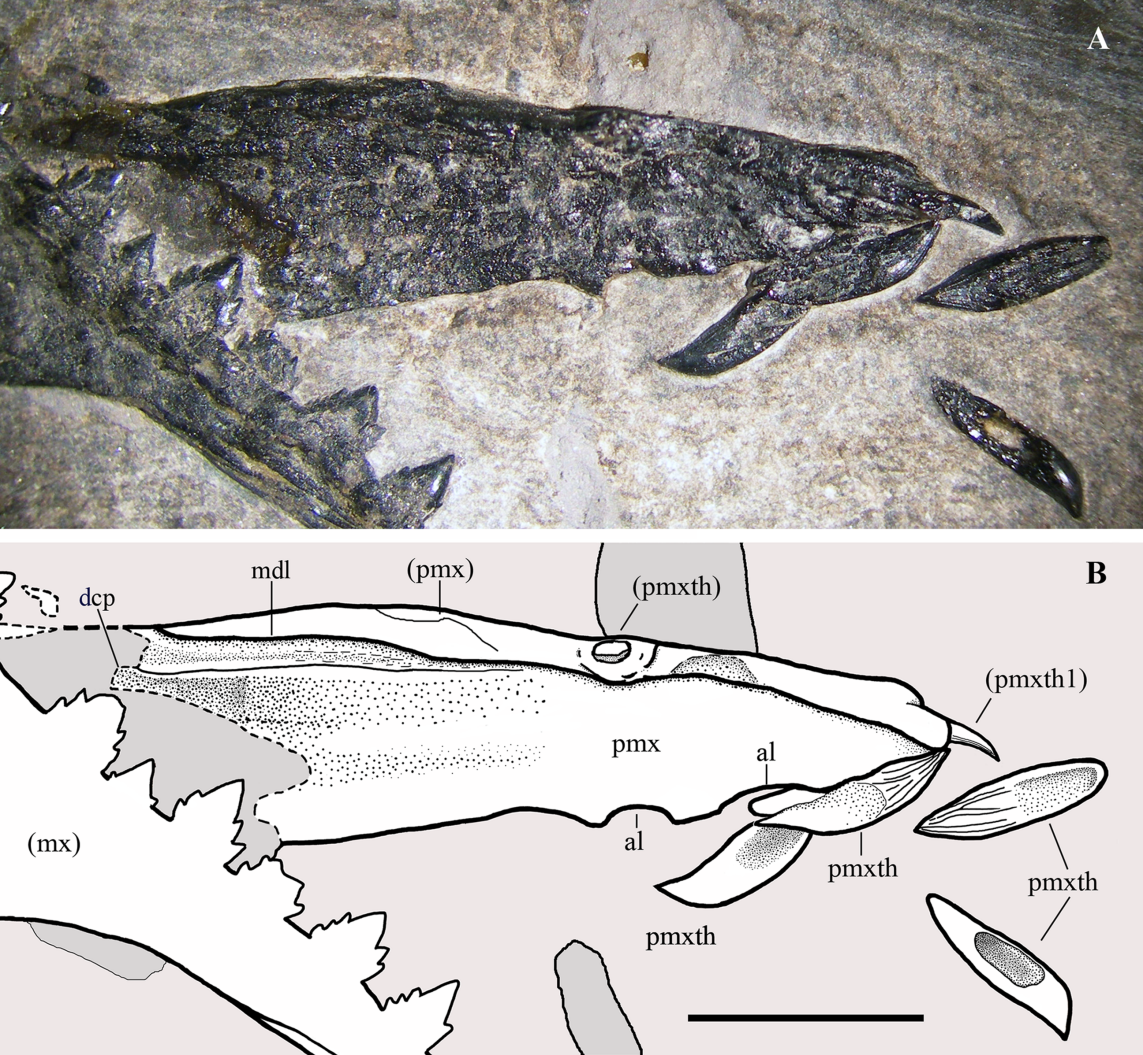
*Seazzadactylus venieri*, MFSN 21545 (holotype), premaxillae. (A) Premaxillae in right dorsolateral view, with four premaxillary teeth displaced from their alveoli; (B) drawing. The broken margins of the bones are marked by dashed lines. Abbreviations: al, alveolus; dcp, dorsocaudal (frontal) process; mdl, midline (suture between the two premaxillae); mx, maxilla; pmx, premaxilla (body); pmxth, premaxillary tooth. Elements in parentheses are from the left side. Scale bar equals five mm.

**Premaxillae.** The premaxillae (Fig. 4) are fused but their suture is still evident. Both dorsal and lateral sides of the right premaxilla are exposed, whereas only the dorsal portion of the left one is visible. As exposed, the premaxillae are very narrow and long (18.2 mm long [excluding the apical tooth] and six mm maximum width) and slightly taper rostrally. They are broken anterior to the rostral margin of the external naris. The rostral tip of the joint premaxillae is blunt. The premaxillary body is low in lateral view. The first tooth of the left premaxilla is still in situ and points forwards, whereas four teeth have dropped out of their alveoli. Only two large distal alveoli are fully exposed along the ventral margin of the right premaxilla, because two displaced teeth conceal the mesial alveoli. Teeth occur only in the rostral half of the premaxillary body.

**Figure 5:**
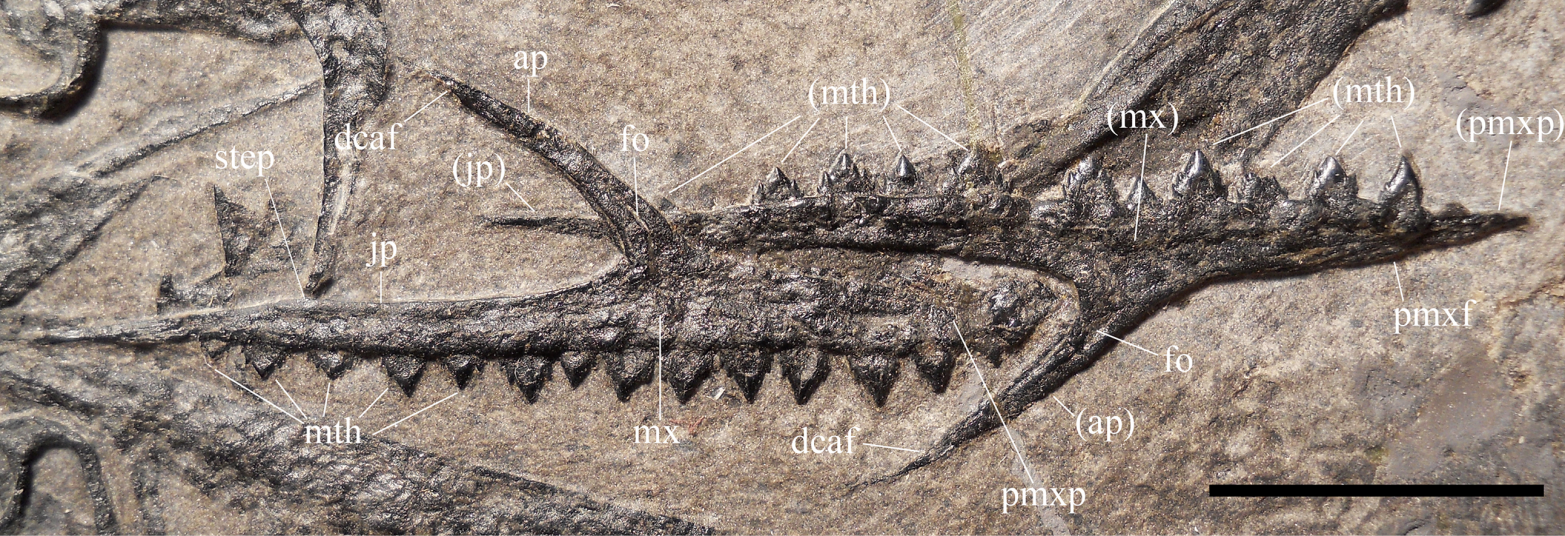
*Seazzadactylus venieri*, MFSN 21545 (holotype), maxillae. Photograph. Abbreviations: ap, ascending process; dcaf, dorsocaudal articular facet on the ascending process; fo, neurovascular foramen; jp, jugal process; mth, maxillary tooth; mx, maxilla; pmxf, facet for the maxillary process of premaxilla; pmxp, premaxillary process. Elements and processes in parentheses are from the left side. Scale bar equals 10 mm.

**Maxillae.** Both maxillae (Fig. 5) show their lateral side, due to the upside-down flipping of the left maxilla. The left maxilla is complete and is 33.2 mm long. The premaxillary process of the right maxilla is rostrally damaged by a fracture and its rostral end is covered by matrix and the displaced left maxillary tooth 8. The maxilla is a triradiate element with slender processes that taper distally to a point. The jugal process is the longest, whereas the premaxillary and the ascending processes are of about the same length (the premaxillary process is 55% of the length of the jugal process). The ascending process slopes caudally at 145° and is slightly arched. It tapers apically to a narrow point and is relatively short; apically, it has a long articular surface along the caudal side, like that for the lacrimal in the reconstruction of the skull of *Scaphognathus crassirostris* by Wellnhofer (1975b, fig. 34a). A short and deep longitudinal groove on the lateral side of the expanded base of the ascending process (Fig. 5) probably corresponds to the large neurovascular foramen observed there in the maxilla of *Preondactylus buffarinii* and *Caelestiventus hanseni* (see Britt et al., 2018). There is no trace of a maxillary contribution to an antorbital fossa. The premaxillary process has a triangular and distally tapering outline in lateral view. The dorsal margin of the premaxillary process is not straight but slightly angled midway where a slit-like articular facet for the maxillary process of the premaxilla starts. Therefore, the maxillary process of the premaxilla bordered the external naris rostroventrally. The jugal process is lower than the premaxillary process; it tapers distally, but tapering is minimal in the proximal segment and increases in correspondence of a change in inclination of the dorsal margin (the ‘step’ in Fig. 5). The segment caudal to this change in inclination is the portion that articulated with the jugal.

The left maxilla preserves 11 teeth in situ. The first tooth is missing, probably because of the damage to the tip of the premaxillary process; tooth 8 slipped out of its alveolus and covers the tip of the premaxillary process of the right maxilla; tooth 13 is represented by an empty alveolus. Therefore, this maxilla has 14 tooth positions. The right maxilla has 14 teeth in situ. Comparison with the left maxilla suggests that the first tooth of the series is tooth 1.

**Nasal.** An elongate (22 mm long), flat and thin bone is preserved between the maxillae and the mandibular rami (Fig. 3). Because of its position and morphology, it is tentatively identified as a nasal. Its rostral extremity tapers to a premaxillary process bounding dorsally a rostral notch corresponding to the dorsocaudal margin of the external naris. The maxillary process is overlapped and concealed by the right mandibular ramus and its dentition. The body of the nasal is straight and its dorsal margin is rectilinear. Its caudal end appears to be squared, but the caudoventral corner is concealed by other bones. Its ventral or ventrolateral margin is irregular and probably not the actual margin of the element but an artefact of preparation on a rather thin bone. As for its shape, size and position, the element could only be alternatively identified as a palatine. However, if it were the palatine, the notch corresponding to the choana should be situated caudally (see Ősi et al., 2010, figs. 1-2). This would imply an unlikely 180° rotation of the bone. The identification as a detached and drifted palatal plate of a maxilla (Ősi et al., 2010, fig. 8) seems also to be unlikely.

**Frontal.** A large fragment of a broad bone preserved dorsal to the occiput is tentatively identified as part of a frontal or of the fused frontals (Fig. 3). It does not show any crests or ridges and gives no information about the morphology of the frontals.

**Figure 6:**
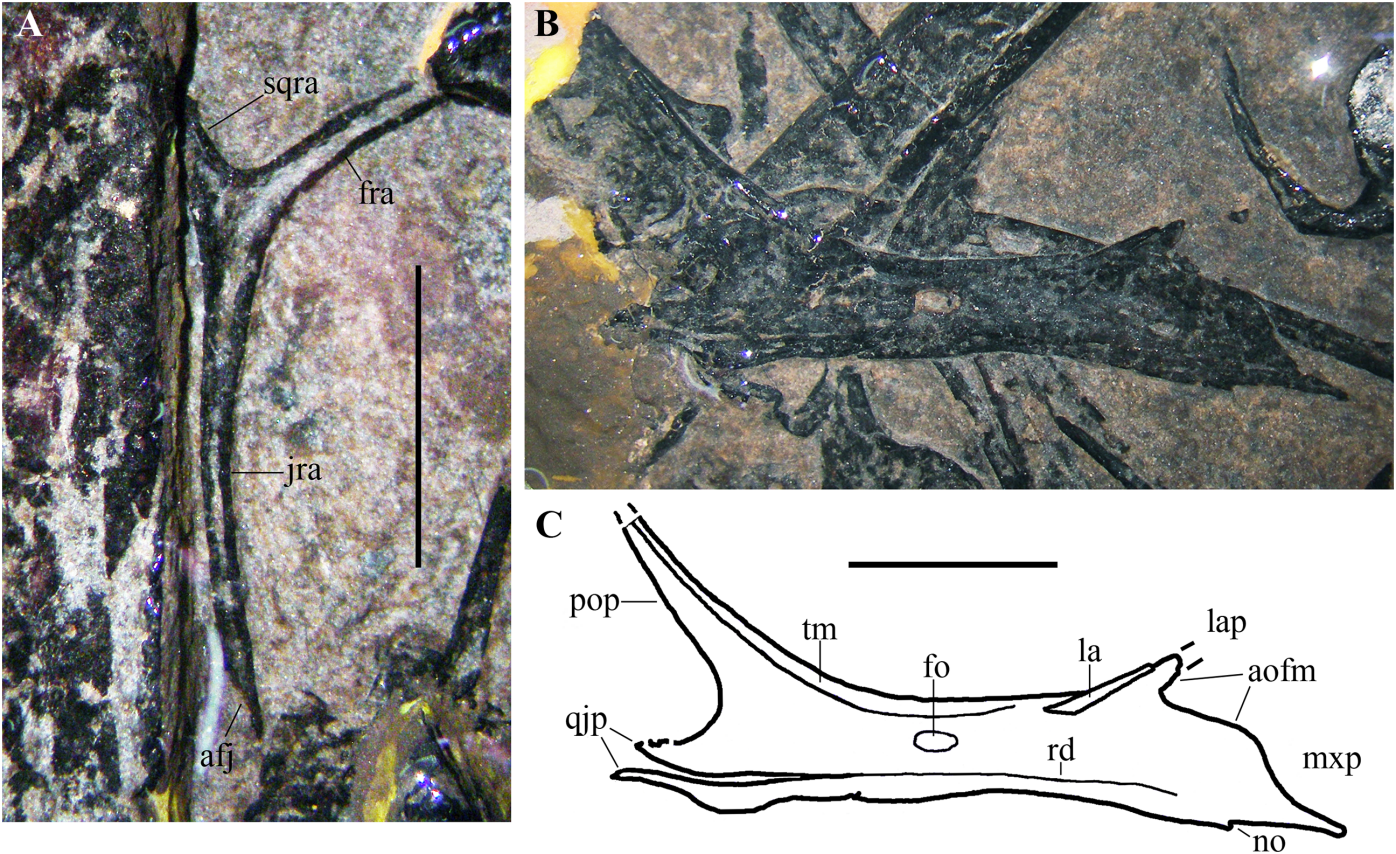
*Seazzadactylus venieri*, MFSN 21545 (holotype), postorbital and jugal. (A) Postorbital; (B) right jugal, lateral view; (C) drawing of (B). Photographs were taken under ethanol immersion. Abbreviations: afj, articular facet for the jugal; aofm, antorbital fenestra margin on the jugal; fo, foramen; fra, frontal ramus of postorbital; jra, jugal ramus of postorbital; la, lacrimal; lap, lacrimal process of jugal; mxp, maxillary process of jugal; no, notch; pop, postorbital process of jugal; qjp, quadratojugal process of jugal; rd, ridge; sqra, squamosal ramus of postorbital; tm, thickened margin. Scale bar equals five mm.

**Postorbital.** The postorbital is a triradiate (Y-shaped; Fig. 6A) and a very slender element. It closely resembles the postorbitals of *Carniadactylus rosenfeldi* (MPUM 6009) and *Austriadraco dallavecchiai* (see Dalla Vecchia, 2018, fig. 3A-B), but it is even more gracile. Its length from the distal extremity of the jugal ramus to the extremity of the exposed portion of the frontal ramus is 10.5 mm. Only the proximal part of the squamosal ramus is visible because the rest is covered by the left humerus. The slender frontal ramus is slightly curved with a rostroventral concavity; its distal end is covered by a cervical vertebra and the right tibia. The exposed portions of the squamosal and frontal rami form an angle of about 85°. This indicates that the upper temporal fenestra had a relatively acute ventrolateral margin (this angle is about 70° in *Carniadactylus rosenfeldi* and about 80° in *Austriadraco dallavecchiai*, but these values are based on more complete squamosal rami; Dalla Vecchia, 2018). The long and very slender jugal ramus is curved with rostral concavity and tapers distally where there is a caudoventral facet for the articulation with the postorbital process of the jugal. Frontal and jugal rami border the caudal part of the broad orbit; their curvature and length, united to those of the postorbital process of the jugal, suggest the presence of a circular and very large orbit.

**Figure 7:**
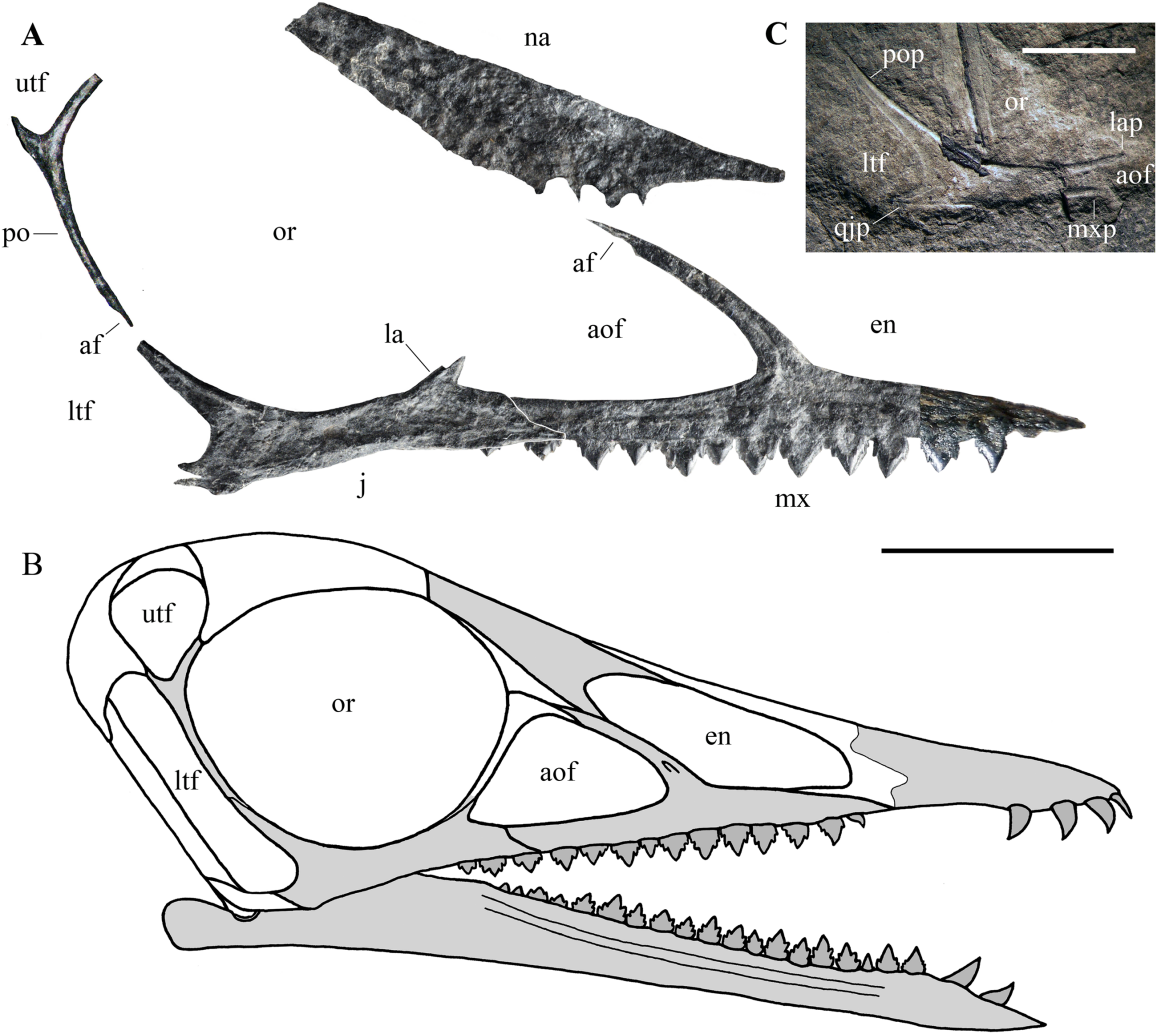
*Seazzadactylus venieri*, MFSN 21545 (holotype), assembly of skull bones and skull reconstruction. (A) assembly of the jugal, maxilla, postorbital and presumed nasal with the jugal and maxilla articulated to obtain a continuous ventral margin of the antorbital fenestra, but concealing the last two maxillary teeth; (B) tentative skull reconstruction (the preserved bones are in grey colour); (C); the jugal of the holotype of *Austriadraco dallavecchiai* (mirrored), for comparison. In (A), the right jugal and maxilla are used in the assembly of the bones; the postorbital may be the right in lateral view or the left in medial view; the presumed nasal may be the left or the right. In (A), the incompletely exposed rostral end of the premaxillary process of the right maxilla was integrated with the rostral end of the premaxillary process of the left maxilla (colour of the part from the left maxilla is darker to show this integration). In (A), the ventral margin of the presumed nasal is irregular because it is covered by the right mandibular ramus in the specimen. Abbreviations: af, articular facet; aof, antorbital fenestra; en, external naris; j, jugal; la, lacrimal; lap, lacrimal process of the jugal; ltf, lower temporal fenestra; mx, maxilla; mxp, maxillary process of the jugal; na, nasal; or, orbit; po, postorbital; pop, postorbital process of the jugal; qjp, quadratojugal process of the jugal; utf, upper temporal fenestra. Scale bar is 10 mm in (A) and five mm in (C).

**Jugal.** The right jugal is exposed in lateral view (Figs. 6B and 6C) and is tetraradiate as in many other basal pterosaurs (Wellnhofer, 1978, 2003; Dalla Vecchia, 2014). It is not fused with the maxilla, postorbital and quadratojugal. Its length is 17 mm from the caudal extremity of the quadratojugal process to the rostral end of the maxillary process. The postorbital process is much longer than the other processes; it is slender and tapers distally. Although the distal termination of this process is broken and is not preserved, its maximum length can be estimated based on the convergence of its cranial and caudal margins and comparison with the jugal process of the postorbital (see Fig. 7A). The postorbital process is nearly straight and caudally inclined at about 130° with respect to the axis of the jugal body. Its orbital margin is thickened. The maxillary process is ventrally deflected at about 20° with respect to the axis of the jugal body. It is deep proximally where it contributes to the caudal end of the ventral margin of the antorbital fenestra and tapers to a needle-like point distally. A very small notch is present along the ventral margin. The lacrimal process is rostrodorsally directed and forms an angle of about 35° with the axis of the jugal body. This process is very short, appearing as a triangular spur. It is partially overlapped dorsally by a rod-like bone. Comparison with *E. ranzii* (see Wild, 1979, fig. 1), *Carniadactylus rosenfeldi* (see Dalla Vecchia, 2018, fig. 2) and *Raeticodactylus filisurensis* (see Stecher, 2008, fig. 6) suggests that this latter element is part of the damaged lacrimal. This suggests also that the short lacrimal process might be incomplete and was longer originally, but its relatively narrow base and tapering margins indicate that it could not be much longer than preserved. A short, triangular process of the jugal is damaged distally and forms the ventral margin of the lower temporal fenestra. This process is clearly separated from a ventral strip of bone by a gap, but the gap becomes a ridge parallel to the ventral margin of the jugal rostrally (Figs. 6B and 6C). Comparison with the 3D-ct scans of the jugal of *Caelestiventus hanseni* (see Britt et al., 2018, fig. 3) suggests that this strip of bone in MFSN 21545 belongs to the thin ventral part of the jugal and is not the quadratojugal. The strip is broken and partly detached in MFSN 21545 because of the crushing of the jugal on other bones. Consequently, the quadratojugal process of the jugal is made of the triangular process forming the ventral margin of the lower temporal fenestra plus the caudal portion of the detached strip of bone and is damaged distally.

The jugal body is rectangular in lateromedial view and is slightly constricted dorsoventrally in the middle. The orbital margin is thickened. A large elliptical foramen pierces the bone at the point of minimum depth.

**Cranial fenestrae.** The shape of the cranial openings can be reconstructed by returning the preserved skull elements to their original position. The articulation between the jugal and maxilla appears to differ among non-monofenestratan pterosaurs. In *Dimorphodon macronyx* (see Sangster, 2003, fig. 2.9) and *Caelestiventus hanseni* (F.M. Dalla Vecchia, 2018, personal observation) the jugal overlaps the jugal process of the maxilla laterally, whereas it overlaps the jugal process of the maxilla dorsally in *E. ranzii* (see Wild, 1979, fig. 1) and *Carniadactylus rosenfeldi* (see Dalla Vecchia, 2018, fig. 2). When the jugal and maxilla of MFSN 21545 are returned to their articular position with the jugal that overlaps the jugal process of the maxilla dorsally (Fig. S3A), the last two maxillary teeth lie below the jugal and the resulting antorbital fenestra is very long and has a ‘step’ in its ventral margin that is not observed in any other pterosaur. When the jugal and maxilla are returned to their articular positions with the jugal being overlapped medially by the jugal process of the maxilla, the overlap ends rostrally where the change in inclination of the dorsal margin of the jugal process of the maxilla occurs (the ‘step’ in Fig. 5), as suggested by analogy with the maxillojugal of *Caelestiventus hanseni* (F.M. Dalla Vecchia, 2018, personal observation). However, two options exist. In the first, the last three maxillary teeth lie below the maxillary process of the jugal and are not covered labially by it, but the ventral margin of the antorbital fenestra possesses an unusual ‘step’ similar to that obtained by the dorsoventral overlap (Fig. S3B). In the second option, the jugal and maxilla overlap to form a ‘smooth’ (i.e. ‘step’-free) ventral margin of the antorbital fenestra (as is the case in other pterosaurs; see *Raeticodactylus filisurensis* in Fig. S4), the maxillary process of the jugal entirely covers the last tooth and partly also the penultimate tooth (Fig. 7A). This articulation between jugal and maxilla resembles that of *Dimorphodon macronyx* but the point of the maxillary process occurs ventrally in *Seazzadactylus venieri* instead of dorsally (cf. Sangster, 2003, fig. 2.9). The labial overlapping of the last two maxillary teeth could be a consequence of the crushing and flattening of the rostroventral margin of the jugal. This second option is chosen here in the assembly of the jugal, maxilla, postorbital and presumed nasal (Fig. 7A), and in the skull reconstruction (Fig. 7B). With this articulation, the axis of the jugal is oriented dorsocranially-ventrocaudally and the ventral margin of the skull at the articulation with the mandible is curved down caudally.

In the assembly, the jugal, maxilla, and postorbital articulate smoothly (Fig. 7A), but the placement of the presumed nasal is somewhat problematic. The bone appears to be of excessive size for a nasal, but it is now flattened, whereas it was dorsolaterally arched in vivo and thus would have been less exposed laterally than appears in Fig. 7A. Caudally, the nasal probably overlapped the frontal and extended over the orbit as in other pterosaurs. However, its exact position cannot be established because the rostroventral (maxillary) process is concealed by the right mandibular ramus. How it articulated with the maxilla is therefore unknown. The ascending process of the maxilla possesses a caudal articular facet along its apical part. This facet likely received the lacrimal as in the reconstructions of the skulls of *E. ranzii*, *Carniadactylus rosenfeldi*, *Raeticodactylus filisurensis*, *Campylognathoides liasicus*, *Dorygnathus banthensis* and *Scaphognathus crassirostris* (Wellnhofer, 1978; Sangster, 2003). The rostroventral process of the nasal articulates dorsally with the ascending process of the maxilla in the reconstructions of these taxa and in those of *Rhamphorhynchus muensteri* and *Angustinaripterus longicephalus* (see Sangster, 2003). In the tentative reconstruction of the skull (Fig. 7B), the presumed nasal of MFSN 21545 is placed in a rostral position based on this dorsal articulation of the nasal with the maxilla. The original slope of the nasal is unknown, as also are the length and orientation of the caudal processes of the premaxilla. Consequently, the reconstructed shape and size of the external naris are tentative. Although most of the lacrimal is not preserved, the inclination of the lacrimal process of the jugal and the ascending process of the maxilla show that the antorbital fenestra was large and shaped like an isosceles triangle (Figs. 7A and 7B), more similar to the large and oval antorbital fenestra of *Raeticodactylus filisurensis* (see Stecher, 2008; Fig. S4), than the smaller and D-like antorbital fenestra of *E. ranzii* (see Wild, 1979). The orbit is very large and sub-circular; as in many other basal pterosaurs, it is the largest skull opening. The shape of the lower temporal fenestra cannot be known exactly because the quadratojugal is not preserved, but the lengths of the postorbital process of the jugal and of the jugal process of the postorbital indicate that it was very long caudodorsally to rostroventrally and probably rather narrow. The lateroventral margin of the upper temporal fenestra is V-shaped as in *Carniadactylus rosenfeldi*, *Austriadraco dallavecchiai* and *Campylognathoides liasicus*. As in the reconstructions of the skull of *Carniadactylus rosenfeldi* by Wild (1979, fig. 2), the upper temporal fenestra had probably the outline of an inverted tear-drop.

**Squamosal.** Part of the left squamosal appears still to be connected to the left side of the occiput, but is intensely deformed and broken because of strong crushing. A large fragment lateral to the left paroccipital process bears a shallow and rimmed, elliptical socket that is 1.25 mm long, which corresponds in size with the proximal articular head of the quadrate. This socket could be the cotyle for the quadrate. A rounded bone with a pointed process, located close to the left wing phalanges 2 and 3 (Fig. 3), could be a disarticulated, displaced and strongly crushed right squamosal. Identification is based on the size and shape of the element, in particular the shape of its process, which resembles the squamosal descending flange that overlaps the caudal or caudolateral surface of the quadrate in *Carniadactylus rosenfeldi* (see Wild, 1979, fig. 2), *Dorygnathus banthensis* (see Padian, 2008a, figs. 6 and 16), *Campylognathoides liasicus* (see Wellnhofer, 1974, fig. 2; Padian, 2008b, figs. 4 and 6), *Scaphognathus crassirostris* (see Wellnhofer, 1975b, figs. 33 and 34a) and in many other pterosaurs (e.g. Wellnhofer, 1978, figs. 2, 4 and 5; Codorniú, Paulina-Carabajal & Gianechini, 2016, figs. 1D and 8).

**Figure 8:**
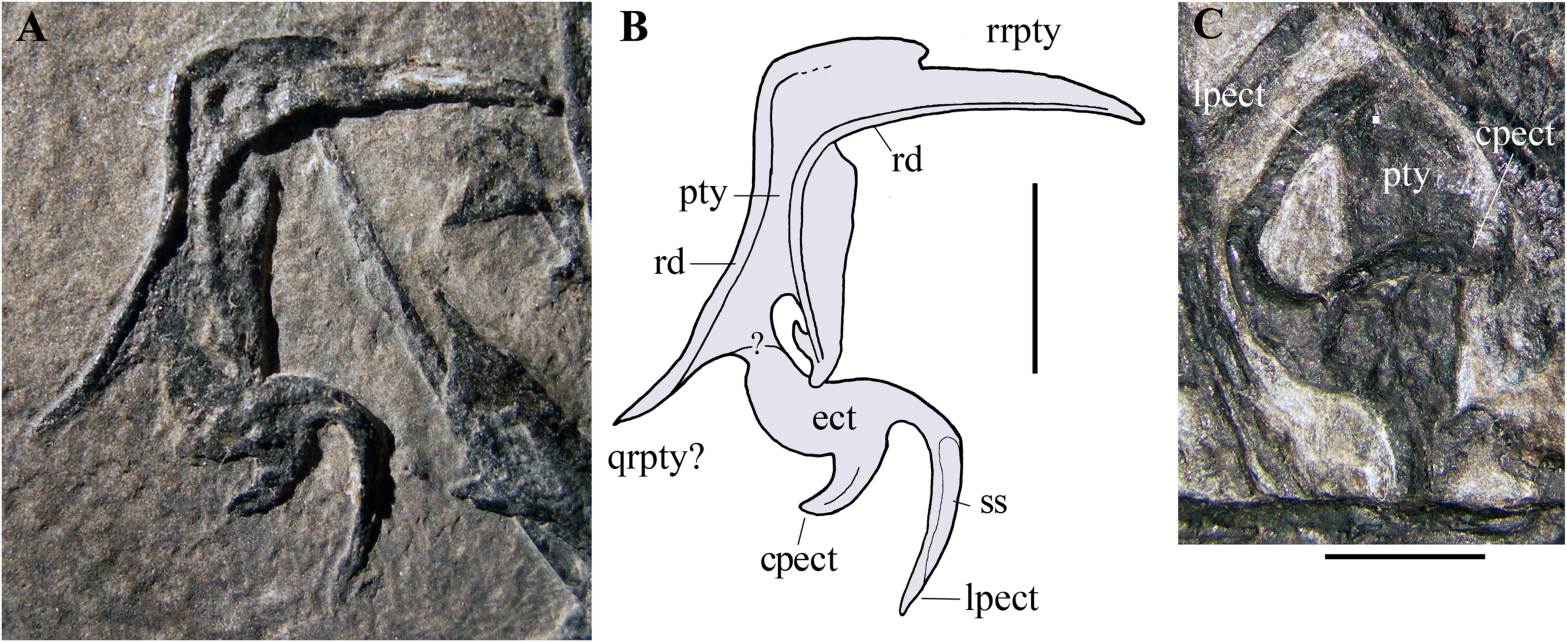
*Seazzadactylus venieri*, MFSN 21545 (holotype), pterygoid and ectopterygoid. (A) Right pterygoid and ectopterygoid in palatal view; (B) drawing of (A); (C) left pterygoid and ectopterygoid. Abbreviations: cpect, caudal (pterygoid) process of the ectopterygoid; ect, ectopterygoid; lpect, lateral (jugal) process of the ectopterygoid; pty, pterygoid; qrpty, quadrate ramus of the pterygoid; rd, ridge; rrpty, rostral ramus of the pterygoid; ss, sutural surface. Scale bar equals five mm in (B) and three mm in (C).

**Pterygoid and ectopterygoid.** A skeletal element with four slender and pointed processes (Figs. 8A and 8B) is preserved isolated just dorsal to the right maxilla and the right jugal. Assuming that the element retains its anatomical orientation, its caudal portion bears two paired, recurved, and caudally directed processes at its caudal end and a third, straight and caudolaterally or caudomedially directed process in a more rostral position. The outer margin of this third process is thickened and ridge-like; this ridge extends along the margin of the rectangular main body of the skeletal element. The fourth and rostral process is actually a 90° bend in the bone and tapers distally. A ridge originating at the proximal part of the rostral process extends longitudinally along the main body of the bone. A partially exposed skeletal element with the same recurved caudal processes occurs between the right jugal and the right mandibular ramus (Fig. 8C). However, the two recurved processes are differently oriented with respect to their homologues on the other skeletal element, suggesting that they may belong to a skeletal element that is tightly connected but distinct from the main body and not fused to it. The possible boundary between these two elements is indicated in Fig. 8B.

Their position with respect to the maxillae, right jugal and mandibular rami, and their morphology, suggest that these bones are palatal elements. Because of their position and size, they are plausibly the pterygoids with the ectopterygoids preserved in dorsal or palatal view (e.g., Ősi et al., 2010, fig.1 and 8B). They are probably flattened by crushing and the various processes may lie artificially in the same plane. Their right-left polarity cannot be unambiguously established based on their position alone, but the completely exposed bone is probably the right one in palatal view (see below).

The morphology of these elements is unlike that of the pterygoid-ectopterygoids of other basal pterosaurs, namely *Carniadactylus rosenfeldi* (see Dalla Vecchia, 2009a, fig. 2A); *Dorygnathus banthensis* (see Ősi et al., 2010, figs. 2, 6B, and 8B), *Campylognathoides liasicus* (see Wellnhofer, 1974, figs. 2 and 4; Padian, 2008b, pl. 7/figs 2 and 5, fig. 8), *Cacibupteryx caribensis* (see Gasparini, Fernandez & De La Fuente, 2004, fig. 2D), *Scaphognathus crassirostris* (see Wellnhofer, 1975b, figs. 33a and 34b; Bennett, 2014, fig. 5B) and *Rhamphorhynchus muensteri* (see Wellnhofer, 1975a, fig. 3d; Ősi et al., 2010, figs. 1C-D and 9A). The partially exposed pterygoid of *Dimorphodon macronyx* also appears to be different from that of MFSN 21545 (Sangster, 2003, fig. 2.9). Particularly, the ectopterygoids of those pterosaurs occur in a rostral position with respect to the pterygoid. None of these other taxa has a rostral process that is bent at 90°. The paired recurved processes of the ectopterygoid resemble those of the ectopterygoid of the theropod dinosaur *Allosaurus fragilis* (see Madsen, 1976, pls. 2B and 10D) in respect of their overall morphology and their position relative to that of the pterygoid, although the ectopterygoid of this dinosaur is proportionally larger than that of MFSN 21545. The pterygoid of *Allosaurus fragilis* is straight in palatal view (Madsen, 1976, pls. 2B) unlike that of MFSN 21545. The pterygoid-ectopterygoid of the basal pterosaur *Sordes pilosus* (the paratype PIN 2470 1B, F.M. Dalla Vecchia, 2018, personal observation on photographs) differs from those of other pterosaurs reported in literature and may be like that of MFSN 21545, including in regard to the 90° bending of the rostral process of the pterygoid. Unfortunately, the palate of *Sordes pilosus* was never described and figured in detail.

The tentative identification of the processes of the pterygoid-ectopterygoid of MFSN 21545 in Fig. 8 is essentially based on the pterygoid-ectopterygoid of *Allosaurus fragilis*. The longer and more slender of the two recurved processes of the ectopterygoid has a long facet that could represent its sutural facet with the jugal (Figs. 8A and 8B), and can therefore be interpreted as the jugal process, which was originally directed laterally and forming the rostral margin of the subtemporal fenestra and the caudal margin of the suborbital fenestra. Consequently, the other recurved process is the caudal process of the ectopterygoid, which overlapped the pterygoid laterally in *Allosaurus fragilis* (Madsen, 1976, pl. 2); if so, the ectopterygoid would be somewhat displaced from its anatomical articulation with the pterygoid. The rostral process of the pterygoid would be a laterally bent palatine ramus, whereas the straight caudal process would be the quadrate ramus.

Two thin and paired bones occurring between the two pterygoids and partly overlapped by the jugal process of the right maxilla (Fig. 3) may be tentatively identified as the epipterygoids.

**Figure 9:**
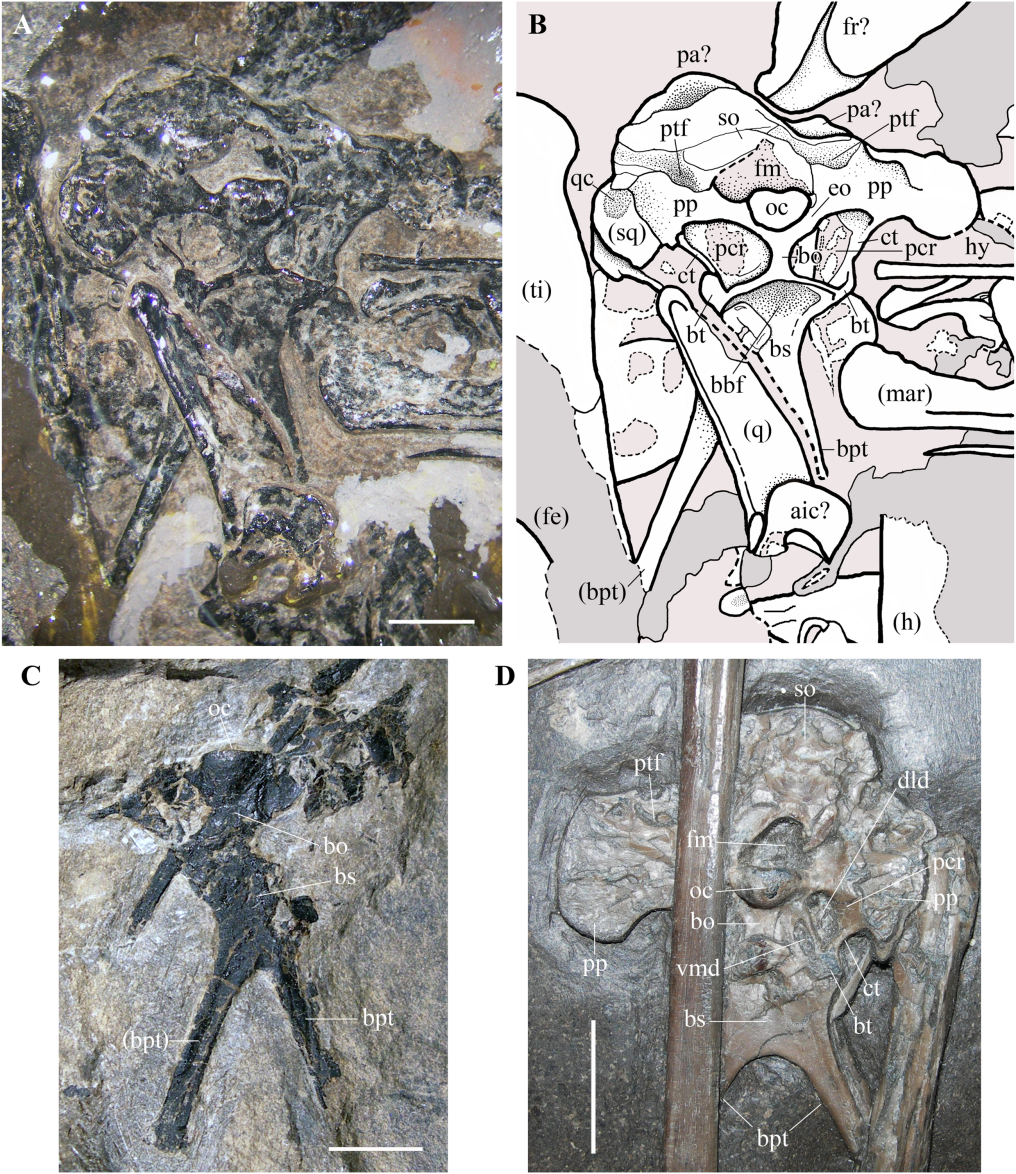
*Seazzadactylus venieri*, MFSN 21545 (holotype), occiput and basicranium in caudal view and comparison. (A) MFSN 21545 (photograph taken under ethanol immersion); (B) MFSN 21545, drawing; (C) holotype of *Austriadraco dallavecchiai* (BSP 1994 I 51); (D) *Dorygnathus banthensis* (SMNS 50164). The broken margins of the bones (where they can be identified as such) are marked by dashed lines. Abbreviations: aic, atlas intercentrum; bbf, basioccipital-basisphenoid fossa; bo, basioccipital; bpt, basipterygoid processes of the basisphenoid; bs, basisphenoid; bt, basal tuber; ct, crista tuberalis; dld, dorsolateral depression; eo, exoccipital; fe, femur; fm, foramen magnum; fr, frontal; h, humerus; hy, ceratobranchial I (hyoid apparatus); mar, mandibular ramus; oc, occipital condyle; pa, parietal; pcr, paracondylar recess; pp, paroccipital process; ptf, posttemporal fenestra (closed); q, quadrate; qc, cotyle for the quadrate on the squamosal; so, supraoccipital, sq, squamosal; ti, tibiotarsus; vmd, ventromedial depression. Elements in parentheses are from the left side (when it was possible to distinguish between right and left elements). Scale bar equals three mm in (A) and (C), 10 mm in (D).

**Quadrate.** The left quadrate is exposed in caudomedial view. It is slightly shifted craniomedially from its anatomical position and overlaps the basisphenoid (Figs. 9A and 9B). The right quadrate is partly preserved and is rotated 90° counter-clockwise in the plane of the occiput from its anatomical position. In caudomedial view, the quadrate is dorsoventrally elongate and strap-like as in other non-pterodactyloid pterosaurs. The proximal portion tapers to a small and rounded articular condyle. The shaft has a straight and thickened lateral margin. The thin and broad medial lamella is partly preserved in the left quadrate. The distal portion with the mandibular condyle and the pterygoid ramus is covered or poorly preserved in both elements.

**Braincase.** The trapezoidal occiput is exposed in caudal view (Figs. 9A and 9B). Unlike the remaining part of the skull, it is not disarticulated, suggesting that the bones forming it were firmly connected. The exposure and overall morphology of this part of the skull resemble those of the holotype of *Carniadactylus rosenfeldi* (see Dalla Vecchia, 2009a, fig. 2A). The occipital condyle is 2.35 mm wide and 1.8 mm high, kidney-shaped and convex. It is comparatively larger with respect to the condyles in pterodactyloids, which have occipital condyles with a rounded outline (e.g., Wellnhofer, 1985, fig. 34; Bennett, 2001, figs. 8-9). There are no visible sutures between the condyle and the basioccipital and between the condyle and the exoccipitals, with the result that the contributions of these bones to the condyle are unclear. The foramen magnum can be identified above the occipital condyle, but its size and outline are affected by crushing. The foramen magnum is bordered dorsally and laterally by the supraoccipital, which is strongly crushed, and its margins cannot be identified with confidence. Portions of the left squamosal and parietals are probably present (Figs. 9A and 9B), but they are strongly crushed and their outlines are unclear. The paroccipital processes project lateral to the occipital condyle, expanding at their lateral extremities. The dorsoventrally narrow portions of the processes that border the foramen magnum ventrally are probably formed by the exoccipitals as in other pterosaurs (e.g. *Rhamphorhynchus muensteri*, Wellnhofer, 1975a, fig. 4a; Padian, 1984, fig. 2), but sutures between the exoccipitals and opisthotics cannot be identified.

The posttemporal fenestrae, which are present in all pterosaurs (e.g., Wellnhofer, 1975a, fig. 4a; Wellnhofer, 1985, fig. 34; Kellner & Tomida, 2000, fig. 9; Bennett, 2001, fig. 9; Codorniú et al., 2016, fig. 1c) cannot be identified dorsal to the paroccipital processes of MFSN 21545, but they might have been closed by the strong compression and crushing that affected the skull. The foramina for the caudal middle cerebral vein, which are reported in *Allkaruen koi* (see Codorniú et al., 2016, fig. 1c) and *Rhamphorhynchus muensteri* (see Wellnhofer, 1975a, fig. 4a) cannot be identified in *Seazzadactylus venieri*.

The basioccipital is hourglass-shaped, very narrow transversely, and much expanded at its ventral boundary with the basisphenoid. The basioccipital and basisphenoid are fused to one another without an apparent suture. The left basal tuber is more developed than the right one, but it is less robust than the basal tubera of *Allkaruen koi* (see Codorniú et al., 2016, fig. 1c). Like the holotype of *Carniadactylus rosenfeldi*, MFSN 21545 has large D-shaped to drop-shaped depressions that are each bordered by the basioccipital medially, the basisphenoid ventrally and the paroccipital processes dorsally (Figs. 9A and 9B). Each depression is bordered laterally by a thin crista tuberalis, which is possibly the ventral ramus of the opisthotic fused to the basal tubera (Gower & Weber, 1998). Plausibly, those depressions were originally deeper rostrocaudally in both specimens before the strong crushing of the skulls and contained one or more foramina that were closed and concealed by crushing. Dalla Vecchia (2009a, fig. 2) reported this depression as the ‘fossa with the vagus foramen’ in *Carniadactylus rosenfeldi*, while it is referred to as paracondylar recess by Codorniú et al. (2016) in the uncrushed skull of *Allkaruen koi*, a term that is adopted here. The paracondylar recess of *Allkaruen koi* is comparatively smaller than those of the two Italian taxa and is mostly occupied by a very large foramen (referred to as the metotic foramen for the exit of nerves IX-XI by Codorniú et al., 2016). A much smaller foramen occurs at the medial margin of the recess in *Allkaruen koi* and is considered to be the foramen for nerve XII (Codorniú et al., 2016, fig. 1c). *Rhamphorhynchus muensteri* has an undivided and very large foramen in the paracondylar recess (Wellnhofer, 1975a, fig. 4a; Padian, 1984, fig. 2B) that can be considered a metotic foramen (Gower & Weber, 1998). The paracondylar recess of *Dorygnathus banthensis* (SMNS 50164; Fig. 9D) is different: it is crossed by a septum that divides it into two large and deep depressions. The dorsolateral depression (as preserved, but in the uncrushed skull was probably somewhat caudolateral) is twice the size of the ventromedial one. Both depressions plausibly contained foramina and represent a divided metotic foramen. Therefore, the larger dorsolateral depression may contain the jugular or vagus foramen transmitting the cranial nerves X, XI (if present), and possibly IX and the jugular vein, whereas the ventromedial depression may contain the fenestra pseudorotunda (for the attachment of a secondary tympanic membrane) and possibly the foramen for the nerve IX (Gower & Weber, 1998).

The paracondylar recess of *Carniadactylus rosenfeldi* is undivided (Dalla Vecchia, 2009a, fig. 2). The condition of the paracondylar recess of *Seazzadactylus venieri* is not immediately clear because the left recess appears to differ from the right one (Figs. 9A and 9B). No bone septum divides the left recess, while a thick bar of bone crosses the right recess close to its medial margin. This bar does not appear to be fused with the margins of the recess, and is thus plausibly part of an underlying bone (the prootic?) emerging through the recess because of crushing. Therefore, the paracondylar recesses of both *Seazzadactylus venieri* and *Carniadactylus rosenfeldi* probably contained an undivided metotic foramen.

The basisphenoid (probably a parabasisphenoid as in most reptiles) and its basipterygoid processes are flattened in the same vertical plane as the occipital condyle and the foramen magnum, but were originally directed ventrorostrally (Codorniú et al., 2016, fig. 1a). As in *Dorygnathus banthensis* (see Padian, 2008a, figs. 12 and 17), *Bellobrunnus rothgaengeri* (see Hone et al., 2012, fig. 4) and probably *Carniadactylus rosenfeldi* (Dalla Vecchia, 2009a, fig. 2A) as well, the basisphenoid is subrectangular, nearly as broad as long, and with basipterygoid processes projecting at its lateroventral corners. The proximal part of the basisphenoid near the distal rim of the basioccipital is concave as in *Carniadactylus rosenfeldi* (see Dalla Vecchia, 2009a, fig. 2A). This concavity corresponds to the basioccipital–basisphenoid fossa of Gower & Sennikov (1996). The basipterygoid processes of the basisphenoid are long, rod-like, and slightly splayed laterally as in other non-monofenestratan pterosaurs (e.g. *Carniadactylus rosenfeldi*, Dalla Vecchia, 2009a, fig. 2A; 2014, fig. 4.1.103; *Raeticodactylus filisurensis*, Dalla Vecchia, 2014, fig. 4.1.160; *Dorygnathus banthensis*, Padian, 2008a, pl. 5/fig. 3, pl. 8/fig. 2, figs. 12 and fig. 17; and Fig. 9D; *Rhamphorhynchus muensteri*, Wellnhofer, 1975a, fig. 3d). Although unreported by Wellnhofer (2003) and Kellner (2015), the holotype of *Austriadraco dallavecchiai* also has a partially preserved occiput (Fig. 9C) and basipterygoid processes of the basisphenoid that are rod-like, elongated and slightly splayed laterally. This specimen does not show any trace of the cultriform process of the parasphenoid (reported also as “parasphenoidal rostrum”; Romer, 1956, p. 87) like that observed in *Dorygnathus banthensis* (see Padian, 2008a, fig. 12, but apparently absent in Fig. 9D), *Rhamphorhynchus muensteri* (see Wellnhofer, 1975a, fig. 3d), *Scaphognathus crassirostris* (see Wellnhofer, 1975b, fig. 35), *Cacibupteryx caribensis* (see Gasparini, Fernandez & De La Fuente, 2004, fig. 2D) and *Bellobrunnus rothgaengeri* (see Hone et al., 2012, fig. 4). This feature cannot be checked in *Seazzadactylus venieri* because the basisphenoid is covered distally by the left quadrate; this is also the case in *Carniadactylus rosenfeldi* where most of the basisphenoid is overlapped by a cervical vertebra and the parasphenoid rostrum—if present—is concealed by the right mandibular ramus (Dalla Vecchia, 2009a, fig. 2A). Maybe the cultriform process was not fused to the braincase in the holotype of *Austriadraco dallavecchiai* and displaced. Alternatively, it might have been broken or unossified.

Some elements occurring in the skull region close to the left wing phalanx 2 (Fig. 3) remain indeterminate, but they may belong to the braincase due to their size, morphology and position.

### Mandible

**Figure 10:**
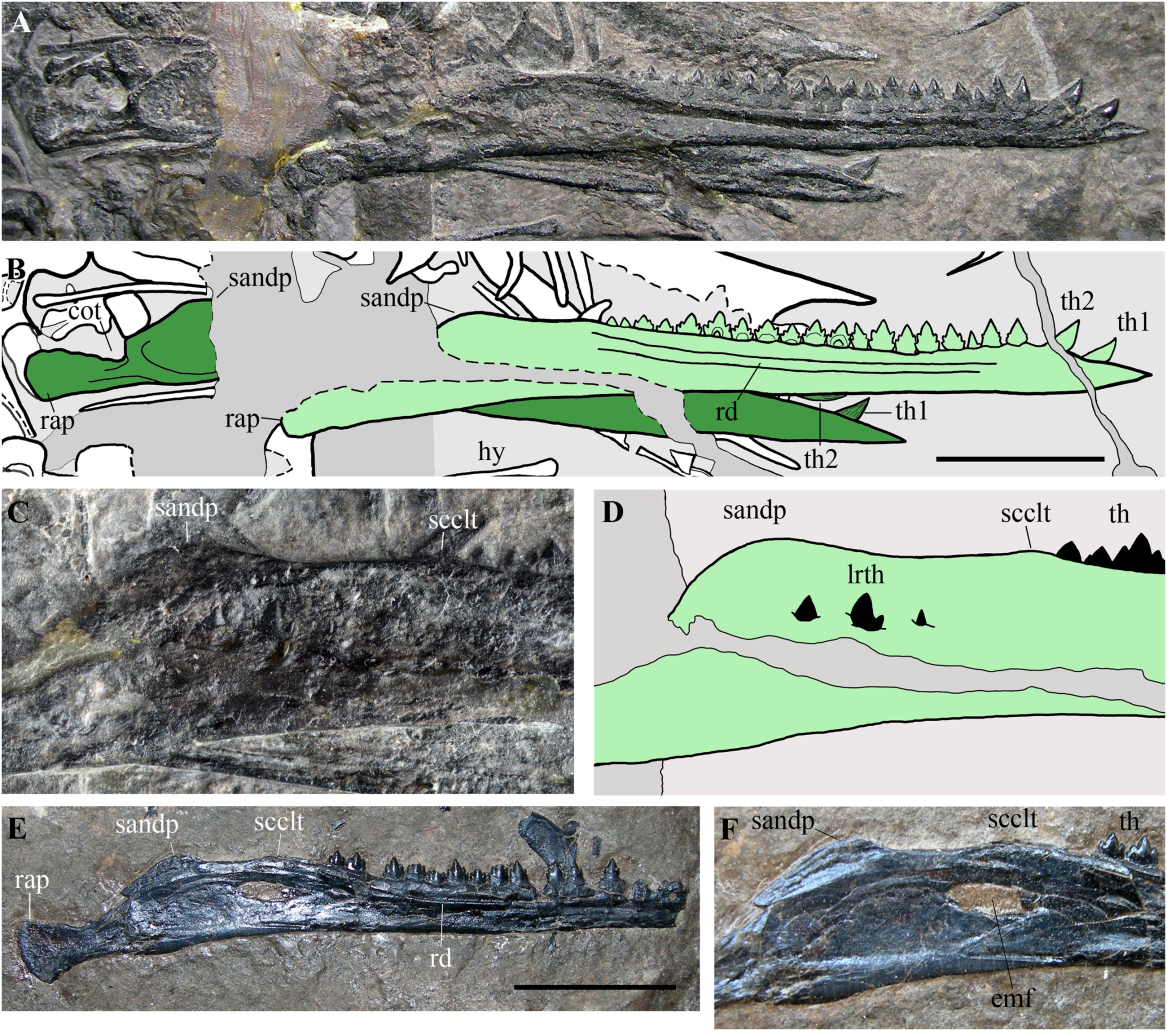
*Seazzadactylus venieri*, MFSN 21545 (holotype), mandible and comparison. (A) Mandibular rami of MFSN 21545; (B) drawing of (A) (the right ramus is pale green, whereas the left is dark green; black dashed lines mark the broken margins of the bones where they can be identified as such); (C) particular of the region caudal to the last tooth in the right ramus of MFSN 21545; (D) drawing of (C); (E) right mandibular ramus of *Austriadraco dallavecchiai*, holotype (BSP 1994 I 51); (F) particular of the region posterior to the last tooth in BSP 1994 I 51. Abbreviations: cot, cotyle; emf, external mandibular fenestra; hy, ceratobranchial I (hyoid apparatus); lrth, teeth of the left mandibular ramus; rap, retroarticular process; rd, ridge; sandp, dorsal process of the surangular; scclt, small convexity caudal to the last mandibular tooth; th, teeth; th1–2, first and second mandibular teeth. Scale bar equals 10 mm.

The two mandibular rami are associated with the skull and lie parallel to one other (Figs. 10A and 10B). The left ramus was shifted caudally with respect to the right ramus. The right ramus shows the lateral side and partly covers the left ramus in the middle. The left ramus is partly damaged by a fracture. The mandibular ramus is slender with a length/height ratio at mid ramus of 17.8 (length is 53.5 mm and height is only three mm).

Its rostral end is straight and sharply pointed, and the dentaries are not fused at the symphysis, which was probably very short. The dorsal margin of the ramus is shallowly concave in lateral view, while the ventral margin is straight. Height is constant along most of dentary, but the ramus slightly flares by mandibular tooth 4 and tapers rostrally to tooth 2. An arched longitudinal ridge, which is bordered by narrow ventral and dorsal grooves, runs along the lateral side of the dentary from tooth 4 to the last tooth. There is no external mandibular fenestra. Just caudal to the position of the external mandibular fenestra in *Austriadraco dallavecchiai* (Figs. 10C–10F), some teeth of the underlying left mandibular ramus pierced the wall of the right ramus and are exposed. This suggests that the wall was very thin in that area and could be easily broken, as in the case of *Dimorphodon macronyx* (see Bennett, 2015) and *Caelestiventus hanseni* (see Britt et al., 2018).

The dorsal margin of the ramus between the last tooth and the glenoid for the quadrate (Figs. 10C and 10D) shows the ‘two-peaked’ shape reported by Dalla Vecchia (2009a, p. 182; see also 2014, p. 82) as a peculiarity of *Austriadraco dallavecchiai* (Figs. 10E and 10F). The dorsal margin of the ramus has a small convexity just caudal to the last tooth which is followed by a straight segment (shallowly concave in the case of *Austriadraco dallavecchiai*) and then by a rounded process (the dorsal process of the surangular or ‘coronoid’ process). The latter is fractured at its base by crushing, which shows that it is a mediolaterally thin prominence. The retroarticular process is long and its caudal end is dorsoventrally expanded, lateromedially flattened and possesses a rounded profile in lateral view. It is slightly ventrally deflected, making with the dentary axis an angle of only 10–12°.

### Hyoid apparatus

Rod-like bones that lie parallel to one other and to the mandibular rami are the ossified ceratobranchials I of the hyoid apparatus. One lies ventral to the mandibular rami in its natural position, whereas the other is slightly displaced dorsocaudally and lies near the caudal part of the left mandibular ramus (Fig. 3). They are nearly straight and slightly expanded at their extremities like those of *Carniadactylus rosenfeldi* (see Dalla Vecchia, 2009a, fig. 2).

### Dentition

The dentition of this specimen is the most completely preserved among known Triassic pterosaurs with multicusped teeth except for that of the holotype of *E. ranzii* (see Dalla Vecchia, 2014). It is composed of four premaxillary, 14 maxillary and 21 mandibular teeth per side.

**Premaxillary teeth.** Four premaxillary teeth are outside their alveoli but close to the rostral tip of the premaxillae. Two right alveoli can be identified, but only one—the last and presumably that of tooth 4—is clearly visible (Fig. 4), whereas the first two alveoli are covered by a shed tooth. The shed teeth may be the right teeth 1–4. The first left tooth, still in situ at the apex of the rostrum, points forwards and its crown is slightly recurved rostroventrally. It is followed distally by another left tooth still in its alveolus, but pushed inside the premaxilla by crushing and appearing as a small mound on the dorsal surface of the bone (Fig. 4); since it occurs at the same distance from the tip of the snout as the last right alveolus, it is probably the left tooth 4.

**Figure 11:**
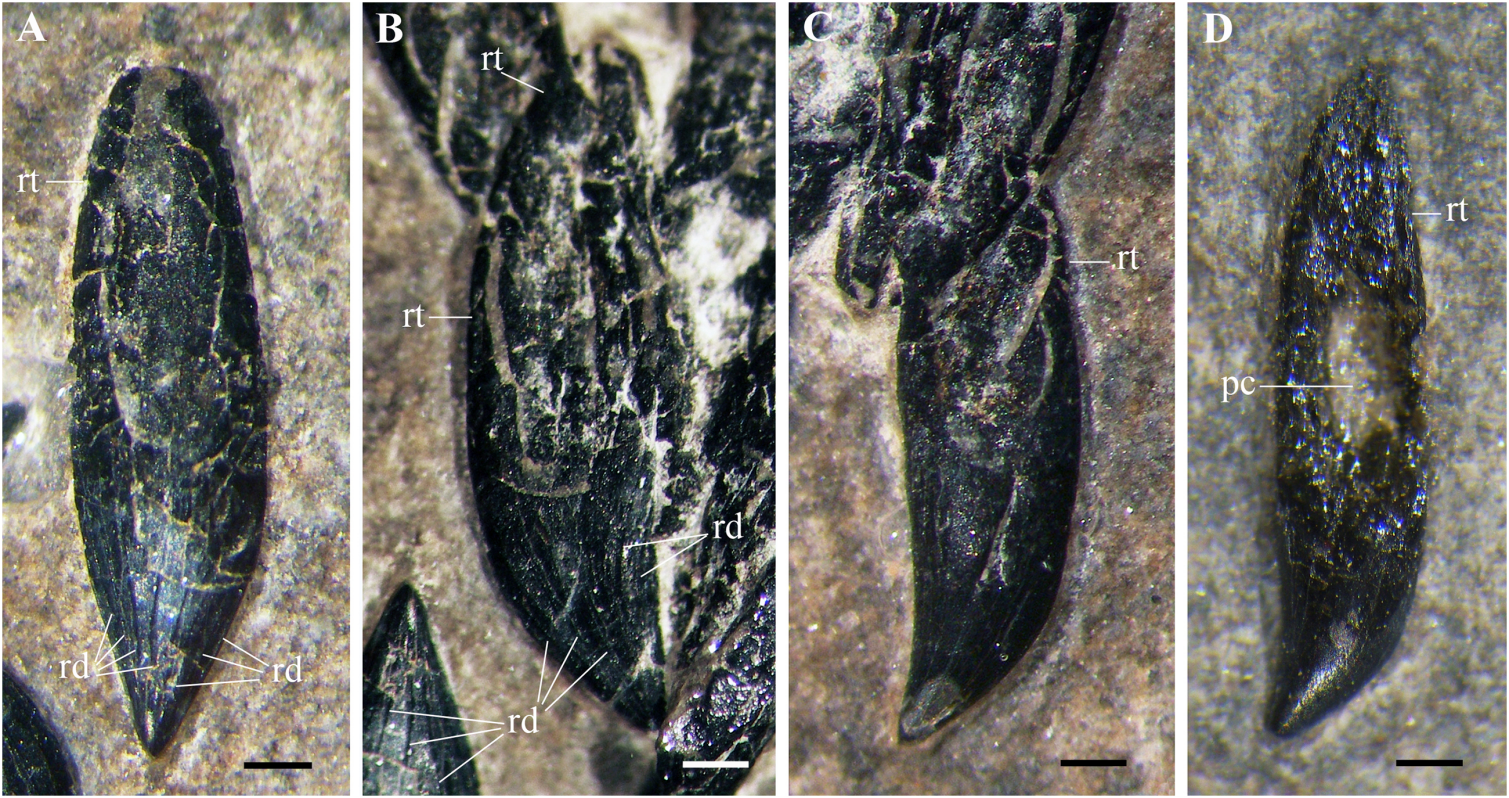
*Seazzadactylus venieri*, MFSN 21545 (holotype), premaxillary teeth. (A–B) teeth in lingual (A) and linguodistal (B) view; (C–D) teeth in labial view (if they are all right teeth). Photographs in (A–C) were taken under ethanol immersion. Abbreviations: pc, pulp cavity; rd, apicobasal ridges; rt, ‘root’. Scale bar equals 0.3 mm.

The crowns of the shed teeth are similar in shape and size to those of the symphysial mandibular teeth, but they are slightly more slender. They are unicuspid, conical and recurved. The crown of left tooth 1 is slightly flattened labiolingually and is recurved with the concave side facing ventrodistally. The other teeth are shed; thus, their orientation must be deduced by comparison. Thin, straight and spaced apicobasal enamel ridges are present only on one side, whereas the rest of the surface is smooth (compare Figs. 11A, 11B and 11C, 11D). Crown curvature is seen in teeth showing the smooth side. The labial side of the first two unicuspid mandibular teeth is smooth, whereas the lingual side has apicobasal enamel ridges. In the unicuspid mandibular teeth 1–3 of *Raeticodactylus filisurensis*, the enamel wrinkles occur only on the lingual side (Stecher, 2008). This suggests that the side with basoapical enamel ridges of the premaxillary teeth of *Seazzadactylus venieri* is the lingual one; consequently, crowns of Figs. 11A and 11B are lingually and linguodistally recurved, respectively, while those of Figs. 11C and 11D are distally recurved (if they are all from the right premaxilla). The total basoapical length of the teeth is 4.2–4.5 mm. The ‘root’ is only slightly longer than the crown and there is no constriction between crown and ‘root’. One tooth (Fig. 11D) has an exposed pulp cavity because the side of the tooth was damaged or it was reabsorbed by a growing replacement tooth.

**Maxillary teeth.** Maxillary crowns are exposed in labial view in both maxillae. All crowns have smooth surfaces. Teeth 8, 10, 14 and possibly tooth 1 on the right maxilla and teeth 6 and 14 on the left maxilla are not fully erupted. The positions of the left teeth 8 and 13 are represented by empty alveoli, but the displaced tooth 8 is preserved close by its alveolus. Crowns 3, 5 and 7 are 1.75 mm high and crown 9 is 1.60 mm high; the penultimate right crown is one mm high like the left crown 12. Maxillary tooth crowns are slightly larger than mandibular crowns (like *Raeticodactylus filisurensis*; Dalla Vecchia, 2014, fig. 4.1.161C); this size difference is more marked in the mesial half of the maxillary dentition (see Fig. S5). In the right maxilla, crowns 1–7 are basoapically higher than mesiodistally long, crown 9 is as high as long and the last three crowns are much longer than high. In the left maxilla, crown 2 is basoapically higher than mesiodistally long, crowns 5 and 8 are slightly apicobasally higher than mesiodistally long, whereas crowns 9–12 are longer than high. The first three crowns are slightly procumbent and slightly recurved backwards with curvature decreasing from tooth 1 to 3, whereas the following crowns are upright and straight. Crowns are not contacting one other, but the mesiodistal spacing between mid-maxilla fully erupted teeth is less than half the mesiodistal length of a fully erupted crown.

**Figure 12:**
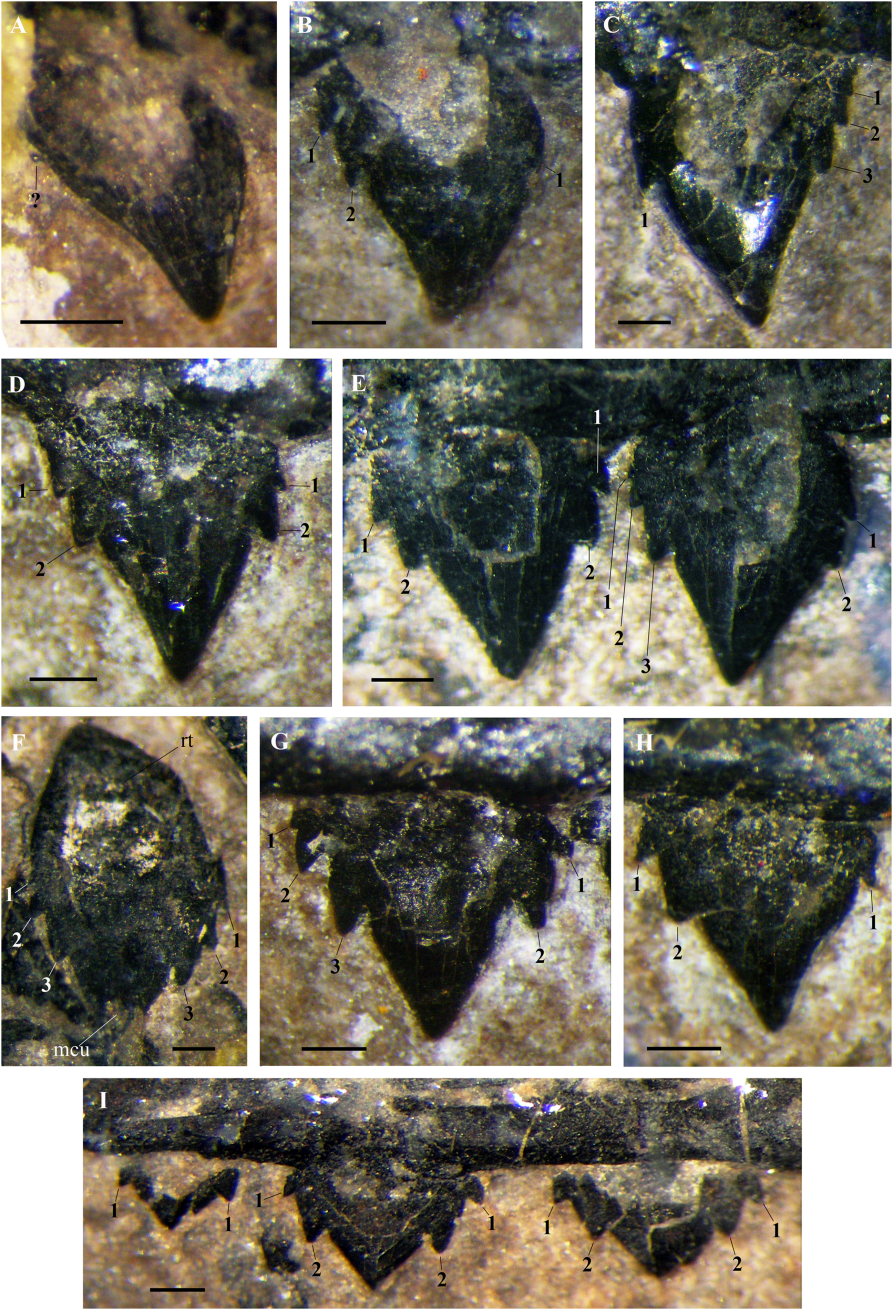
*Seazzadactylus venieri*, MFSN 21545 (holotype), maxillary teeth. (A) Right crown 1; (B) right crown 2; (C) left crown 2; (D) right crown 3; (E) right crowns 4–5; (F) left tooth 8 (displaced); (G) right crown 9; (H) right crown 11; and (I) right crowns 12–14. Photographs were taken under ethanol immersion. Abbreviations: 1–3, accessory cusps along each cutting margin, mcu, main cusp; rt, ‘root’. Scale bar equals 0.3 mm.

With the possible exception of the first tooth (Fig. 12A), crowns are multicusped (Figs. 12B–12I). The main cusp is triangular in labial view and moderately flattened labiolingually. The first three maxillary crowns differ slightly from the first two or three multicusped mandibular teeth, whereas crowns distal to maxillary tooth 3 have a similar shape as the mandibular crowns distal to tooth 4 or 5.

**Figure 13:**
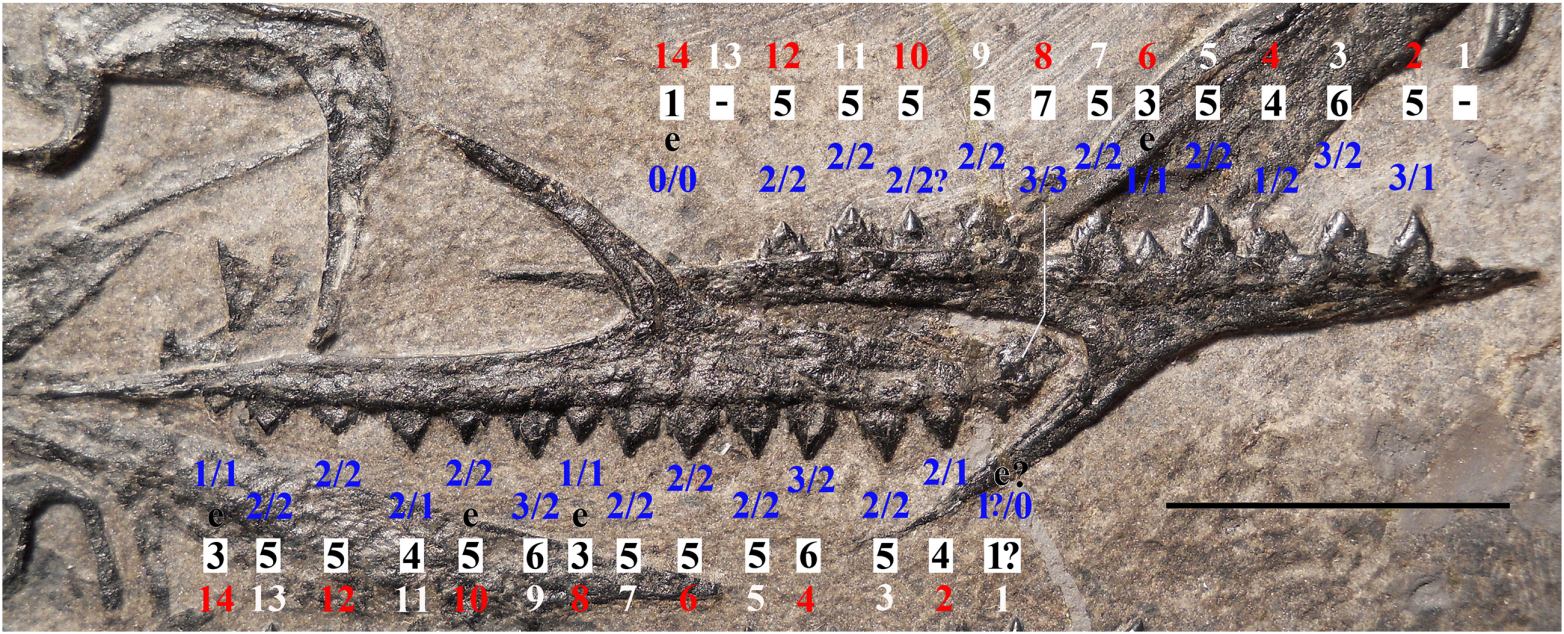
*Seazzadactylus venieri*, MFSN 21545 (holotype), cuspidation pattern of the maxillary teeth. The outer row of numbers (alternating white and red numbers) refers to the tooth position, the middle row (black numbers) is the total cusp number per tooth and the inner row (blue numbers) contains the number of accessory cusps on the mesial (right) and distal (left) cutting margin of each crown. The left maxilla is upside-down. Teeth are described in SI2. Abbreviations: e, erupting tooth that shows only part of the crown. Scale bar equals 10 mm.

In the left maxilla tooth 1 is missing. The crown of the right tooth 1 (Fig. 12A) has an inflated basal part and a distally recurved apical part. It is smaller than the following teeth and possibly not fully erupted. A very small accessory cusp might be present distally, but the crown appears to be basically unicuspid and resembles the premaxillary crowns. The cuspidation pattern of the following teeth is summarised in Fig. 13. Crowns are mainly pentacuspid with two mesial and two distal accessory cusps (Figs. 12D, 12E and 12I), but there is also a pentacuspid crown with three distal and one mesial accessory cusps (Fig. 12C), a heptacuspid crown with three mesial and three distal accessory cusps (Fig. 12E), three hexacuspid crowns with two mesial and three distal accessory cusps (Fig. 12G) and two tetracuspid crowns with one mesial and two distal accessory cusps (Figs. 12B and 12H). There are no fully erupted tricuspid teeth. Accessory cusps increase in size from the basal to the apical one. The cuspidation pattern differs in corresponding teeth of the left and right maxillae (Fig. 13), as it was observed in *E. ranzii* (see Wild, 1979).

The basal part of the crown has a more or less developed pit in all teeth, which could be due to basal resorption by the growing replacement tooth, as in some mandibular crowns (see below), but it was most probably caused by the collapse of its pulp cavity.

The ‘root’ is visible only in the displaced left tooth 8: it is tongue-shaped and as deep as the crown is high.

Details of the individual teeth are reported in SI2.

**Figure 14:**
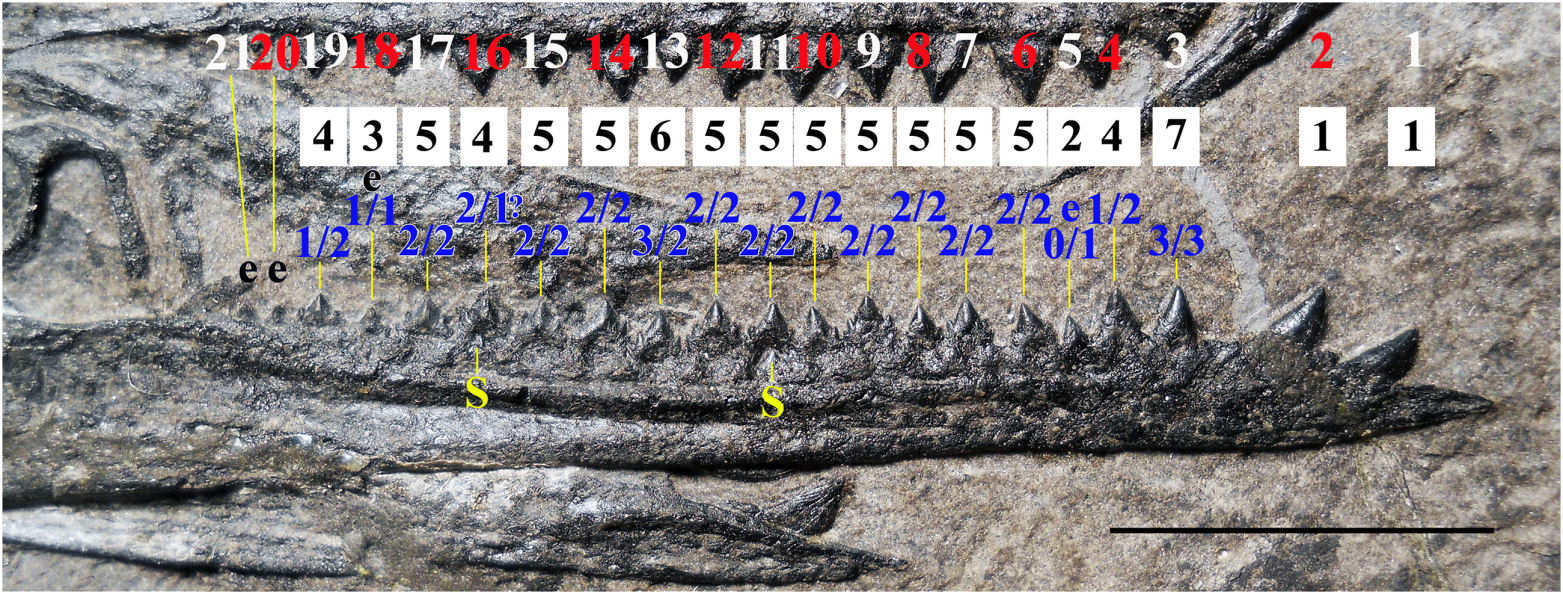
*Seazzadactylus venieri*, MFSN 21545 (holotype), cuspidation pattern of the mandibular teeth. Cuspidation pattern in the right mandibular ramus. The upper row of numbers (alternate white and red numbers) refers to the tooth position, the middle row (black numbers) is the total cusp number per tooth and the lower row (blue numbers) contains the number of accessory cusps in mesial (right) and distal (left) cutting margins of each crown. Teeth are described in SI2. Abbreviations: e, erupting tooth that shows only part of the crown; S, replacement tooth. Scale bar equals 10 mm.

**Mandibular teeth.** The right mandibular ramus exposes its entire dentition (21 teeth) in labial view (Fig. 14). Teeth 5, 18, 20 and 21 are not fully erupted. The ratio of tooth number/mandible length is 0.39. The dentition of the left mandibular ramus, exposed in lingual view, is mostly covered by the right ramus. The first left mandibular tooth is in situ whereas the second is out of its alveolus but close by. Crushing and probably preparation caused three mid-distal left mandibular crowns (approximately corresponding to teeth 13–15) to crop out through the right ramus and be partially visible (Figs. 10C and 10D).

**Figure 15:**
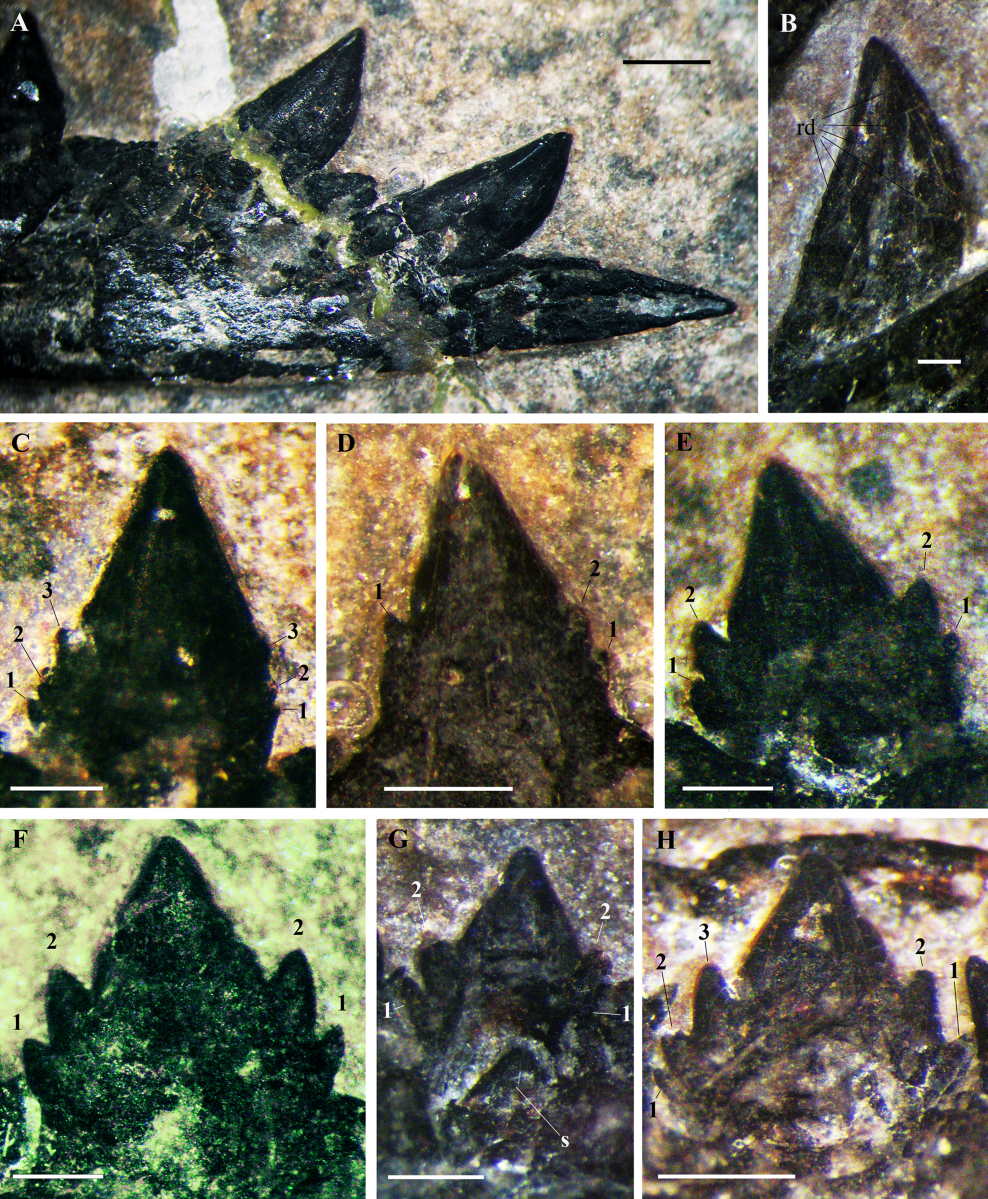
*Seazzadactylus venieri*, MFSN 21545, mandibular teeth. (A) Right teeth 1 and 2, labial view; (B) left crown 1, lingual side with thin apicobasal ridges; (C) right crown 3; (D) right crown 4; (E) right crown 6; (F) right crown 9; (G) right tooth 11 with the replacement tooth; and (H) right crown 13. Photographs were taken under ethanol immersion. Abbreviations: 1–3, accessory cusps along each cutting margin; rd, basoapical ridges; s, replacement tooth. Scale bar equals one mm in (A) and 0.3 mm in (B–H).

The first two mandibular teeth (Figs. 15A and 15B) have unicuspid, conical and pointed crowns that are relatively stout and slightly recurved backwards. These crowns are slightly bulkier than the premaxillary crowns. They are procumbent; the first more than the second. These crowns are not much larger than those of fully grown mid-mesial multicusped mandibular teeth (they are ca. 2.6 and 2.2 mm basoapically high, respectively, whereas crown 12 is ∼1.5 mm high). The lingual side of the crown (visible in the left teeth) has thin, straight and spaced basoapical enamel ridges, whereas the labial side is smooth.

A 1.3 mm-long gap separates crown 2 from crown 3. All crowns from crown 3 to 21 are multicusped, with one main central cusp and 1–3 accessory cusps along each mesial and distal margin (Figs. 14 and 15C–15H). All multicusped crowns have smooth surfaces. Crowns are conical and slightly labiolingually compressed, with an upright main cusp and basally-positioned accessory cusps. Cuspidation pattern is summarised in Fig. 14. Crowns are mainly pentacuspid with two mesial and two distal accessory cusps (Figs. 15E–15G), but tooth 3 has a heptacuspid crown with three mesial and three distal accessory cusps (Fig. 15C), tooth 13 has a hexacuspid crown with two mesial and three distal accessory cusps (Fig. 15H), teeth 4 and 19 have tetracuspid crowns with two mesial and one distal accessory cusps (Fig. 15D) and tooth 14 may have a tetracuspid crown with one mesial and two distal accessory cusps. The overall shape of crowns 3–4 is unlike that of the following crowns. Crowns 3–4 have small accessory cusps, whereas these cusps are larger in tooth 6 and following teeth and the apical accessory cusps are larger than the basal accessory cusps (Figs. 15C–15H). The main cusps are more flattened labiolingually in crown 6 onwards than in crowns 3–4. Crowns 3–4 are apicobasally much higher than mesiodistally long (Figs. 15C and 15D); crowns 6–7 are also apicobasally higher than mesiodistally long, but are comparatively longer mesiodistally than the preceding crowns (Fig. 15E); crowns 12 and 14 are nearly as mesiodistally wide as apicobasally tall and are the largest multicusped crowns in the mandible (height ∼1.5 mm). In the most distal teeth, crowns become mesiodistally longer than apicobasally high and with a slightly asymmetrical main cusp.

Spacing of the multicusped crowns is in general ca. 0.25 mm, that is, much less than half the mesiodistal length of the crown of a fully grown tooth; the splayed accessory cusps of adjacent teeth sometimes contact or even overlap.

Right teeth 11 and 16 (Fig. 15G) show the apical part of the replacement tooth growing inside the pulp cavity of the functional tooth because the functional crown is labially reabsorbed. Right crowns 10, 12–15 and 17 have a basal depression, possibly due to reabsorption by the replacement crown growing inside the basal part of the functional crown or because of the collapse of the pulp cavity.

### Axial skeleton

The vertebral column is disarticulated and its elements scattered. The caudal segment is totally missing.

**Cervical vertebrae.** Six cervical vertebrae can be identified based on their position, size and peculiar morphology (Dalla Vecchia & Cau, 2015). Part of the atlas is preserved on the left quadrate near the occipital region of the skull (Figs. 9A and 9B). It is craniocaudally short, kidney-shaped and with remnants of the pedicels, potentially representing the intercentrum of the atlas in craniocaudal view with part of the atlas neural arch (cf. Bennett, 2001). A cervical vertebra in left lateral view close to this bone (Fig. 3) is identified as the third cervical based on its position, size, outline of the neural spine and the well-developed prezygapophyses. The axis is mostly covered by the atlas intercentrum and by the cervical vertebra 3. The other three cervicals occur near the scapulocoracoids (Fig. 2); the most proximal of the three is exposed in left lateral view, while the other two are probably in ventral view and badly crushed.

**Figure 16:**
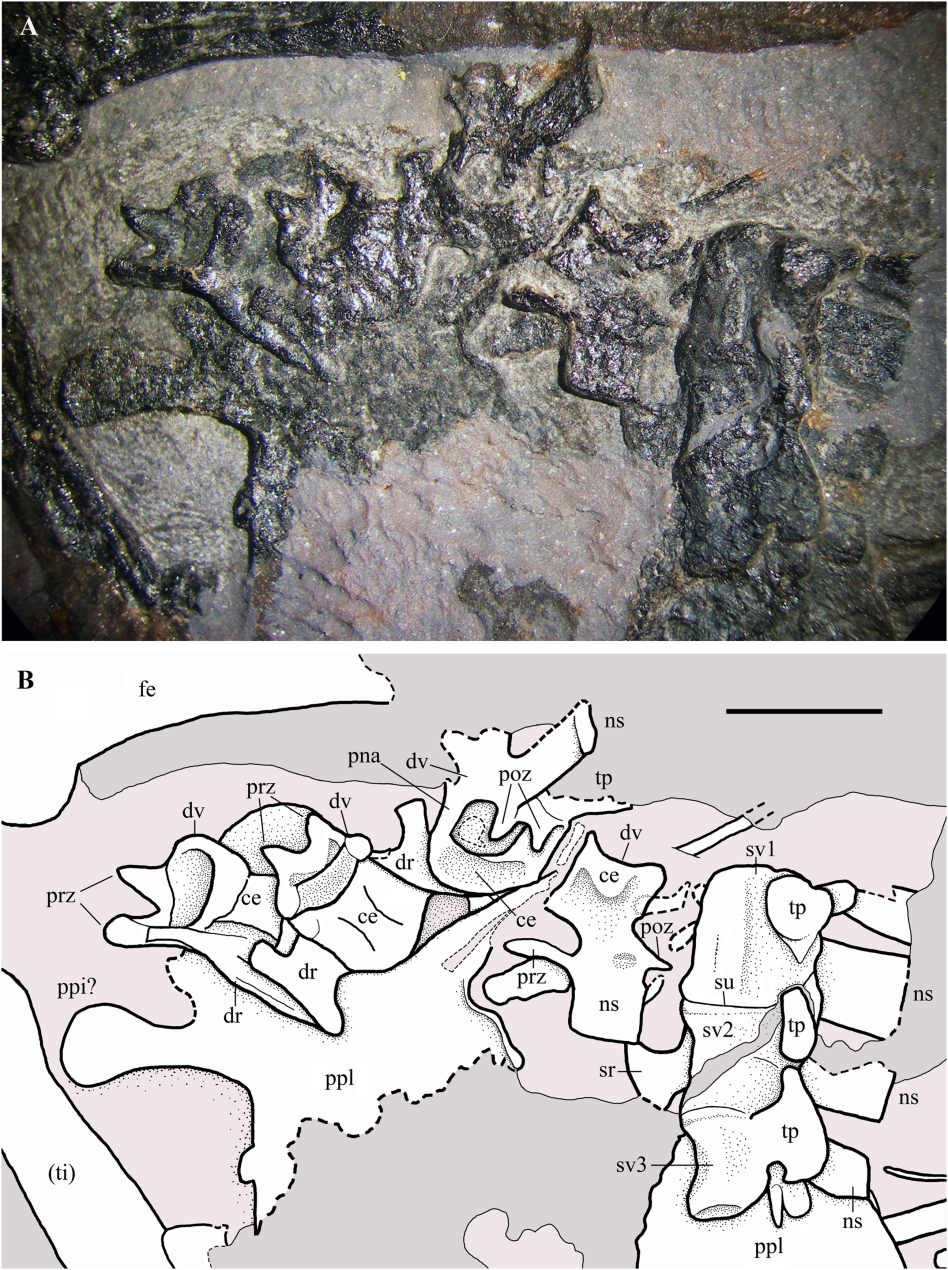
*Seazzadactylus venieri*, MFSN 21545 (holotype), last dorsal vertebrae and the sacrum. (A) Photograph; (B) drawing. Abbreviations: ce, vertebral centrum; dr, dorsal rib; dv, dorsal vertebra; fe, femur; ns, neural spine; pna, pedicel of the neural arch; poz, postzygapophysis; ppi, preacetabular process of ilium; ppl, pelvic plate; prz, prezygapophysis; sr, sacral rib; su, suture; sv1–3, sacral vertebrae 1–3; ti, tibiotarsus; tp, transverse process. Elements in parentheses are from the left side (when it was possible to distinguish between right and left elements). Scale bar equals five mm.

**Dorsal vertebrae.** Only seven out of the 14–16 dorsal vertebrae present in non-monofenestratan pterosaurs (Wellnhofer, 1975a; Wild, 1979; Padian, 2008a, 2008b; Bennett, 2014) can be reliably identified in the slab. They are gathered in two groups: one, proximal, is located between the scapulocoracoids (Fig. 2), whereas the other, distal, is close to the sacral vertebrae and the pelvis (Fig. 16). The many missing vertebrae are probably covered by other bones or were preserved in the portions of the slab that got lost. The better preserved dorsal vertebra of the first group is exposed in dorsal view and has a long and thin transverse process. It is as large as the cervicals and thus it is one of the anteriormost dorsals. Another vertebra that is close to the shaft of the right coracoid is much smaller. It is exposed in ventral view and also has a long transverse process directed caudolaterally; its centrum is cylindrical and unconstricted. The four dorsals of the second group are disarticulated, but close to one other. The last dorsal is exposed in right lateral view, the penultimate in caudal view and the other two in cranioventral view (Fig. 16). In lateral view, the centrum has a concave ventral margin. The cranial articular surfaces of the centra of the first two vertebrae of the second group are kidney-shaped (lower than wide) and concave; the caudal articular surface of the centrum of the penultimate dorsal also appears to be kidney-shaped and slightly concave. The postzygapophyses are smaller than the prezygapophyses. Although the last dorsal lacks transverse processes, these processes appear to be present in the penultimate dorsal. The last dorsal has a square neural spine that is slightly longer than high. The first two dorsals of the second group have associated ribs.

**Cervical and dorsal ribs.** Only shaft fragments and portions of the tubercula and capitula of the cervical and dorsal ribs are preserved. The ribs of the third to last dorsal vertebra are apparently dicephalous and have an unusually short shaft with a blunt distal end (Fig. 16B).

**Sacral vertebrae.** Three co-ossified sacral vertebrae are exposed in left lateral view near the last dorsal vertebra (Fig. 16). The faint suture between the centra of the sacrals 1 and 2 can be seen only under ethanol immersion. The neural spines are rectangular and that of the first sacral is taller than long. A fan-shaped sacral rib crops out from below the sacral vertebra 2. The sacrum is not co-ossified with the sacral ribs and the sacral ribs are not fused with the ilia.

**Figure 17:**
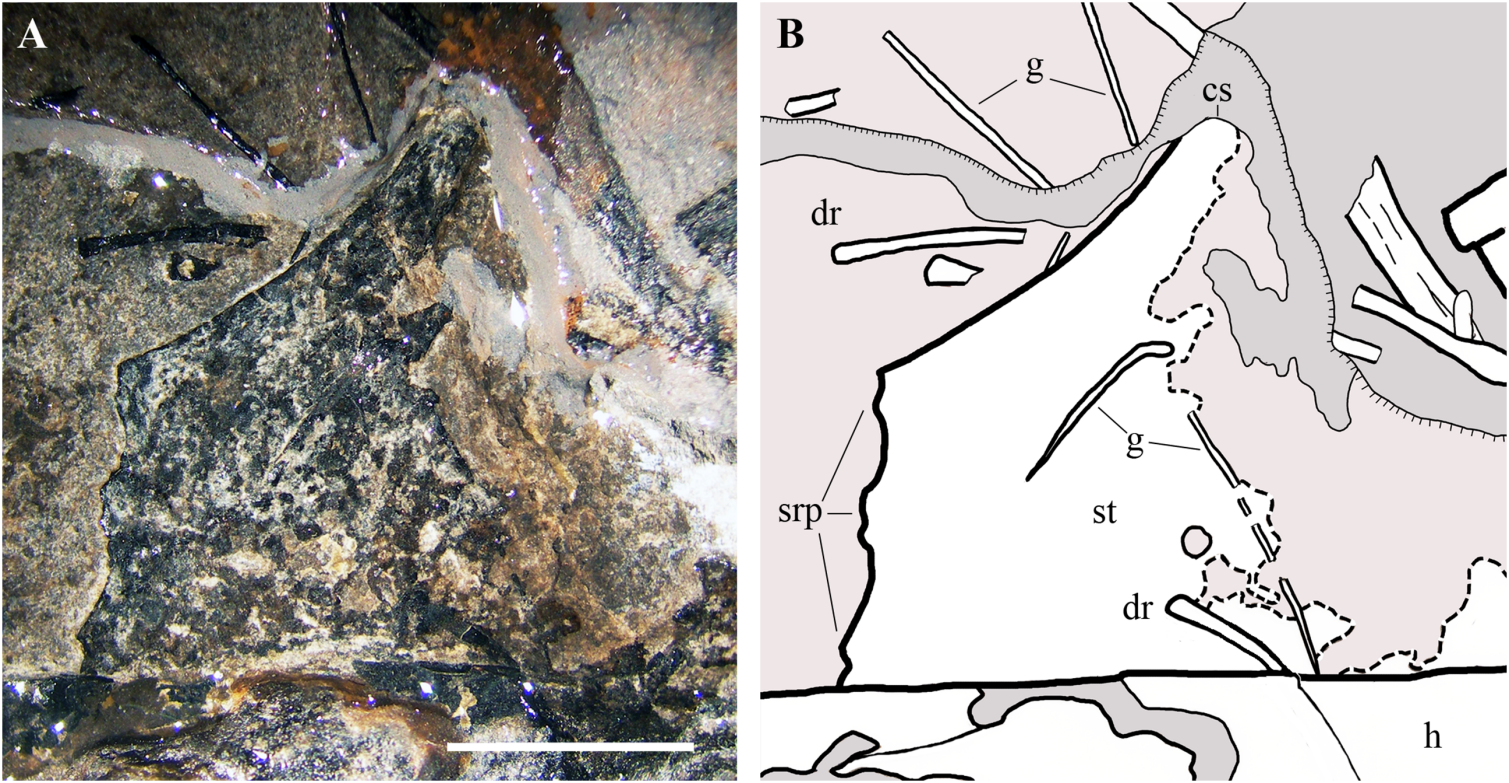
*Seazzadactylus venieri*, MFSN 21545 (holotype), sternum. (A) The preserved portion of the sternum (photograph taken under ethanol immersion); (B) drawing. Black dashed lines mark the broken margins of the bones where they can be identified as such. Abbreviations: cs, cristospine; dr, dorsal rib; g, gastrale; h, humerus; srp, processes for the sternal ribs; st, sternum. Scale bar equals five mm.

**Sternum.** Only the ?left half of the sternum is preserved (Fig. 17). It is a thin and broad plate with a triangular cranial portion and a square posterior part bearing three short lateral processes for the sternal ribs. Only the base of the cristospine is preserved and the caudal portion of the plate is concealed by the right humerus. This sternum resembles those of *E. ranzii* (see Wild, 1979, fig. 14) and MPUM 7039 (Dalla Vecchia, 2014, fig. 4.2.2A).

**Gastralia.** The gastralia are very thin bones that are straight or curved at one extremity and pointed at the other (Fig. 17). They are scattered around the sternum and the girdles.

### Pectoral girdle

**Figure 18:**
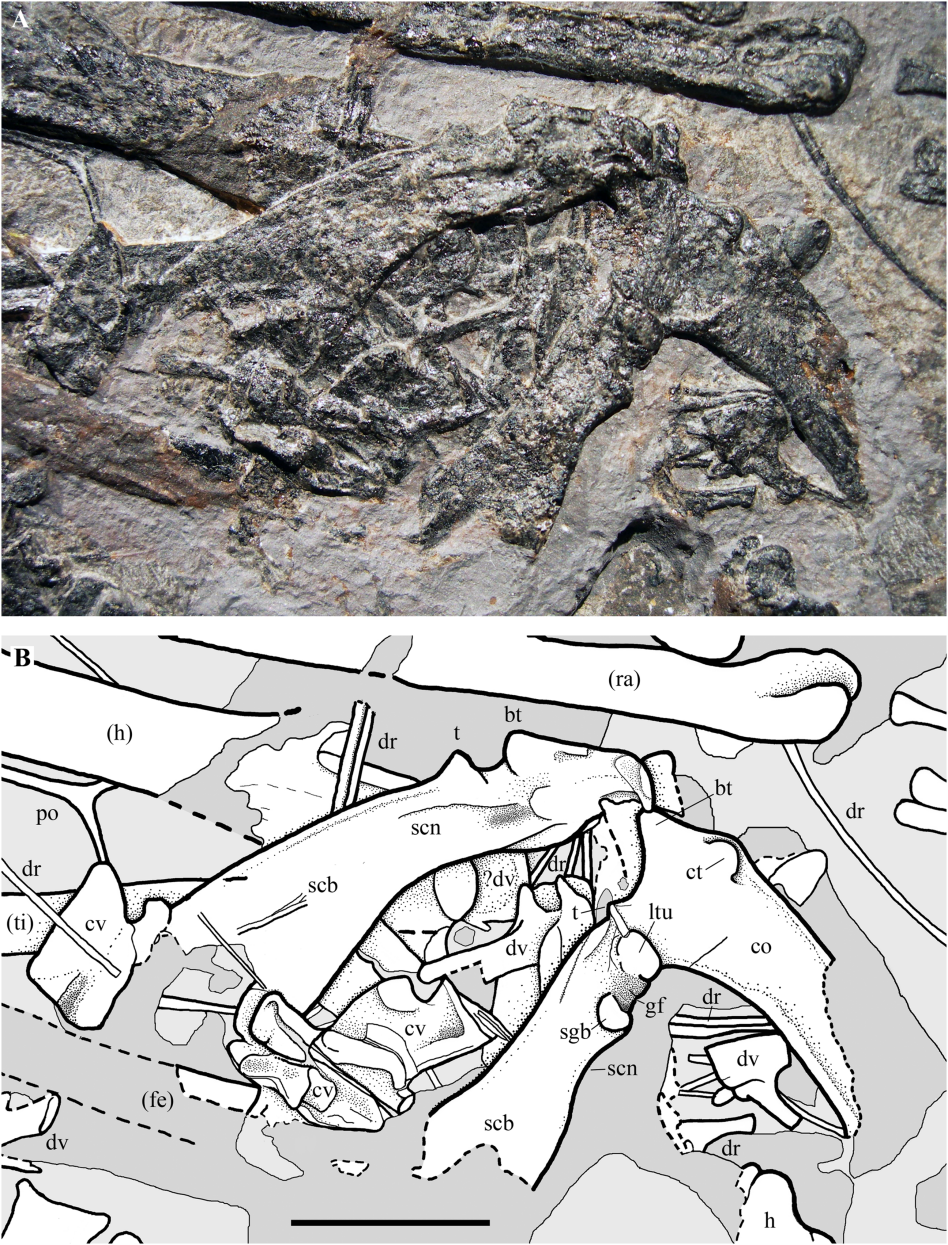
*Seazzadactylus venieri*, MFSN 21545 (holotype), scapulocoracoids. (A) Photograph; (B) drawing. Dashed lines mark the broken margins of the bones where they can be identified as such. Abbreviations: bt, biceps tubercle; co, coracoid; ct, coracoid tubercle; cv, cervical vertebra; dr, dorsal rib; dv, dorsal vertebra; fe, femur; gf, glenoid fossa; h, humerus; ltu, lower tuberosity; po, postorbital; ra, radius; scb, scapular blade; scn, scapular neck; sgb, supraglenoidal buttress, t, tubercle; ti, tibiotarsus. Elements in parentheses are from the left side (when it was possible to distinguish between right and left elements). Scale bar equals 10 mm.

The scapula and coracoid are fused. The right and left scapulocoracoids are exposed in lateral and medial view, respectively (Fig. 18). They are close and parallel to one other as is sometimes found to be the case in disarticulated pterosaur skeletons (e.g., Wild, 1979, pl. 8; Padian, 2008a, pl. 4/fig. 7).

The right coracoid lacks the distal portion of its shaft, and both scapulae also lack their distal portions. The shaft of the left coracoid is mostly missing; it is unclear whether this is due to the loss of fragments of the damaged slab and or to preparation and the imprecise fit of the slab fragments. The coracoid has a prominent biceps tubercle (sensu Bennett, 2003) at its dorsal extremity like the coracoids of *Carniadactylus rosenfeldi* and *Austriadraco dallavecchiai* and a coracoid tubercle (sensu Bennett, 2003) craniolaterally in the same position as in the coracoid of *Carniadactylus rosenfeldi* (Dalla Vecchia, 2009a; fig. 3). A small tubercle that is lower than the biceps tubercle occurs dorsal to the lower tuberosity (sensu Sangster, 2003) along the dorsal margin of the scapulocoracoid as in other Triassic pterosaurs (Dalla Vecchia, 2009a; fig. 3). This could be a remnant of the ‘acromion’ of the scapula after the fusion of scapula and coracoid. The glenoid is bordered by the lower tuberosity cranially and by the supraglenoidal buttress (sensu Sangster, 2003) caudally. The shaft of the right coracoid is flat and broad like those of *Carniadactylus rosenfeldi* and *Austriadraco dallavecchiai*, with parallel cranial and caudal margins (or craniomedial-caudolateral, according to its—unknown—articulation with the sternum). The angle between the right scapula and right coracoid is 76°. Distal to the glenoid the deep scapula exhibits a slight constriction (the scapular neck), beyond which the dorsal (dorsomedial, if crushing and flattening altered its original orientation) and ventral (ventrolateral) margins of the scapula diverge and the scapular blade flares markedly distally (Fig. 18).

### Forelimb

The right forelimb preserved in articulation from the humerus to the wing phalanx 1 (Figs. 1 and 2). Only the proximal part of the wing phalanx 2 is preserved, along the margin of the slab. The left forelimb is moderately disarticulated and lacks only the greater part of the wing phalanx 4. Both forelimbs are flexed at the elbow with the humerus and paired radius and ulna aligned parallel to one other.

**Humerus.** The right humerus (Fig. 2) is exposed in dorsal view. Part of the external tuberosity and the saddle-like articular margin are preserved, but the deltopectoral crest is missing and was reconstructed. The distal part of the shaft is recurved cranially; the distal articular end is missing and was reconstructed. The left humerus is represented only by most of its crushed shaft (Fig. 2).

**Figure 19:**
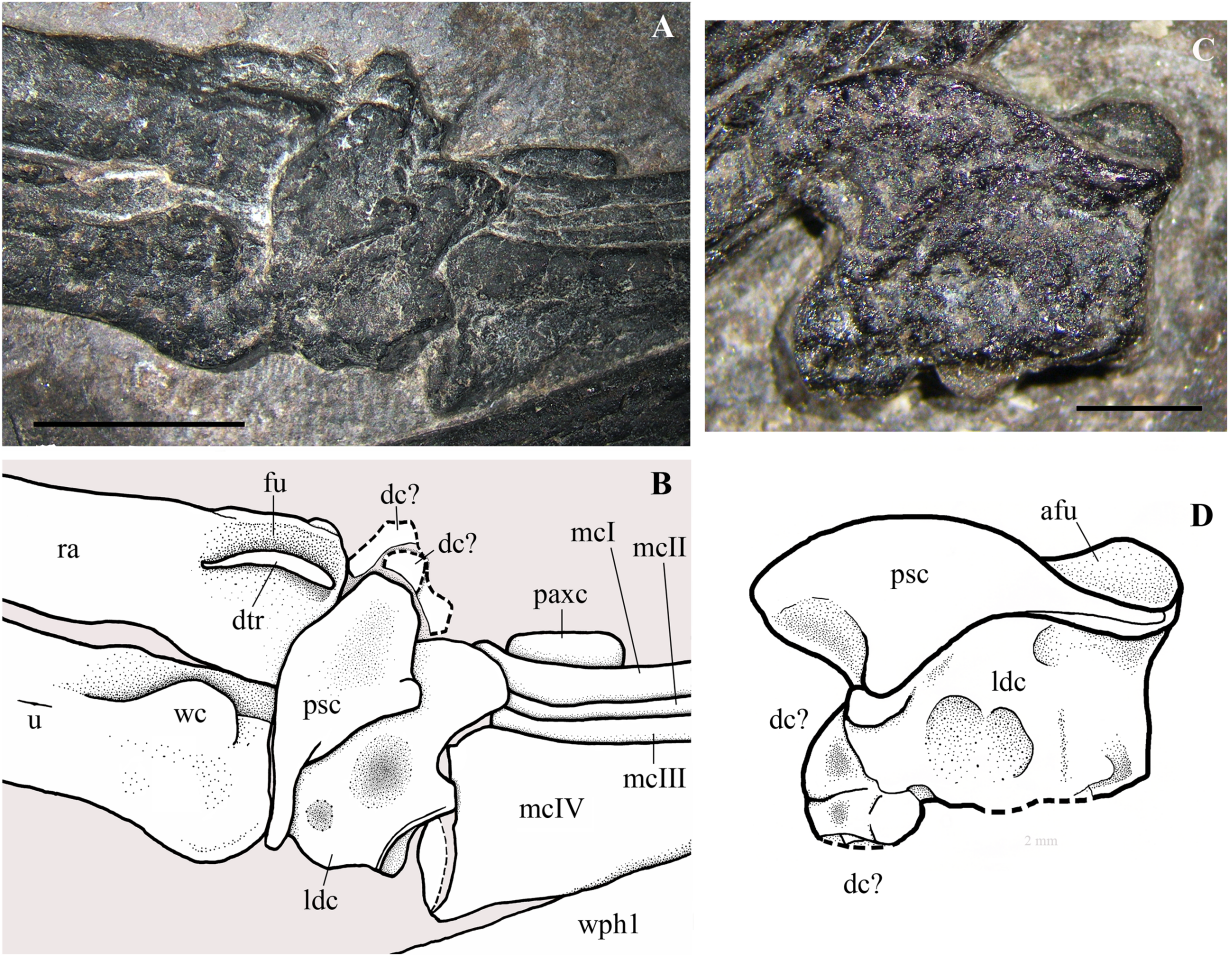
*Seazzadactylus venieri*, MFSN 21545 (holotype), carpus. (A) Right carpus, dorsal view; (B) drawing of (A); (C) left carpus, dorsal view; (D) drawing of (C). Dashed lines mark the broken margins of the bones where they can be identified as such. Abbreviations: afu, articular facet for ulna; dc, distal carpal; dtr, distal tubercle of radius; fu, furrow; ldc, large distal carpal; mcI–IV, metacarpals I–IV; paxc, preaxial carpal; psc, proximal syncarpal; ra, radius; u, ulna; wc, wing-like crest in the ulna; wph1, wing phalanx 1. Scale bar equals five mm in (A) and two mm in (B).

**Radius and ulna.** Radius and ulna are paired and lie parallel to one another in both forelimbs as is common in moderately disarticulated skeletons of early pterosaurs (e.g., Wellnhofer, 1975a, 1975b; Padian, 2008a, 2008b; Dalla Vecchia, 2014). The proximal portions of both radii and ulnae pairs are not preserved. The shafts of both elements are straight and have similar diameters. The right ulna (Figs. 19A and 19B) is exposed in dorsal view; its distal end bears a broad, flattened and wing-like crest like that on the upper surface of the distal part of the ulna in the holotype of *Carniadactylus rosenfeldi* (see Dalla Vecchia, 2014, fig. 4.1.113C1-2). The distal end of the right radius has a prominent longitudinal ridge bounded cranially by a broad furrow (Figs. 19A and 19B). The ridge could correspond to the distal tubercle of radii reported in other early pterosaurs (i.e. *Carniadactylus rosenfeldi* [see Dalla Vecchia, 2014, fig. 4.1.113C1-2] and *Dimorphodon macronyx* [see Sangster, 2003, fig. 3.7]); in which case the right radius is rotated so that its cranial side is partly exposed. The distal portions of the left radius and ulna differ noticeably in morphology from the distal ends of the right bones (Fig. S6). The distal termination of the left ulna, although partly reconstructed, is much more expanded than the distal termination of the radius. It closely resembles the distal end of the ulna of *Rhamphorhynchus muensteri* figured by Wellnhofer (1975a, fig. 12h) and comparison with it and with the associated radius indicates that the left ulna is exposed in cranial view (see also Bennett, 2001, fig. 76). It sends ventrally a flange like other pterosaurs (e.g. *Rhamphorhynchus muensteri*, Wellnhofer, 1975a, fig. 12h; *Dorygnathus banthensis*, Padian & Wild, 1992, pl. V, fig. 5). The moderately expanded distal end of the radius is divided into two condyle-like parts by a longitudinal furrow; comparison with the radius of *Rhamphorhynchus muensteri* figured by Wellnhofer (1975a, fig. 12g) and with the right radius suggests that the left radius is also in cranial view. The distal tubercle seems to be damaged.

**Carpus.** Both left and right carpi are exposed in dorsal view. The right carpus (Figs. 19A and 19B) is articulated with the radius-ulna and metacarpus. There is a single proximal syncarpal that is interlocked with a very large distal carpal. At least one, possibly two, other distinct and smaller elements crop out from below the cranial end of the proximal syncarpal, and do not contact the metacarpus. They are plausibly distal carpals that articulated with metacarpals I–III (Wellnhofer, 1975a, fig. 12a-b; Dalla Vecchia, 2009a, fig. 5A), which are slightly displaced and partially covered by the proximal syncarpal. They are damaged and their shape is therefore unclear.

A preaxial carpal with the same appearance as the right preaxial carpal of the holotype of *Carniadactylus rosenfeldi* and a similar position to it (see Dalla Vecchia, 2009a, fig. 5A) crops out cranially from the proximal part of metacarpal I.

The left carpus (Figs. 19C and 19D) is represented by a single carpal ‘block’ that is disarticulated and isolated but still very close to the radius-ulna and the wing metacarpal of the left wing (Figs. 1 and 2). The ‘block’ is made up of the interlocked proximal syncarpal and the large distal carpal. The cranial half of the left large distal carpal is apparently divided into at least two parts, but it is unclear whether this represents crushing of the irregularly shaped carpal or the existence of separate, distinct and smaller distal carpals. Comparison with the apparently homogeneous ‘nose’ of the right large distal carpal suggests that they are not distinct carpals. The carpal ‘block’ is rotated with respect to the left wing metacarpal and shows its dorsal side, as indicated by the position of the deep articular facet for the ulna in the proximal syncarpal.

The proximal syncarpals are saddle-like in dorsal view, thicker cranially (radial side), thinner caudally (ulnar side) and with a depressed ulnar facet. There is no suture between the radial and ulnar parts that compose each syncarpal. These elements resemble the proximal syncarpal of *Carniadactylus rosenfeldi* (see Dalla Vecchia, 2009a, fig. 5B), *E. ranzii* (see Wild, 1979, fig. 17) and *Rhamphorhynchus muensteri* (see Wellnhofer, 1975a, fig. 12b-f).

In dorsal view, the large distal carpals of MFSN 21545 are as craniocaudally broad as the proximal syncarpal and are proximodistally longer. The caudal half of each large distal carpal, which articulates with the wing metacarpal, is squared and massive, whereas the cranial half, which articulates with the metacarpals III, II and possibly I, is nose-like.

The actual shape of the preaxial carpal is unknown because it is partly concealed by the metacarpus (cf. Dalla Vecchia, 2009a, fig. 5).

**Figure 20:**
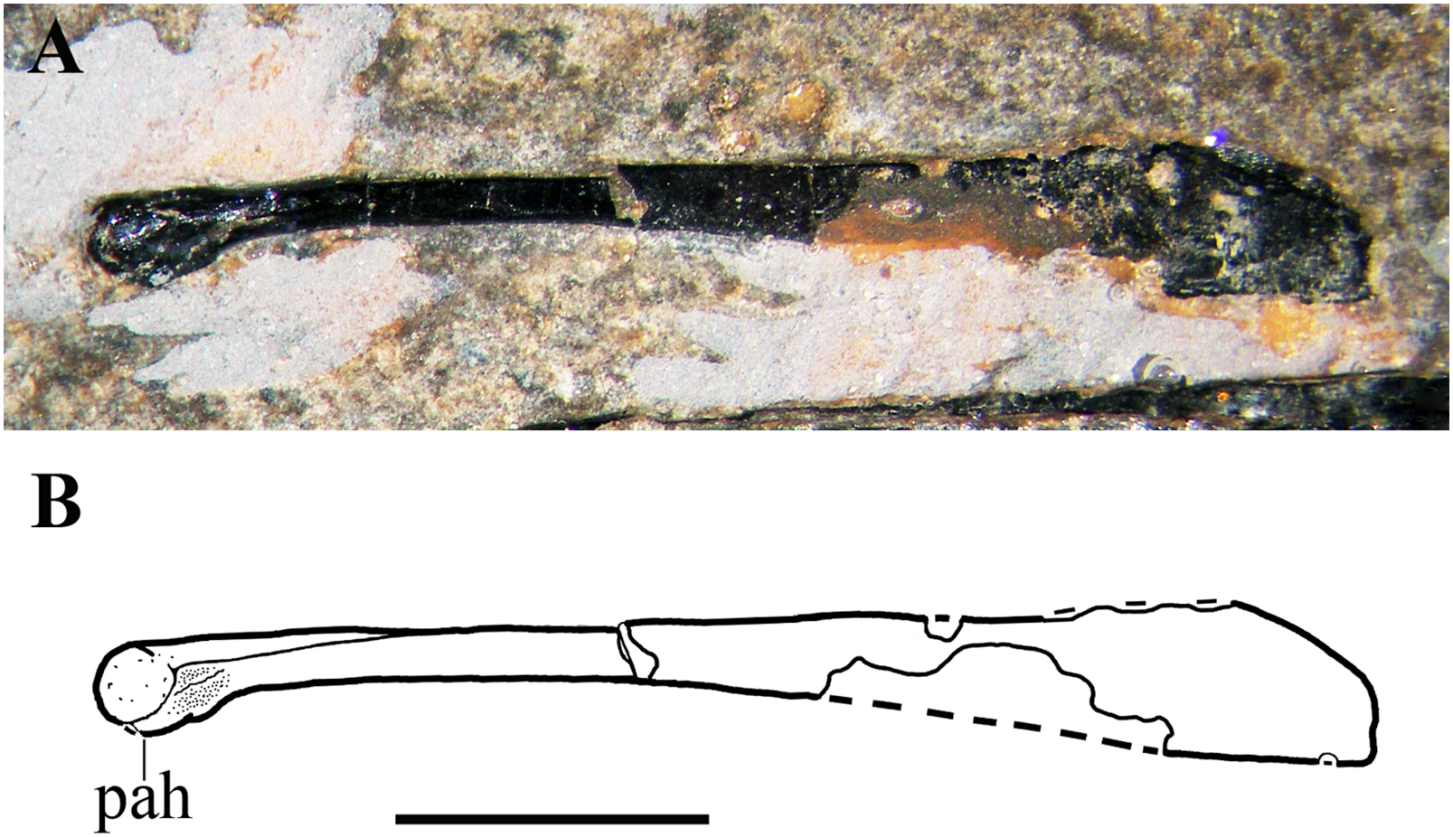
*Seazzadactylus venieri*, MFSN 21545 (holotype), pteroid. (A) Photograph taken under ethanol immersion; (B) drawing. Dashed lines mark the missing margins of the bone. Abbreviations: pah, proximal articular head. Scale bar equals three mm.

**Pteroid.** A pteroid (Fig. 20) is preserved close by and parallel to the proximal part of the right wing phalanx 1. It is therefore shifted away from its natural articulation with the proximal syncarpal. Although it is closest to the right manus, the left manus is also close and disarticulated. Thus, it cannot be established whether it is a right or left pteroid. If it is the right pteroid, its proximal portion is exposed in ventro-caudal view, whereas the distal part is exposed in caudal view, due to mid-shaft fracturing and the slight rotation of the distal part. The distal portion is partly damaged, but its shape can be reconstructed without ambiguity. The pteroid has the shape of an exclamation mark, with a craniocaudally flattened shaft that broadens and becomes thinner distally. The distal end is spatula-shaped, thin and flattened. The shaft is straight, but it is slightly bent caudally at its beginning just distal to the proximal articular head. The latter is slightly expanded and subspherical. This 12.3 mm long pteroid is comparatively short compared with those of other Triassic pterosaurs (Table S1).

**Figure 21:**
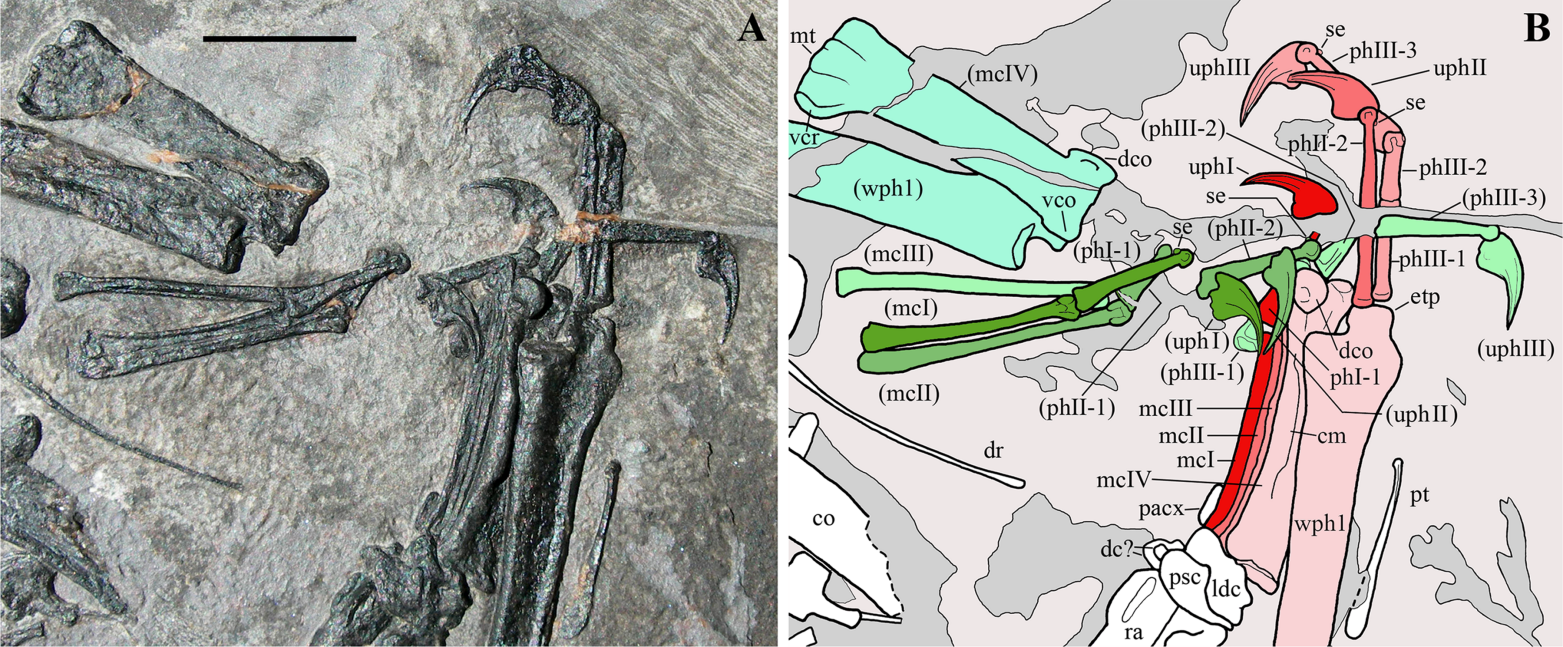
*Seazzadactylus venieri*, MFSN 21545 (holotype), mani. (A) Photograph; (B) drawing. Bones of the left manus are in green tones, whereas those of the right manus are in red-pink tones. Abbreviations: cm, crista metacarpi; co, coracoid; dc, distal carpal; dco, dorsal condyle of the wing metacarpal; dr, dorsal rib; etp, extensor tendon process of wing phalanx 1; ldc, large distal carpal; mcI–III, metacarpals I–III; mcIV, wing metacarpal; mt, medial tuberosity of the wing metacarpal; pacx, preaxial carpal; phI-1, phalanx 1 of digit I; phII-1 and 2, phalanges 1 and 2 of digit II; phIII-1, 2 and 3, phalanges 1, 2 and 3 of digit III; psc, proximal syncarpal; pt, pteroid; ra, radius; se, sesamoid; uphI–III, ungual phalanges I–III; vco, ventral condyle of the wing metacarpal; vcr, ventral crest of the wing metacarpal; wph1, wing phalanx 1. Bones in parentheses are from the left side (when it was possible to distinguish between right and left elements). Scale bar equals 10 mm.

**Metacarpus.** The right metacarpus is perfectly articulated (Fig. 21). The metacarpals lie parallel to one another and metacarpals I–III overlap proximally and partly longitudinally. The distal ends of metacarpals II and III are covered by the ungual phalanx of left digit II. The left metacarpus is slightly disarticulated. The left wing metacarpal is close to the left wing phalanx 1, whereas the left metacarpals I–III have shifted ventrally as a unit. Left metacarpals I and II remained articulated to one other, while metacarpal III is disarticulated. Left metacarpals I and II expose their cranial side, showing the distal ginglymi, which have a broad intercondylar sulcus. The proximal extremities of left metacarpals I and II are convex. The shaft of left metacarpal I has a broad longitudinal groove, probably caused by the collapse of the thin cortex. The left metacarpal III has an asymmetrically expanded proximal end. Left metacarpals II and III have the same length (18 mm), whereas metacarpal I is decidedly shorter (it is 78% the length of metacarpal II).

**Figure 22:**
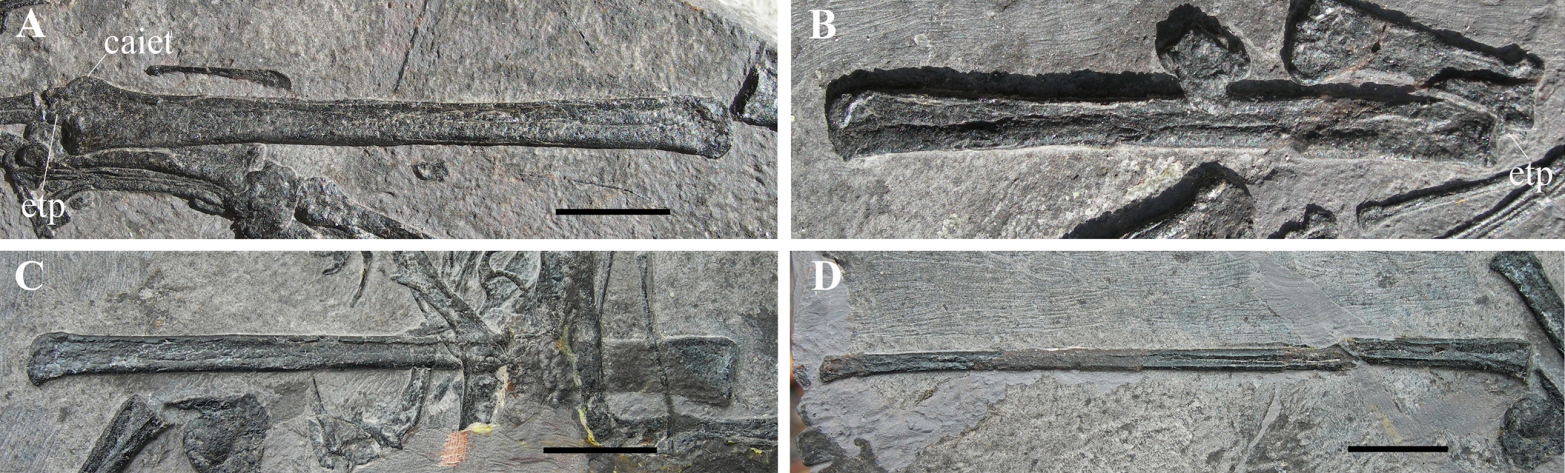
*Seazzadactylus venieri*, MFSN 21545 (holotype), wing phalanges. (A) Right wing phalanx 1, dorsal view; (B) left wing phalanx 1, dorsal view; (C) left wing phalanx 2, dorsal view; (D) left wing phalanx 3, dorsal view. Abbreviations: caiet, additional insertion of the extensor tendon of wing phalanx 1; etp, extensor tendon process of wing phalanx 1. Scale bar equals 10 mm.

The wing metacarpals (IV) are similar to the wing metacarpals of the other Triassic pterosaurs (Dalla Vecchia & Cau, 2015). They are much more robust than metacarpals I–III and slightly longer than metacarpals II–III. They have the same length as the wing metacarpal of the holotype of *Carniadactylus rosenfeldi* (21 mm). The left wing metacarpal is exposed in cranial view and shows a well-developed proximal ventral flange, a hint of the median tuberosity, the proximal depression for metacarpals I–III and the distal condylar end with a larger and slightly dorsally splayed dorsal condyle. The right wing metacarpal is exposed in caudodorsal view, showing a prominent *crista metacarpi* (Dalla Vecchia & Cau, 2015).

**Phalanges of manual digits I–III.** The left manus is disarticulated and the scattered phalanges of digits I–III are mixed together with those of the right manus, but left phalanges can be distinguished from right ones (Fig. 21). The phalangeal formula is 2-3-4-4-0. All non-ungual phalanges of digits I–III are straight. The penultimate (pre-ungual) phalanges are the longest (see Table S2); phalanx III-2 is the shortest phalanx in these digits (it is nearly half the length of phalanx III-3). In the penultimate phalanges, the shafts taper distally and the distal ginglymi are well-shaped, with a semicircular outline and lateral pits for the collateral ligaments. There is a small antungual sesamoid dorsally on all of these ginglymi. Similar sesamoids are reported in *E. ranzii*, *Carniadactylus rosenfeldi*, *Peteinosaurus zambellii* and MCSNB 8950 (Wild, 1979, 1994). The ungual phalanges are of similar sizes to one another (length range 6.2–7 mm, when not damaged). They are sharply pointed, moderately recurved and dorsoventrally flattened. They have a longitudinal groove for the attachment of the horny sheath and a large flexor tubercle. They resemble the pedal phalanges of the holotype of *Carniadactylus rosenfeldi* (see Dalla Vecchia, 2009a, fig. 8; 2014, fig. 4.1.128) and are only slightly larger than them (cf. Dalla Vecchia, 2009a, tab. 1). Unfortunately, no ungual phalanges of the pedes are preserved in MFSN 21545. Thus, it cannot be established whether *Seazzadactylus venieri* had manual unguals only slightly larger than pedal unguals (as comparison with the similarly-sized holotype of *Carniadactylus rosenfeldi* would suggest) or just smaller manual unguals with respect to *Carniadactylus rosenfeldi*. The rounded ginglymi of the penultimate phalanges allowed a high range of flexion and extension of the unguals.

**Wing phalanges.** Wing phalanx 3 is the longest and wing phalanx 1 the shortest (Table S1), but the length of wing phalanx 4 is unknown. As in other Triassic pterosaurs, the proximal part of wing phalanx 1 is enlarged and bear a robust extensor tendon process and a broad preaxial crest for additional insertion of the extensor tendon of the phalanx (cf. Wellnhofer, 1991, fig. 34). The extensor tendon process is fused without visible suture to the proximal part of the phalanx. Wing phalanges 1 and 2 are straight, whereas the distal end of wing phalanx 3 is slightly bent caudally.

### Pelvic girdle

**Figure 23:**
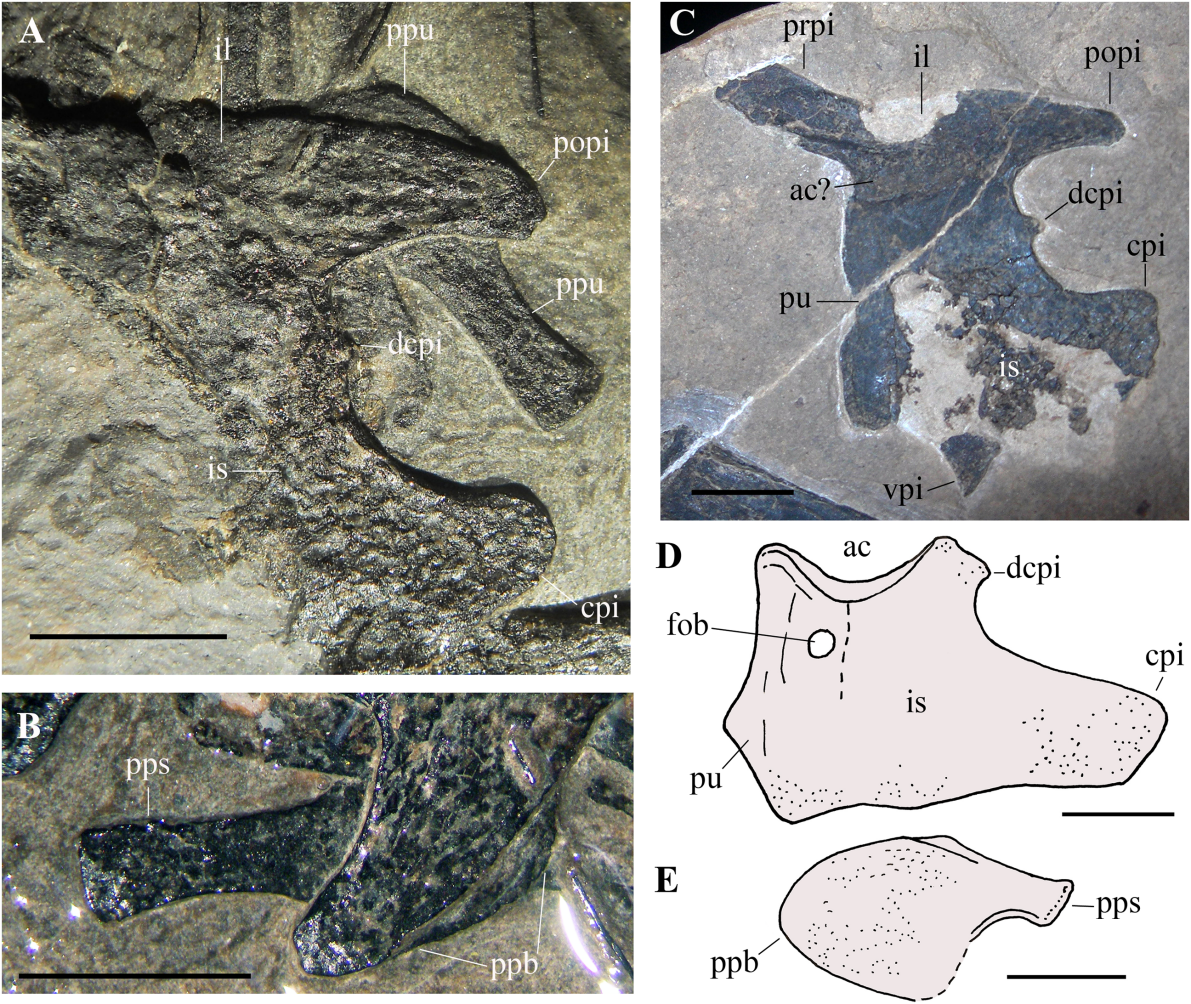
*Seazzadactylus venieri*, MFSN 21545 (holotype), pelvic elements and comparison. (A) Pelvic plate (caudal portion of ilium and ischium) and one prepubis of MFSN 21545; (B) prepubis of MFSN 21545 (photograph taken under ethanol immersion); (C) ?left pelvic plate of *Austriadraco dallavecchiai*, holotype (BSP 1994 I 51), ?lateral view; (D) drawing of the left ischiopubic plate of *Peteinosaurus zambellii* (MCSNB 3496), lateral view; (E) drawing of the left prepubis of *E. ranzii*, holotype (MCSNB 2888), lateral view. (D) and (E) are redrawn from Wild (1979). Dashed lines mark the margins of the missing parts. Abbreviations: ac, acetabulum; cpi, caudal process of ischium; dcpi, dorsocaudal process of ischium; fob, foramen obturatorium; il, ilium; is, ischium; popi, postacetabular process of ilium; ppb, prepubic blade; pps, prepubic stalk; ppu, prepubis; prpi, preacetabular process of ilium; pu, pubis; vpi, ventral process of ischium. Scale bar equals five mm.

**Pelvic plate.** The pelvic plates are close to the sacral vertebrae and the hind-limb elements (Figs. 1 and 2). One of them preserves the postacetabular process of the ilium and the upper part of the caudal process of the ischium, which have a similar caudal length (Fig. 23A). The postacetabular process is short, low, and slightly recurved ventrally; it tapers slightly distally to a blunt end. The caudal process of the ischium is short and its upper part has a rounded end that is slightly recurved dorsally. Both processes are similar to those of the holotype of *Austriadraco dallavecchiai* (Fig. 23C). The postacetabular process is also like that of *Carniadactylus rosenfeldi* (MPUM 6009, which does not preserve the caudal process of the ischium). The caudal process of the ischium is unlike the trapezoidal and more elongated process of *Peteinosaurus zambellii* (Fig. 23D). The caudal margin of the pelvic plate is deeply concave in MFSN 21545; a very small dorsocaudal process of the ischium occurs in the middle of the concavity as in the pelvic plate of the holotype of *Austriadraco dallavecchiai* (Fig. 23C) and *Peteinosaurus zambellii* (Fig. 23D). The ilium and ischium appear to be fused to one other but the pelvic plate and the sacrum were not fused to each other.

A broad, plate-like bone is partially preserved close to the described pelvic plate and the sacral vertebrae (Figs. 2 and 16). It is possibly the cranial portion of the other pelvic plate. It has a straight and vertical cranial (pubic) margin as in other pelvic plates of Triassic pterosaurs (cf. Fig. 23C), and also a spatula-like cranial process that could be the preacetabular process of ilium. However, this process is shorter and morphologically unlike the preacetabular process of all other pterosaurs (e.g. *Carniadactylus rosenfeldi*, Dalla Vecchia, 2014, fig. 4.1.145; *Austriadraco dallavecchiai*, Fig. 23C; MCSNB 8950, Wild, 1994, fig. 5; *Dimorphodon macronyx*, Sangster, 2003, fig. 3.15; *Dorygnathus banthensis*, Padian, 2008a, figs. 14, 19C and 21; *Campylognathoides liasicus*, Wellnhofer, 1974, fig. 9; *Campylognathoides* sp., Padian, 2008b, fig. 9; and *Rhamphorhynchus muensteri*, Wellnhofer, 1975a, fig. 10). Its shape could therefore be apomorphic, if it actually is the preacetabular process of the ilium.

**Prepubis.** A prepubis (10.5 mm long) is partly covered by the postacetabular process of an ilium (Fig. 23A). The exact outline of the expanded prepubic blade cannot be seen. However, this prepubic plate is probably shovel-like with a prepubic blade slightly more expanded ventrally than dorsally (Fig. 23B) like that of *E. ranzii* (Fig. 23E).

### Hind limb

Both right and left femora and tibiotarsi are partly preserved and the missing portions were restored (Figs. 1 and 2). Both femora are close and parallel to the corresponding tibiotarsi; both femur-tibiotarsus sets are close to one other and to the pelvic plates. No free tarsals can be identified. Only a few fragments are preserved of the elements of the foot.

**Femur.** The proximal third of the left femur (identified as such by its association with the left tibiotarsus) is preserved in cranial view, whereas the median third is missing and only a fragment remains of the distal third. The length of this preserved portion is 32 mm. In cranial view, the proximal head of the femur is dorsoventrally broad with only a hint of the neck (Fig. S7A). The angle between the proximal head of the femur and the shaft is 115°. The greater trochanter was damaged and has been reconstructed. Only a long and slightly sigmoidal portion of the shaft is preserved on the right femur. Because of the incompleteness of both elements, the exact total length of the femur cannot be known, but exceeds 32 mm.

**Tibiotarsus and fibula.** Right and left tibiotarsi are distinguished from one another on the basis of their position in the slightly disarticulated skeleton, the position of the associated fibula and the shape of their distal condyles (the lateral condyle is more developed than the medial one and projects more cranially than caudally in Triassic pterosaurs; Dalla Vecchia, 2003). Both tibiotarsi are strongly crushed and their shafts collapsed. The right tibiotarsus is exposed in caudal view and preserves the distal portion with the condyles (Fig. S7B) and fragments of most of the shaft with some fragments of the parallel and appressed fibula along the lateral side. The length of the preserved portion is 50 mm. The left tibiotarsus preserves the distal portion with the condyles and a proximal segment of the shaft. It is probable that it is exposed in medial view as the asymmetrical outline of the condyle (Fig. S7C) resembles that of the medial condyle of the left tibiotarsus of the holotype of *Carniadactylus rosenfeldi* (Dalla Vecchia, 2009a, fig. 9A; 2014, fig. 4.1.126 B-C); both elements also share a comma-like medial epicondyle. The proximal portion of the left tibiotarsus is covered by the blade of the left scapula, but part of its proximal end crops out from the ventral side of the blade where it is mostly concealed by vertebrae. The total length of the left tibiotarsus is approximately 55 mm.

**Pes.** Two phalanges and possibly a further two, all preserved close to the tibiotarsi (Figs. 1 and 2), belong to the completely disarticulated feet. The most complete phalanx is close to the left tibiotarsus and is short (4.9 mm) and stout.

## Phylogenetic Analysis

Unlike the data matrix of Britt et al. (2018), the state of the character 61 has been considered unknown for *Austriadraco dallavecchiai* here because of the dubious identification of the sternum in this taxon (see below). Codings of *Seazzadactylus venieri* are reported in the Supplemental Information. The analysis produced six equally parsimonious trees, each with a length of 290 steps, consistency index = 0.5759, homoplasy index = 0.5483, retention index = 0.7050, and rescaled consistency index = 0.4060. The analysis with TNT obtained the same topology and a tree length of 254 steps (polymorphic characters are treated as unknown by TNT).

**Figure 24:**
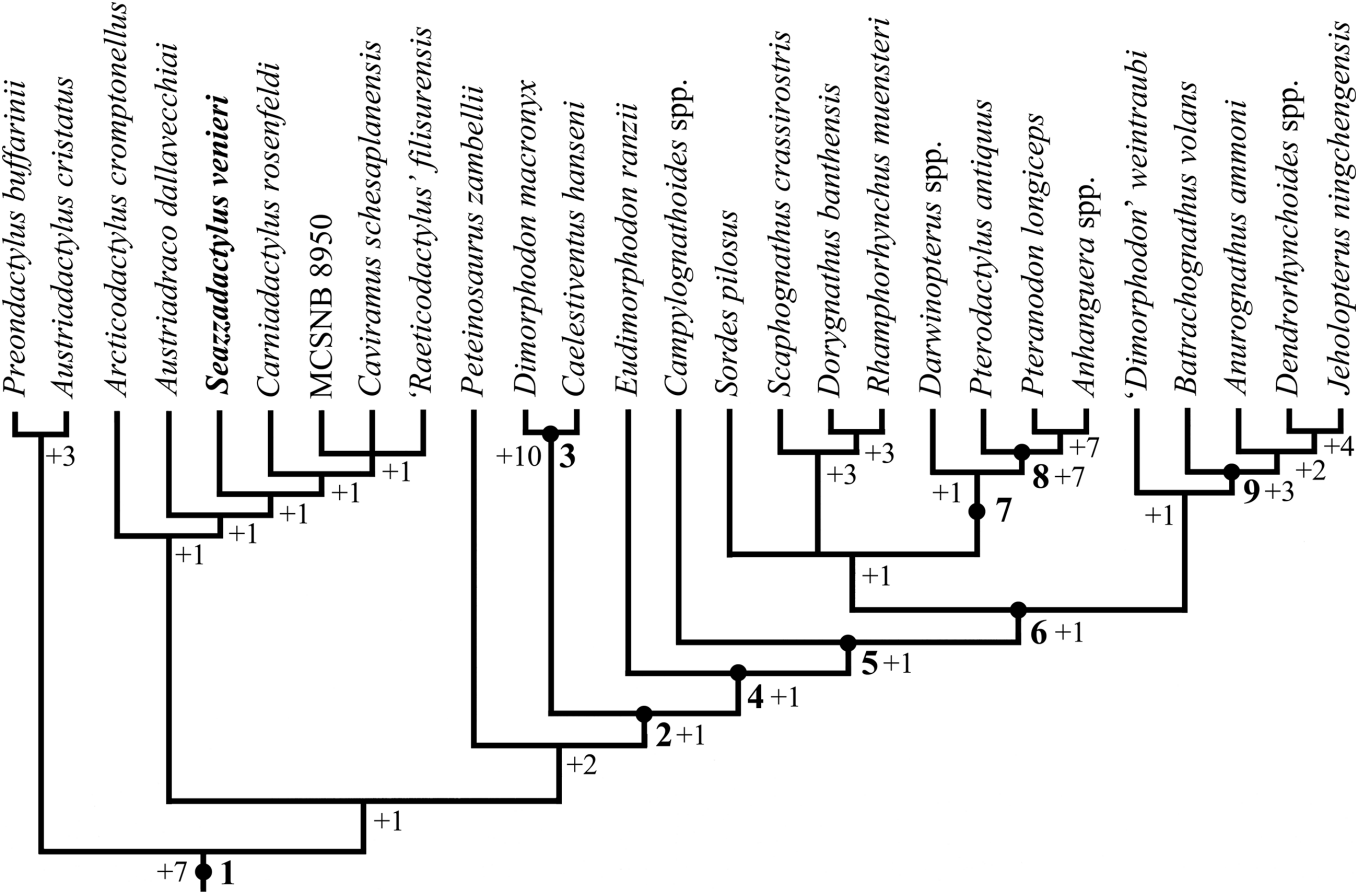
*Seazzadactylus venieri*, MFSN 21545 (holotype), phylogenetic relationships. Strict consensus tree of six most parsimonious trees as computed in PAUP 4.0b10 (length = 290, consistency index = 0.5759, RI = 0.7050) (see Materials, Terminology and Methods). Numbers +1 to +10 refer to Bremer values. Legend: **1**, Pterosauria; **2**, Macronychoptera; **3**, Dimorphodontidae; **4**, Lonchognatha; **5**, Novialoidea; **6**; Caelidracones; **7**, Monofenestrata; **8**, Pterodactyloidea; **9**, Anurognathidae. Outgroup taxa are not shown.

The strict consensus tree topology (Fig. 24) differs from that obtained by Britt et al. (2018; fig. 5, suppl. figs. 2-4). The addition of MFSN 21545 to the matrix including MCSNB 8950 resolved the big polytomy at the base of the strict consensus tree (see Britt et al., 2018, suppl. figs. 2-3). The basal clade of the Pterosauria is *Preondactylus buffarinii* + *Austriadactylus cristatus*, which is followed crownwards by an unnamed clade composed of *Arcticodactylus cromptonellus* + *Austriadraco dallavecchiai* + *Seazzadactylus venieri* + *Carniadactylus rosenfeldi* + (trichotomy of *Raeticodactylus filisurensis*, *Caviramus schesaplanensis* and MCSNB 8950). *Seazzadactylus venieri* is nested within this unnamed clade as the sister taxon of *Carniadactylus rosenfeldi* + (trichotomy of *Raeticodactylus filisurensis*, *Caviramus schesaplanensis* and MCSNB 8950). There is no support for a clade Eudimorphodontidae sensu Dalla Vecchia (2014), because *E. ranzii* is located in the tree between the Dimorphodontidae and *Campylognathoides* spp. Bremer support values for the clades in the analysis are mostly low: all nodes within the unnamed clade mentioned above have Bremer values of +1 (Fig. 24). Only Pterosauria, *Preondactylus buffarinii* + *Austriadactylus cristatus*; Dimorphodontidae; Anurognathidae; *Jeholopterus ningchengensis* + *Dendrorhynchoides* spp.; *Dorygnathus banthensis* + Rhamphorhynchidae; Rhamphorhynchidae; Pterodactyloidea; and *Anhanguera* spp. + *Pteranodon longiceps* have Bremer values ≥3.

## Discussion

### Ontogenetic stage

MFSN 21545 is similar in size to the holotype of *Carniadactylus rosenfeldi*. It is slightly larger than the holotype of *Austriadraco dallavecchiai* and is nearly twice the linear size of the specimen MPUM 6009 of *Carniadactylus rosenfeldi* (Tables S1 and S3). MFSN 21545 is larger than specimens MCSNB 2887 and MCSNB 8950 and is much larger than the holotype of *Arcticodactylus cromptonellus*. Although the mean of the percent length of some selected skeletal elements of the holotype of *E. ranzii* is only 118% those of MFSN 21545 (Table S3), the latter appears much smaller when the fossils are compared (Fig. S8), because body mass is proportional to the length raised to the third power. MFSN 21545 is much smaller than the holotype of *Raeticodactylus filisurensis*.

The holotypes of *Carniadactylus rosenfeldi* and *Austriadraco dallavecchiai* are not juveniles, although the holotype of *Austriadraco dallavecchiai* shows some features of osteological immaturity (Dalla Vecchia, 2018). Osteological features of immaturity also occur in MPUM 6009 and MCSNB 8950 (Dalla Vecchia, 2018). The holotype of *Arcticodactylus cromptonellus* is a young individual, based on histological analysis (Padian, Horner & De Ricqlés, 2004). The holotype of *E. ranzii* is usually considered an adult, but it also shows some features indicating osteological immaturity (Dalla Vecchia, 2018). The holotype of *Raeticodactylus filisurensis* does not show any features of osteological immaturity (F.M. Dalla Vecchia, 2018, personal observation), but it is incomplete (e.g. pelvis and sacrum are not preserved).

Some features suggest osteological immaturity also for MFSN 21545. Roof and palatal elements of the skull are unfused. A suture is visible between the premaxillae. The mandibular rami are unfused at the symphysis (but see Dalla Vecchia, 2018). There are possibly three unfused distal carpals (but see Dalla Vecchia, 2009a, 2018). The ilium is not fused to the sacral ribs, which are not fused with the sacral vertebrae. Prepubes are not fused at the symphysis. On the other hand, the elements of the occipital region and basicranium appear to be fused, although is impossible to state whether the sutures among the elements were fully obliterated or not. The neural arches of the last dorsal vertebrae appear to be fused to their centra. The sternum is ossified with processes for the sternal ribs. Scapulae and coracoids are fused. There is a single proximal syncarpal. The extensor tendon of the first wing phalanx is fused to the phalanx (however, this occurred early during ontogeny in Triassic pterosaurs; Dalla Vecchia, 2018, contra Kellner, 2015). The phalanges of the manus are well-ossified with well-formed ginglymi. The ilium is fused with the puboischiadic plate and the sacral vertebrae are fused into a synsacrum. The fusion of the sacral vertebrae seems to have occurred relatively late during ontogeny in Triassic pterosaurs (Kellner, 2015; Dalla Vecchia, 2018). The rounded condylar end of the tibia indicates that it is actually a tibiotarsus with the proximal tarsals fused to the tibia. These features indicate that MFSN 21545 was not a juvenile, but probably was still growing when it died (Dalla Vecchia, 2018).

### Systematic comparisons

Multicusped maxillary and mandibular teeth like those of *Seazzadactylus venieri* are reported only in the Triassic taxa *E. ranzii*, *Carniadactylus rosenfeldi*, *Arcticodactylus cromptonellus* and *Austriadraco dallavecchiai* (Dalla Vecchia, 2014). *Caviramus schesaplanensis* and *Raeticodactylus filisurensis* also have multicusped teeth, but their crowns are distinctly bulkier than those of *Seazzadactylus venieri* and have a peculiar constriction at their base (Dalla Vecchia, 2014, figs. 4.1.57A–C and 4.1.161B–C). Two other specimens (MCSNB 2887 and MCSNB 8950) do not preserve any trace of dentition, but they have been considered closely related to the taxa listed above in the literature (Wild, 1979, 1994). *Seazzadactylus venieri* must first be compared with specimens of *Carniadactylus rosenfeldi* because the holotype of this species comes from the same formation and geographic region (Dalla Vecchia, 2009a). Although both pterosaurs are from the Dolomia di Forni Formation, it cannot be known whether they are from the same stratigraphic level or not, as the exact stratigraphic provenance of MFSN 21545 is unknown and the fossiliferous part of the Dolomia di Forni Formation is about 500 m thick (Dalla Vecchia, 2006). The holotype of *Carniadactylus rosenfeldi* comes from a stratigraphically mid-low position within the Dolomia di Forni Formation (see Dalla Vecchia, 2014) and the boulder containing MFSN21545 was located in the lower part of the formation.

#### Cranial bones

**Premaxilla.** The body of the premaxilla is more elongated in *Seazzadactylus venieri* than in *Carniadactylus rosenfeldi* (MPUM 6009), and teeth occur only in the rostral half of the premaxilla, whereas the last premaxillary tooth is at the premaxilla/maxilla boundary in MPUM 6009 (Dalla Vecchia, 2018, fig. 2). No other non-monofenestratan pterosaurs have premaxillary teeth restricted to the rostral half of the premaxilla (i.e. *Preondactylus buffarinii*, Dalla Vecchia, 2014, fig. 4.1.3; *Austriadactylus cristatus*, Dalla Vecchia et al., 2002, fig. 2; *Raeticodactylus filisurensis*, Stecher, 2008, fig. 6; *E. ranzii*, Wild, 1979, fig. 1; *Dimorphodon macronyx*, Sangster, 2003, figs. 1.6A and 2.1A; *Dorygnathus banthensis*, Padian, 2008a, fig. 18); *Campylognathoides liasicus* and *Campylognathoides zitteli*, Padian, 2008a, fig. 10; *Scaphognathus crassirostris*, Bennett, 2014, fig. 5; *Jianchangopterus zhaoianus*, Lü & Bo, 2011, fig. 3; *Jianchangnathus robustus*, Zhou, 2014, fig. 3; *Rhamphorhynchus muensteri*, Wellnhofer, 1975a, fig. 3); *Anurognathus ammoni*, Bennett, 2007, fig. 4; and *Batrachognathus volans*, Riabinin, 1948, fig. 1).

**Maxilla.** The maxilla of *Seazzadactylus venieri* has an elongated foramen at the base of the ascending process in lateral view, which is lacking in *Carniadactylus rosenfeldi*, *Arcticodactylus cromptonellus, E. ranzii* and *Raeticodactylus filisurensis*. Unlike *Arcticodactylus cromptonellus*, the maxilla of *Seazzadactylus venieri* lacks a row of large foramina along the lateral side of the jugal process (Jenkins et al., 2001, fig. 4). The maxillary process of the jugal overlaps the jugal process of the maxilla laterally in *Seazzadactylus venieri*, whereas the jugal overlaps the jugal process of the maxilla dorsally in *Carniadactylus rosenfeldi* (MPUM 6009; Dalla Vecchia, 2018, fig. 2) and *E. ranzii* (see Wild, 1979, figs. 1 and 25b). *Seazzadactylus venieri* lacks the small notch for the maxillary process of the premaxilla that occurs on the dorsal margin of the premaxillary processes of the maxilla of *E. ranzii* (see Dalla Vecchia, 2014, Fig. 4.1.82).

**Jugal.** The jugal of *Seazzadactylus venieri* differs from that of *Carniadactylus rosenfeldi* (MPUM 6009; Wild, 1979, fig. 2; Dalla Vecchia, 2018, fig. 2), as well as those of other non-monofenestratan pterosaurs (i.e. *Austriadraco dallavecchiai*, Wellnhofer, 2003, fig. 3A; fig. 7C; *E. ranzii*, Wild, 1979, fig. 1; *Raeticodactylus filisurensis*, Stecher, 2008, fig. 6; *Dimorphodon macronyx*, Sangster, 2003, fig. 2.7; *Caelestiventus hanseni*, Britt et al., 2018, fig. 3h and i; *Parapsicephalus purdoni*, Newton, 1888, pl. 78, fig. 2; O’Sullivan & Martill, 2017, fig. 5A; *Campylognathoides liasicus*, Wellnhofer, 2003, fig. 3C; *Dorygnathus banthensis*, Wellnhofer, 2003, fig. 3D; *Scaphognathus crassirostris*, Wellnhofer, 1975b, fig. 33a and c; Bennett, 2014, fig. 5A; *Rhamphorhynchus muensteri*, Wellnhofer, 1975a, fig. 3a; and *Rhamphinion jenkinsi*, Padian, 1984, fig. 1). The jugal of *Austriadraco dallavecchiai* has a long, slender, and caudally sloping postorbital process and a very small lacrimal process like those of *Seazzadactylus venieri*, but the jugal of *Austriadraco dallavecchiai* has a comparatively shorter body, the lacrimal process is more inclined rostrally, and the maxillary process is much smaller (Fig. 7C). The jugal of *Seazzadactylus venieri* is comparatively more slender than the jugal of *Carniadactylus rosenfeldi* (MPUM 6009), *E. ranzii* and *Raeticodactylus filisurensis* too. The postorbital process of the jugal of *Seazzadactylus venieri* is more slender and proportionately longer than that of *E. ranzii* and *Raeticodactylus filisurensis*; the condition in *Carniadactylus rosenfeldi* (MPUM 6009) is unclear because the distal part of the process is not exposed (see Dalla Vecchia, 2018, fig. 2). The lacrimal process of the jugal of *Seazzadactylus venieri* is smaller and more inclined rostrally than those of *Carniadactylus rosenfeldi* (MPUM 6009), *E. ranzii* and *Raeticodactylus filisurensis*, whereas the maxillary process is deeper. The jugal contributes to an antorbital fossa in *E. ranzii* (Wild, 1979 identified it as the lacrimal; see Witmer, 1997, p. 33) and possibly also in *Carniadactylus rosenfeldi* (see Dalla Vecchia, 2018). There is no large foramen in the middle of the jugal body in *Carniadactylus rosenfeldi* as well as in all other pterosaurs.

**Postorbital.** *Seazzadactylus venieri* shares with *Carniadactylus rosenfeldi* and *Austriadraco dallavecchiai* a slender Y-like postorbital (Dalla Vecchia, 2018, fig. 3A-B). The postorbital in *E. ranzii* is comparatively shorter than in *Seazzadactylus venieri*, has stouter rami, more open frontal and postorbital rami and a distally expanded frontal ramus.

**Pterygoid.** As far as can be seen in the holotype (Dalla Vecchia, 2009a, figs. 2A), the pterygoid of *Carniadactylus rosenfeldi* is unlike that of *Seazzadactylus venieri*. The pterygoid of *E. ranzii* has small tooth-like structures on the palatal side (Wild, 1979; Dalla Vecchia, 2014, fig. 4.1.75) that are absent in the pterygoids of *Seazzadactylus venieri*.

#### Cranial fenestrae

Because of the orientation of the lacrimal process of the jugal, the caudal portion of the antorbital fenestra was deeper in *Carniadactylus rosenfeldi* (MPUM 6009), *E. ranzii* and *Raeticodactylus filisurensis* than in *Seazzadactylus venieri*. The antorbital fenestra of *Seazzadactylus venieri* probably has the outline of an isosceles triangle, whereas that of *Carniadactylus rosenfeldi* is probably D-like or oval and that of *E. ranzii* is D-like. The caudal corner of the antorbital fenestra of *Seazzadactylus venieri* is not slit-like as that of *Austriadraco dallavecchiai* (Fig. 7C). Because of the shape of the postorbital, the upper temporal fenestra probably had the outline of an inverted tear-drop in *Seazzadactylus venieri*, *Carniadactylus rosenfeldi* and *Austriadraco dallavecchiai*, whereas it likely had a sub-circular outline in *E. ranzii*.

#### Mandible

*Seazzadactylus venieri* shares with *Carniadactylus rosenfeldi* the straight and pointed rostral end of the dentary. By contrast, the rostral end of the dentary is blunt and bent downwards in *E. ranzii* (Wild, 1979, fig. 1). *Seazzadactylus venieri* shares with *Carniadactylus rosenfeldi* (see Dalla Vecchia, 2009a, fig. 2) and *Austriadraco dallavecchiai* (Fig. 10E) the longitudinal arched ridge bordered by narrow ventral and dorsal grooves on the labial side of the dentary, and the retroarticular process of the mandible that is lateromedially flattened and has a rounded profile in lateral view. *E. ranzii* lacks this longitudinal arched ridge. *Seazzadactylus venieri*, in common with all other pterosaurs, lacks the external mandibular fenestra than occurs in *Austriadraco dallavecchiai* (see Bennett, 2015).

The dorsal margin of the mandibular ramus between the distalmost tooth and the cotyle for the quadrate has the same saddle-like (two-peaked) outline in both *Seazzadactylus venieri* and *Austriadraco dallavecchiai* (Fig. 10), distinguishing them from all other pterosaurs (Dalla Vecchia, 2009a, 2014). Both have a rounded dorsal process of the surangular (‘coronoid’ process). The dorsal process of the surangular is triangular and pointed in *Carniadactylus rosenfeldi* and the dorsal margin of the lower jaw between the distalmost tooth and the cotyle for the quadrate is markedly angled (Dalla Vecchia, 2009a, figs. 2B and 11). In *E. ranzii* (Wild, 1979, fig. 4), the dorsal margin of the mandibular ramus between the distalmost tooth and the cotyle for the quadrate presents only a small, triangular and pointed dorsal process of the surangular that is very close to the last mandibular tooth.

#### Dentition

The multicusped teeth of *Seazzadactylus venieri*, *Carniadactylus rosenfeldi*, *Austriadraco dallavecchiai* and *Arcticodactylus cromptonellus* have smooth crown surfaces in contrast to those of *E. ranzii* which possess basoapical ridges. The main cusps in the multicusped crowns of *Seazzadactylus venieri* are proportionally broader mesiodistally than those of *E. ranzii* and are probably also more flattened labiolingually (Dalla Vecchia, 2014, fig. 4.1.78). Upper accessory cusps of the pentacuspid teeth of *Seazzadactylus venieri* are less robust than those of the pentacuspid teeth of *E. ranzii*.

**Premaxillary teeth.** Premaxillary teeth of *Seazzadactylus venieri* resemble a displaced tooth of *Austriadraco dallavecchiai* that was tentatively identified as a premaxillary tooth by Wellnhofer (2003) based on its general shape and ornamentation. That tooth is associated with the impression of the premaxillae (F.M. Dalla Vecchia, 2018, personal observation), therefore I consider it to be a premaxillary tooth here. It differs from premaxillary teeth of *Seazzadactylus venieri* in having a constriction between the crown and ‘root’ (Wellnhofer, 2003, fig. 4B). All premaxillary teeth are unicuspid in *Seazzadactylus venieri*, whereas the premaxillary tooth 3 is tricuspid in *E. ranzii* (Wild, 1979, fig. 8a).

**Maxillary teeth.** *Seazzadactylus venieri*, *Carniadactylus rosenfeldi* (MPUM 6009), *Arcticodactylus cromptonellus* and *E. ranzii* have 14, 14, ∼12 and 25 maxillary teeth, respectively. Therefore, *Seazzadactylus venieri* has a comparatively lower tooth-count with respect to body size (Table S3).

Corresponding multicusped crowns are approximately the same size in the maxilla and mandible of *E. ranzii*, *Carniadactylus rosenfeldi* and *Arcticodactylus cromptonellus*, whereas the maxillary crowns are larger than the corresponding mandibular crowns in *Seazzadactylus venieri* and *Raeticodactylus filisurensis*. This size difference is even more marked in *Preondactylus buffarinii* and *Austriadactylus cristatus* (see Dalla Vecchia, 2014). One displaced tooth of *Austriadraco dallavecchiai* figured by Wellnhofer (2003, fig. 4A) was considered as one of the first mandibular teeth or a fang-like tooth from below the ascending process of the maxilla. However, its denticulated crown is not much larger than the crowns of the multicusped mandibular teeth (its apicobasal height is at maximum two mm, whereas that of a multicusped mid-mandibular crown is ca. 1.25 mm; see Wellnhofer, 2003). This suggests that it is a maxillary tooth (Dalla Vecchia, 2009a, 2014) but not a fang-like one; thus *Austriadraco dallavecchiai* probably had maxillary teeth that were slightly larger than its mandibular teeth like *Seazzadactylus venieri*.

*Seazzadactylus venieri*, *Carniadactylus rosenfeldi* (MPUM 6009) and *Arcticodactylus cromptonellus* lack the large fang-like teeth below the ascending process of the maxilla that occur in *E. ranzii*, *Preondactylus buffarinii* and *Austriadactylus cristatus* (see Dalla Vecchia, 2014).

The maxillary teeth of *E. ranzii* are more closely spaced than the fully erupted maxillary teeth of *Seazzadactylus venieri* (cf. Wild, 1979, fig. 1 and Fig. 13).

Unlike *Carniadactylus rosenfeldi*, *Arcticodactylus cromptonellus* and *E. ranzii, Seazzadactylus venieri* has hexacuspid and heptacuspid maxillary teeth. Also its overall cuspidation pattern is unlike that of these pterosaurs. The maxillary teeth of MPUM 6009 are penta- and tetracuspid (as in the case of *Seazzadactylus venieri*, there are no fully grown tricuspid teeth; Wild, 1979, figs. 2, 6 and 27a). An exception is tooth 3, which is apparently unicuspid with a straight cusp; this may be an erupting tooth, which Wild (1979, fig. 27a) considered to be a tricuspid or unicuspid tooth when fully erupted. The first two maxillary teeth preserved in *Arcticodactylus cromptonellus* are unicuspid and the others have 3–5 cusps without any apparent trend. According to Wild (1979), 11 maxillary teeth mesial to the fang-like teeth are mostly tricuspid (left crown 5 is tetracuspid) whereas 12 distal to the fang-like teeth are pentacuspid in *E. ranzii*.

The maxillary teeth of *Seazzadactylus venieri* show further morphological differences from those of *Austriadraco dallavecchiai*, *Arcticodactylus cromptonellus* and *E. ranzii*. Unlike in *Seazzadactylus venieri*, the crown of the maxillary tooth of *Austriadraco dallavecchiai* is much apicobasally higher than mesiodistally long and bears three small accessory cusps plus two crenulations along each cutting margin (Wellnhofer, 2003, fig. 4a). The crown of the most mesial preserved tooth of *Arcticodactylus cromptonellus* is unicuspid and smaller than the following crowns like that of the first maxillary tooth of *Seazzadactylus venieri*, but it is not recurved backwards (see Jenkins et al., 2001, fig. 4). The first four maxillary crowns of *E. ranzii* are not recurved backwards like those of *Seazzadactylus venieri* and the first crown is tricuspid (Wild, 1979, fig. 25b) and lacks the inflated basal portion of the corresponding crown of *Seazzadactylus venieri*.

**Mandibular teeth.** *Seazzadactylus venieri*, *Carniadactylus rosenfeldi* (MPUM 6009) and *E. ranzii* have 21, 17 or18 and 26–28 mandibular teeth, respectively. The mandibular tooth-count is more proportional to the body size of these specimens (Table S3) than the maxillary tooth-count. The ratio of the tooth number/mandibular length is similar in *Seazzadactylus venieri* and *E. ranzii* (0.39 and 0.38, respectively), while it is higher in *Carniadactylus rosenfeldi* (0.50–0.53).

The first two, unicuspid, mandibular teeth of *Seazzadactylus venieri* are stouter than those of *Carniadactylus rosenfeldi* and *E. ranzii* and are comparatively smaller than those of *E. ranzii*. Tooth 1 is procumbent, but the second is not in *E. ranzii*. The third mandibular tooth is heptacuspid in *Seazzadactylus venieri*, whereas it is fang-like with a distal accessory cusp in *Carniadactylus rosenfeldi* (see Dalla Vecchia, 2014, fig. 4.1.140A-B) and very small and tricuspid in *E. ranzii*. The most mesial preserved mandibular crown of *Arcticodactylus cromptonellus* is unicuspid but not larger than the following crowns and not recurved (Jenkins et al., 2001, fig. 3).

Unlike *Carniadactylus rosenfeldi*, *Austriadraco dallavecchiai*, *Arcticodactylus cromptonellus* and *E. ranzii, Seazzadactylus venieri* has hexacuspid and heptacuspid mandibular teeth. Furthermore, the overall cuspidation pattern of *Seazzadactylus venieri* is unlike that of these other pterosaurs. In *Carniadactylus rosenfeldi*, teeth distal to tooth 3 are multicusped with a predominance of tricuspid crowns mesially and of pentacuspid crowns distally (Wild, 1979, fig. 27a). In *Austriadraco dallavecchiai* the most mesial preserved right crown is tricuspid, while the following 11 preserved teeth are pentacuspid (Wellnhofer, 2003, fig. 4b). The left ramus preserves the last 11 tooth positions and 10 teeth. The last four teeth are tricuspid, the first two are tetracuspid and the others are pentacuspid (Wellnhofer, 2003). The fragments of the mandibular rami of *Arcticodactylus cromptonellus* each bear 11 teeth (Jenkins et al., 2001). The most mesial preserved crown of the left ramus is unicuspid and the following is bicuspid, while the other teeth are tricuspid with the exception of the penultimate, which is pentacuspid. The first preserved tooth of the right ramus (the most mesial teeth are missing) is tetra- or pentacuspid; the second is tricuspid; the third is tetra- or pentacuspid; the fourth is tricuspid but erupting; the fifth is tetracuspid; and the six most distal crowns are pentacuspid except for the eighth preserved crown, which is tricuspid (but possibly not fully grown). The multicusped mandibular teeth of *E. ranzii* show a predominance of tricuspid teeth mesially and of pentacuspid teeth distally; one crown is tetracuspid and one bicupid (Wild, 1979, fig. 27b).

In *Seazzadactylus venieri*, the first two multicusped crowns of the mandible differ from the following crowns, whereas they have the same shape in *E. ranzii*. Unlike in *Seazzadactylus venieri*, the first seven multicusped crowns of *E. ranzii* are much smaller than the mid-mandibular teeth.

#### Sternum

The general shape of the sternum is similar in *Seazzadactylus venieri* and *E. ranzii* (see Wild, 1979). A rhomboid skeletal element in *Austriadraco dallavecchiai* was identified as a sternum by Wellnhofer (2003), but it was later referred to the fused frontals by Bennett (2015) and Kellner (2015). If the bone is the frontal plate, no comparison is possible with *Seazzadactylus venieri*. If it is the sternum, it much differs from the sternum of *Seazzadactylus venieri*. Unlike in *Seazzadactylus venieri*, the sternum of MCSNB 8950 lacks the processes for the sternal ribs (Wild, 1994).

#### Pectoral girdle

**Coracoid.** *Seazzadactylus venieri* shares with *Carniadactylus rosenfeldi* a plate-like, flat and broad shaft of the coracoid with parallel cranial and caudal margins, which is a diagnostic feature of *Carniadactylus rosenfeldi* according to Dalla Vecchia (2009a). The coracoid of *Arcticodactylus cromptonellus* also appears to have a relatively flat and broad shaft (Jenkins et al., 2001). In contrast, the shaft of the coracoid is strut-like in *E. ranzii* (see Wild, 1979, fig. 15) and Jurassic pterosaurs. The coracoid of *Austriadraco dallavecchiai* is flat and broad like that of *Seazzadactylus venieri*, but fan-shaped (Wellnhofer, 2003, fig. 12)—i.e. its cranial and caudal margins diverge distally unlike those of *Seazzadactylus venieri* and *Carniadactylus rosenfeldi*. The shaft of the coracoid of MCSNB 2887 is not as broad and flat as in *Seazzadactylus venieri*.

**Scapula.** Although only the proximal portion of the scapula is preserved in specimens of *Carniadactylus rosenfeldi*, the parallel margins and the narrowness of the scapula of MPUM 6009 (Dalla Vecchia, 2014, fig. 4.1.99B-C) show that this bone did not flare like the scapulae of *Seazzadactylus venieri* (where flaring starts proximally). The scapular blade is distally expanded with a rounded end in *Austriadraco dallavecchiai*, but the rest of the blade is narrow, with parallel margins (Wellnhofer, 2003, fig.12), unlike the scapulae of *Seazzadactylus venieri*. The scapular blade is not expanded in *Arcticodactylus cromptonellus* (see Jenkins et al., 2001, fig. 2), *E. ranzii* (see Wild, 1979, fig. 15), MCSNB 8950 (Wild, 1994, fig. 2) and MCSNB 2887 (Dalla Vecchia, 2014, fig. 4.1.167). The flaring of the scapular blade of *Seazzadactylus venieri* is not observed in any other Triassic or Jurassic pterosaur (e.g., Goldfuss, 1831, pl. 8; Sharov, 1971, fig. 1; Wellnhofer, 1975a, fig. 9; 1978, fig. 9; Wild, 1979, fig. 34; Ji & Ji, 1998, fig. 1; Wang, Zhou & Zhang, 2002, fig. 1; Bennett, 2003, fig. 3A-B, 2007, fig. 2; Sangster, 2003, fig. 3.2; Padian, 2008a, fig. 21, 2008b, fig. 11; Lü et al., 2012, fig. 2a; Cheng et al., 2014, fig. 1; Zhou, 2014, fig. 4; Lü et al., 2017, fig. 2).

#### Forelimb

**Pteroid.** The question mark-like pteroid of *Seazzadactylus venieri* is distinct from the angled and boomerang-like pteroid of *Carniadactylus rosenfeldi* (see Dalla Vecchia, 2009a, fig. 6A-B) and the handle-like pteroid of *E. ranzii* (see Dalla Vecchia, 2009a, fig. 6D). The pteroid of MCSNB 2887 is also handle-shaped (Dalla Vecchia, 2014, fig. 4.1.170) and proportionally larger than the small pteroid of *Seazzadactylus venieri*. The pteroid of MCSNB 8950 (Wild, 1994, figs. 2 and 6) is rod-like, recurved and more robust than that of *Seazzadactylus venieri*. The pteroid of *Seazzadactylus venieri* is also unlike those of *Preondactylus buffarinii* (see Dalla Vecchia, 2014, fig. 4.1.5); *Peteinosaurus zambellii* (see Dalla Vecchia, 2014, figs. 4.1.65-66); *Dimorphodon macronyx* (see Sangster, 2003, p. 72, figs. 3.8 and 3.10); *Campylognathoides liasicus* (see Wellnhofer, 1974, fig. 8a; Wild, 1975, fig. 4); *Campylognathoides zitteli* (see Wild, 1975, fig. 3); *Dorygnathus banthensis* (see Padian, 2008a, figs. 3 and 13A, pl. 4/fig. 5); *Scaphognathus crassirostris* (see Wellnhofer, 1975a, fig. 2; Bennett, 2014, fig. 3A); *Quinglongopterus guoi* (see Lü et al., 2012, fig. 2b); *Orientognathus chaoyangensis* (see Lü et al., 2015, fig. 4b); *Bellobrunnus rothgaengeri* (see Hone et al., 2012, fig. 10); *Rhamphorhynchus muensteri* (see Wellnhofer, 1975a, fig. 12a-b); *Anurognathus ammoni* (see Wellnhofer, 1975b, fig. 37) and *Vesperopterylus lamadongensis* (see Lü et al., 2017, fig. 2).

**Metacarpus.** In *Seazzadactylus venieri*, metacarpal III = II > I (as in *Preondactylus buffarinii*), whereas metacarpal III > II > I in *Carniadactylus rosenfeldi*, *Arcticodactylus cromptonellus* and *E. ranzii* (Table S1).

**Wing phalanges.** Wing phalanx 1 is straight in *Seazzadactylus venieri*, whereas it is slightly curved cranially in *Carniadactylus rosenfeldi* (see Dalla Vecchia, 2009a) and *Austriadraco dallavecchiai* (see Wellnhofer, 2003, fig. 15A). Wing phalanx 1 of *Seazzadactylus venieri* is comparatively shorter than that of *Carniadactylus rosenfeldi* (see ratios wing phalanx 1/humerus, wing phalanx 1/ulna and wing phalanx 1/metacarpal IV in Table S4). In *Seazzadactylus venieri*, wing phalanx 3 > 2 > 1 as in MCSNB 8950 and in the Jurassic *Dimorphodon macronyx* and *Sordes pilosus* (Dalla Vecchia, 2014, p. 306), while the proportions are 1 > 3 > 2 in both *Carniadactylus rosenfeldi* specimens and 2 = 3 < 1 in *Arcticodactylus cromptonellus*.

#### Pelvic girdle

**Ilium and ischium.** The postacetabular process of the ilium of *Seazzadactylus venieri* has the same outline as that of *Carniadactylus rosenfeldi* (MPUM 6009; Dalla Vecchia, 2014, fig. 4.1.145). The postacetabular process of the ilium and the preserved portion of the caudal process of the ischium of *Seazzadactylus venieri* resemble those of *Austriadraco dallavecchiai* (Fig. 23C). Unlike in *Seazzadactylus venieri*, the postacetabular process of MCSNB 8950 is straight and distally pointed in lateral view (Wild, 1994, fig. 5). However, these processes are not preserved in *E. ranzii*, *Arcticodactylus cromptonellus*, *Caviramus schesaplanensis*, *Raeticodactylus filisurensis* and *Caelestiventus hanseni* and the postacetabular process of the ilium is not preserved in *Austriadactylus cristatus* and *Peteinosaurus zambellii*. The morphological variability of these processes within the Triassic pterosaurs is therefore unknown because of the incompleteness of the fossil record.

**Prepubis.** Although the prepubis of *Seazzadactylus venieri* is plausibly similar to that of *E. ranzii*, it has a proportionally longer stem (see Figs. 23B and 23E). Unlike in *Seazzadactylus venieri*, the prepubic blade of MCSNB 8950 is squared (Wild, 1994, fig. 5).

#### Hind-limb

**Femur.** The femur of *Seazzadactylus venieri* is similar to the left femur of the holotype of *Austriadraco dallavecchiai*, which has an angle between the proximal head of the femur and shaft that is also about 115° (F.M. Dalla Vecchia, 2018, personal observation), unlike the femur of *Raeticodactylus filisurensis* where it is closer to 90° (Stecher, 2008).

**Tibia.** The tibia is as robust as the radius and ulna in *Seazzadactylus venieri* and *Carniadactylus rosenfeldi*, whereas it is more gracile than the radius and ulna in *E. ranzii* (see Wild, 1979, pl. 2). The tibia is proportionately shorter in MCSNB 2887 with respect to *Seazzadactylus venieri*, as shown by the ratios humerus/tibia, ulna/tibia and wing phalanx1/tibia (Table S4).

#### Ratios of long-bone lengths

The ratios wing phalanx 1/humerus and wing phalanx 1/metacarpal IV are much higher in *Seazzadactylus venieri* (1.38 and 2.92, respectively) than in *Arcticodactylus cromptonellus* (0.95 and 2.24, respectively; Table S4). The wing metacarpal and tibia are proportionally shorter in MCSNB 8950 than in *Seazzadactylus venieri*, as shown by the ratios humerus/metacarpal IV, ulna/metacarpal IV and wing phalanx1/metacarpal IV and humerus/tibia, ulna/tibia and wing phalanx1/tibia, respectively (Table S4).

### Significance of the new taxon

The addition of *Seazzadactylus venieri* to the matrix of Britt et al. (2018) caused a significative change in the topology of the strict consensus tree (Fig. 24) with respect to the original analysis (Britt et al., 2018, fig. 5). *Austriadraco dallavecchiai* and *Arcticodactylus cromptonellus* are nested within the same clade as *Carniadactylus rosenfeldi*, whereas they formed a separate clade in the strict consensus tree of Britt et al. (2018). Furthermore, *E. ranzii* is recovered as the basalmost taxon of the Lonchognatha and more derived than all other pterosaurs with multicusped dentition (which are all Triassic in age like *E. ranzii*). Therefore, *E. ranzii* would have developed its multicusped dentition independently or retained it as an ancestral feature. However, the latter hypothesis is less parsimonious because implies two steps more than the former.

The phylogenetic analysis supports the close relationships of *Seazzadactylus venieri* with *Carniadactylus rosenfeldi* and *Austriadraco dallavecchiai*, but also its separation as a distinct taxon within an unnamed clade of Triassic taxa also including *Arcticodactylus cromptonellus* and the trichotomy formed by MCSNB 8950, *Caviramus schesaplanensis* and *Raeticodactylus filisurensis*. Forcing *Seazzadactylus venieri* as the sister taxon of *Carniadactylus rosenfeldi*, the shortest topologies found are two steps longer than the non-forced shortest trees. Forcing *Seazzadactylus venieri* to be the sister taxon of *Austriadraco dallavecchiai*, the shortest topologies found are only one steps longer than the non-forced shortest trees. However, *Austriadraco dallavecchiai* is clearly distinct from *Seazzadactylus venieri* in the following features: a different maxillary process of the jugal; a stouter body of the jugal and the absence of the large foramen in the middle; a slit-like caudal part of the antorbital fenestra; the presence of an external mandibular fenestra; the presence of a constriction between the crown and ‘root’ in the premaxillary teeth; crowns of maxillary multicusped teeth that are much apicobasally higher than mesiodistally long; a different cuspidation pattern in the mandibular dentition; a narrower scapular blade; and distally diverging cranial and caudal margins of the shaft of the coracoid. These differences are not ontogenetic because the two holotypes appear to be at a similar ontogenetic stage (Dalla Vecchia, 2018). The potential for a sexually-dimorphic relationship between this trait disparity could only be tested with a larger sample, preferably from the same population.

It is premature to give a name to and a definition of the clade containing *Seazzadactylus venieri*, because the Bremer values for the nodes within it are low (Fig. 24). This instability is probably due to the non-overlapping known skeletal remains for many Triassic taxa, which are mostly represented by single and fragmentary specimens. Tree topology could change with the discovery of further specimens of the included terminal taxa. For this reason, all new specimens of Triassic pterosaurs are important. As in the analysis by Britt et al. (2018), MCSNB 8950 (lacking skull, mandible and teeth) acts as a wildcard taxon. When MCSNB 8950 is pruned from the analysis, the nodal supports of the least inclusive nodes containing *Carniadactylus rosenfeldi* and the *Caviramus schesaplanensis* + *Raeticodactylus filisurensis* clade are 2 and 7, respectively.

Unlike other pterosaurs, *Seazzadactylus venieri* has hexa- and heptacuspid tooth crowns and mesialmost multicusped teeth in the upper and lower jaws that have a different shape with respect to the following multicusped teeth. This confirms that the pattern of the multicusped dentition is quite variable among Triassic pterosaur taxa.

The peculiar shape of the pterygoid-ectopterygoid, which more resembles that of the theropod dinosaur *Allosaurus* than that of other non-monofenestratan pterosaurs, is puzzling. This apparent divergence highlights the need to describe these elements in greater detail in other pterosaurs. The peculiar shape of the pteroid confirms the diagnostic importance of this bone, which was already evidenced by Dalla Vecchia (2009a). The jugal also appears to have diagnostic importance within non-monofenestratan pterosaurs.

With the addition of *Seazzadactylus venieri*, the pterosaur genera and species from the Dolomia di Forni Formation increase to four (the others are *Preondactylus buffarinii*, *Austriadactylus cristatus* and *Carniadactylus rosenfeldi*). The two basal clades of the Pterosauria are represented in this sample (Fig. 24). The pterosaur diversity of the Dolomia di Forni Formation is higher than those of the Early Jurassic formations that have yielded relatively abundant pterosaur remains (e.g. the Blue Lias of England and the Posidonienschiefer of Germany; Barrett et al., 2008). However, this higher systematic diversity may be only apparent. The geological time-spans represented by the fossil-bearing part of the Dolomia di Forni Formation and the Liassic formations are probably different. The Dolomia di Forni Formation is dated to the Alaunian 3-Sevatian (late middle-late Norian) based on conodont biostratigraphy (Dalla Vecchia, 2014). The Dolomia di Forni Formation probably represents only a part of the Sevatian, but the durations of the Alaunian and Sevatian have not been precisely established (Olsen, Kent & Whiteside, 2011) and could be several million years (the duration of the entire Norian is ca. 18.5 million years according to Cohen et al., 2013). The fossiliferous part of the Dolomia di Forni Formation is hundreds of metres thick and the different pterosaur taxa come from different levels within it (Dalla Vecchia, 2014). In contrast, the range of *Dorygnathus banthensis, Campylognathoides liasicus* and *Campylognathoides zitteli* in the Posidonienschiefer corresponds to the Lias ε II/1–6 (Padian, 2008a, 2008b), which extends from about the middle of the *Semicelatum* to the lower third of the *Elegans* Ammonoid Subzones (Riegraf, Werner & Lörcher, 1984) and could correspond to less than 700 ky (Ogg, Ogg & Gradstein, 2008). Whereas *Dorygnathus banthensis, Campylognathoides liasicus* and *Campylognathoides zitteli* were probably coeval, the taxa from the Dolomia di Forni Formation may not have been.

## Conclusions

MFSN 21545 represents a new taxon of non-monofenestratan pterosaur with multicusped dentition, *Seazzadactylus venieri*. This taxon is nested within a clade of Triassic pterosaurs that is basal within the Pterosauria but is not the basalmost clade. *Seazzadactylus venieri* is distinguished from other non-monofenestratan pterosaurs by the position of its teeth in the premaxilla; features of the jugal, pterygoid and ectopterygoid; the pattern of accessory cusps in the multicusped teeth; the shape of the crown of the first multicusped mandibular teeth; and the shapes of the scapular blade and pteroid. *Seazzadactylus venieri* is similar and closely related to *Carniadactylus rosenfeldi* and *Austriadraco dallavecchiai*, also found in the Alpine middle-upper Norian. It differs from *Carniadactylus rosenfeldi* (which is from the same formation and geographic location) in many further features, including: shape of the premaxilla; shape of the antorbital fenestra; jugal-maxilla articulation; shape of the jugal; absence of a jugal contribution to the antorbital fossa; shape of the first mandibular teeth; relative length of metacarpals I–III; relative length and shape of wing phalanx 1; and proportions of wing phalanges 1–3. It differs from *Austriadraco dallavecchiai* in additional features, including: shapes of the antorbital fenestra and jugal; absence of an external mandibular fenestra; shapes of the premaxillary and maxillary teeth; and shape of the coracoid.

The new taxon increases the diversity of Triassic pterosaurs and raises the number of pterosaur genera and species known from the Dolomia di Forni Formation to four.

## Supplemental Information

10.7717/peerj.7363/supp-1 Supplemental Information 1 Supplemental Information. Supplemental Text; Supplemental figures; Supplemental Tables; and Phylogenetic Analysis–Supplemental Information.

## Institutional Abbreviations

BNM: Bündner Naturmuseum, Chur, Switzerland
BSP: Bayerische Staatssammlung für Paläontologie und Geologie, Munich, Germany
MCSNB: Museo Civico di Scienze Naturali, Bergamo, Italy
MFSN: Museo Friulano di Storia Naturale, Udine, Italy
MGC: Museo Geologico della Carnia, Ampezzo, Italy
MGUH: Geological Museum, University of Copenhagen, Denmark
MPUM: Museo di Paleontologia dell’Università di Milano, Milano, Italy
PIMUZ: Paläontologisches Institut und Museum der Universität, Zürich, Switzerland
SMNS: Staatliches Museum für Naturkunde, Stuttgart, Germany.

## Additional Information and Declarations

### New Species Registration

The following information was supplied regarding the registration of a newly described species: Publication LSID: urn:lsid:zoobank.org:pub:5F0C4B84-F39D-436F-93FD-858B323C6A15. *Seazzadactylus* LSID: urn:lsid:zoobank.org:act:1B567D5D-E9BC-41A0-BA73-04A29F496989. *Seazzadactylus venieri* LSID: urn:lsid:zoobank.org:act:02CB1E39-1338-49F7-8493-37C474ED7663.

### Data Availability

The following information was supplied regarding data availability: Tables of measurements and ratios are available as Supplemental Files.
