# Supplementary material for: Seazzadactylus venieri gen. et sp. nov., a new pterosaur (Diapsida: Pterosauria) from the Upper Triassic (Norian) of northeastern Italy
Source: PeerJ. 2019 Jul 25;7:e7363. doi: 10.7717/peerj.7363 (PMC6661147; doi:10.7717/peerj.7363)
Supplement: Supplemental Information 1 — Supplemental Text; Supplemental figures; Supplemental Tables; and Phylogenetic Analysis–Supplemental Information. [file peerj-07-7363-s001.pdf]

Seazzadactylus venieri gen. et sp. nov., a new pterosaur (Diapsida: Pterosauria) from the Upper Triassic (Norian) of northeastern Italy

Fabio M. Dalla Vecchia

Institut Català de Paleontologia Miquel Crusafont (ICP), Carrer de l'Escola Industrial 23, E-08201, Sabadell, Spain; Museo Friulano di Storia Naturale, Via Sabbadini 22-32, I-33100 Udine, Italy.

E-mail: fabio.dallavecchia@icp.cat

Supplemental Information

Supplemental Information 1

The skeletal remains of MFSN 21545 are preserved on a dark gray dolostone slab that was broken into several pieces by unknown fossil collectors who did not realize that they had found a pterosaur fossil. The broken slab was abandoned on the Seazza Brook bed where the boulder containing it cropped out within the debris; but it was later collected by Umberto Venier in 1997 and brought to the MFSN. Its surviving pieces were subsequently reassembled, but many small parts were found to be missing (SI Fig.1). Fragments of the slab and counterslab were glued together and the specimen prepared for exhibit purposes by Urs Oberli in 2001. The missing parts (rock and bone) were reconstructed with artificial resin and coloured like the original part (artificial resin parts are distinguished from rock and fossil bone in text-figures and in Fig.S1).

Supplemental Information 2

Morphology of the maxillary teeth

Tooth1, left - Missing.

Tooth1, right - The crown (Fig. 12A) is apicobasally higher than mesiodistally long, with an inflated basal part and a distally recurved apical part. It is procumbent and smaller than the crowns of the following teeth. A very small accessory cusp might be present distally, but the crown appears to be basically unicuspid.

Tooth 2, right - The crown (Fig. 12B) has a single small basal accessory cusp mesially and two larger accessory cusp distally, and is thus tetracuspid. The crown is slightly procumbent, slightly recurved distally, and is apicobasally higher than mesiodistally long (with proportions that are similar to those of mandibular tooth 3).

Tooth 2, left - Its crown (Fig. 12C) has the same shape as the right crown but has three accessory cusps distally, and is thus pentacuspid. Distal accessory cusps increase in size from the basal to the apical one; the single mesial accessory cusp is at the level of the apical accessory cusp on the other margin.

Tooth 3, right - The crown (Fig. 12D) is pentacuspid with two accessory cusps distally and two mesially. The crown is slightly recurved distally and slightly procumbent.

Tooth 3, left - The crown has two mesial and three distal accessory cusps, and is thus hexacuspid. The apical cusp is larger than the others and the basal distal one is very small. The crown is slightly recurved distally and slightly procumbent. The apex of the main cusp is worn or damaged.

Tooth 4, right - The crown (Fig. 12E) is neither recurved nor procumbent and has a cuspidation pattern as the crown of tooth 3, but with a further very small, accessory cusp at the base of the distal side, and is therefore hexacuspid.

Tooth 4, left - The crown is broken apically. Two mesial and one distal accessory cusps are preserved. The only distal accessory cusp is as large as the apical accessory cusp of the right crown 4, and the left tooth 4 was thus probably tetracuspid.

Teeth 5 - Right (Fig. 12E) and left crowns are both pentacuspid, with two accessory cusps mesially and distally. Mesial and distal accessory cusps are similar in shape and size and are pointed. The apical accessory cusp is the larger of the two. Crowns are upright and have symmetrical main cusps like those of the following teeth. They are slightly apicobasally higher than mesiodistally long.

Tooth 6, right - The crown is probably pentacuspid, but the apical mesial and basal distal accessory cusps are damaged.

Tooth 6, left - This tooth is not fully erupted and shows only the apices of a mesial and a distal accessory cusp.

Teeth 7 - Both right and left crowns are pentacuspid and similar to the crowns of teeth 5. The apex of the main cusp of the left crown is damaged.

Tooth 8, right - The crown is not fully erupted and shows only the apical accessory cusps.

Tooth 8, left - The position of this tooth is represented by an empty alveolus, but the displaced tooth is preserved close by (Fig. 12F). The crown is like that of tooth 7, but bears a further, very small and acute basal accessory cusp along each cutting margin. Therefore, this tooth is heptacuspid. The 'root' is tongue-shaped and as deep as the crown is high.

Tooth 9, right - The crown (Fig. 12G) has two accessory cusps mesially and three distally, and is therefore hexacuspid. The apical accessory cusp is much larger and blunter than the others, whereas the basal distal cusp is very small.

Tooth 9, left - The crown is pentacuspid with two mesial and two distal accessory cusps and is slightly mesiodistally longer than apicobasally high.

Tooth 10, right - The crown is not fully erupted and shows two mesial and one distal accessory cusps, but the apex of a further basal cusp is possibly present distally. Therefore, the crown appears to be tetracuspid, but is probably pentacuspid.

Tooth 10, left - The crown has two distal and one mesial accessory cusps, but a mesial basal cusp was probably broken. Therefore, the crown appears to be tetracuspid, but it is probably pentacuspid.

Tooth 11, right - The crown (Fig. 12H) has a basal, small mesial accessory cusp and two distal accessory cusps (the apical accessory cusp is the largest) and thus it is tetracuspid. The mesial cutting margin of the main cusp is slightly concave instead of straight as in the other crowns and the main cusp appears to be more slender than the others.

Tooth 11, left - The crown is pentacuspid, with two mesial and two distal accessory cusps and is slightly mesiodistally longer than apicobasally high.

Tooth 12, right - The crown (Fig. 12I) is pentacuspid with two distal and two mesial accessory cusps and is the first crown to be much lower apicobasally than wide mesiodistally.

Tooth 12, left - The crown is pentacuspid and like the left crown 11, but it is smaller.

Tooth 13, right - the crown (Fig. 12I) is pentacuspid and similar to the crown of right tooth 12.

Tooth 13, left - A gap of about 2.2 mm in length between crown 12 and the following crown suggests the presence of an empty alveolus, and maybe even two. I consider as more probable the presence of a single alveolus because the right maxilla has 14 teeth and the distal teeth are more widely spaced than the mid-mesial teeth.

Tooth 14, right - The crown (Fig. 12I) is the smallest of the whole right maxillary dentition. This tooth is probably not fully grown because its crown is much smaller than the preceding crown. The main cusp is damaged. There are only two relatively large accessory cusps, one along each cutting margin.

Tooth 14, left - Only the apical part of the main cusp of the erupting crown is visible.

Morphology of the multicuspid teeth of the right mandibular ramus

Tooth 3 - The crown (Fig. 15C) is heptacuspid with three small accessory cusps per margin. It is upright and much apicobasally higher than mesiodistally long.

Tooth 4 - The crown is tetracuspid with two accessory cusps at the base of the mesial margin and one at the base of the distal margin (Fig. 15D). It is upright and much apicobasally higher than mesiodistally long, with small accessory cusps.

Tooth 5 - The crown is not fully erupted and only the main cusp and the most apical cusp along the mesial margin are visible.

Tooth 6 - The crown is pentacuspid, with two accessory cusps at each side (Fig. 15E). It is apicobasally higher than mesiodistally long, but is comparatively longer mesiodistally than the preceding crowns and has main cusps that are more flattened labiolingually; the two accessory cusps at the base of each cutting margin are larger than those of crowns 3-4 and the apical accessory cusps are larger than the basal cusps.

Tooth 7 - The crown is pentacuspid, with two accessory cusps on each side. The morphology of the crown and cusps is the same as that of tooth 6.

Tooth 8 - The crown is pentacuspid, with two accessory cusps on each side.

Tooth 9 - The crown is pentacuspid, with two accessory cusps on each side (Fig. 15F). It is like the crown of tooth 7, but it is slightly larger, stouter and with comparatively larger accessory cusps.

Tooth 10 - The crown is pentacuspid, with two accessory cusps on each side. It has a basal labial depression, possibly because of the reabsorption by the replacement crown growing inside the basal part of the functional crown or just because of the collapse of the pulp cavity.

Tooth 11 - The crown is pentacuspid, with two accessory cusps at each side (Fig. 15G). The apical part of the replacement tooth growing inside the pulp cavity of the functional tooth is visible at the base of the crown, which is labially reabsorbed.

Tooth 12 - The crown is pentacuspid, with two accessory cusps at each side. It is nearly as mesiodistally wide as apicobasally tall and is the largest multicusped crown in the mandible (height ~1.5 mm) together with the crown of tooth 14. It has a basal depression like tooth 10.

Tooth 13 - The crown is hexacuspid with two accessory cusps mesially and three distally (Fig. 15H). It has a basal depression like tooth 10.

Tooth 14 - The crown is pentacuspid, with two accessory cusps at each side. It is nearly as mesiodistally wide as it is apicobasally tall. It has a basal depression like tooth 10.

Tooth 15 - The crown is pentacuspid, with two accessory cusps at each side. It has a basal depression like tooth 10.

Tooth 16 - The crown is tetracuspid with one accessory cusp at the base of the mesial margin and two at the base of the distal margin. However, the basal accessory cusp of tooth 15 overlaps the margin of the crown basomesially. Thus a further small accessory cusp may be present and concealed. The apical part of the replacement tooth growing inside the pulp cavity of the functional tooth is visible at the base of the crown, which is labially reabsorbed.

Tooth 17 - The crown is pentacuspid with two accessory cusps at each side. It is the first crown to be mesiodistally longer than apicobasally high. Its main cusp is slightly asymmetrical distally. It has a basal depression like tooth 10.

Tooth 18 - The crown is not fully erupted, showing the main cusp and the apices of the upper mesial and distal cusps.

Tooth 19 - The crown is tetracuspid with two accessory cusps at the base of the mesial margin and one at the base of the distal margin. Its shape is like that of the crown of tooth 17.

Tooth 20 - The crown is not fully erupted and shows only the apical part of the main cusp and the point of the apical accessory cusps

Tooth 21 - The crown is not fully erupted and shows only the apical part of the main cusp and the point of the apical distal accessory cusp.

Supplemental figures

Figure S1: ***Seazzadactylus venieri*, MFSN 21545 (holotype), the specimen before preparation.** (A) The reassembled fragments of the slab before preparation; (B) drawing of the prepared specimen in which the bones that can be recognized in (A) and the principal parts of the skeleton are labeled. The rock is pale gray in colour, the parts reconstructed in resin are dark gray and the bones are white. Abbreviations: fe, femur; h, humerus; ra, radius; u, ulna; wph1, 3 and 4, wing phalanges 1, 3 and 4. Elements in parentheses are from the left side. In (A) the scale bar is in centimetres; in (B), the scale bar equals 2 cm.

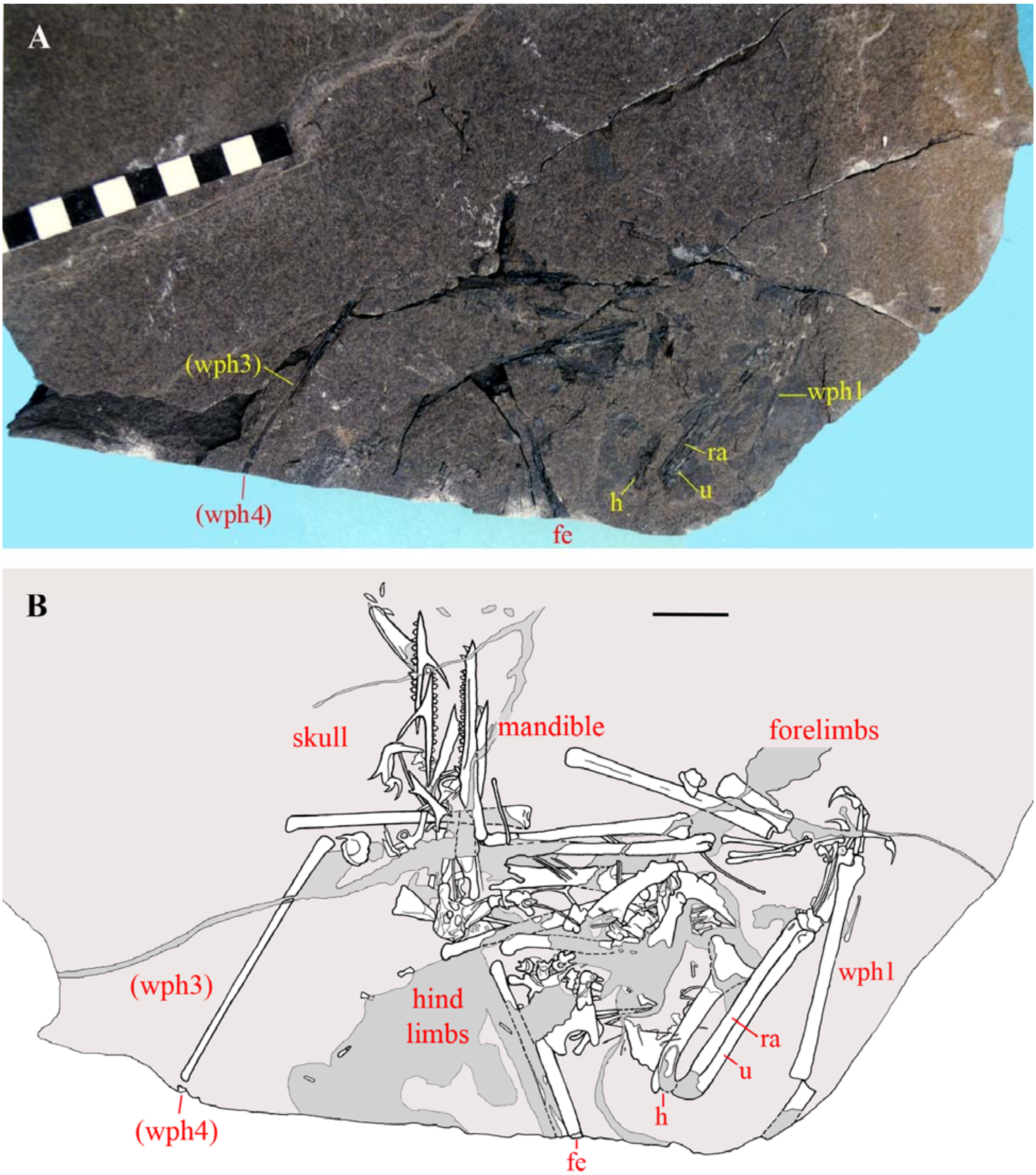

Figure S2: **Location of *Seazzadactylus venieri*, MFSN 21545 (holotype).** The asterisk marks the location of the specimen within the Friuli Venezia Giulia Autonomous Region of NE Italy.

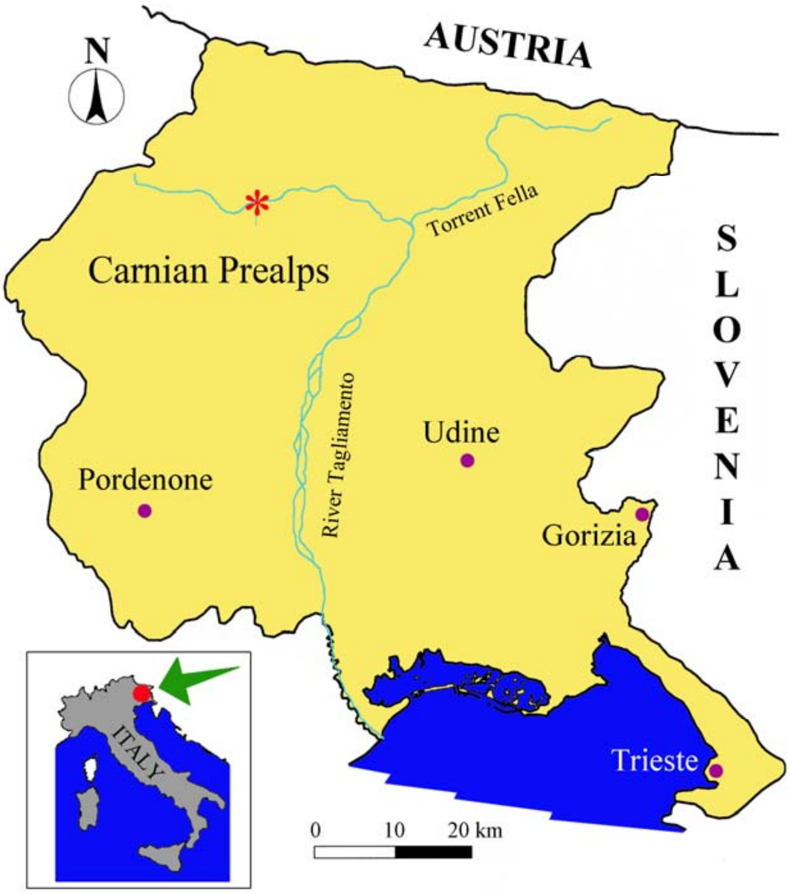

Figure S3: ***Seazzadactylus venieri*, MFSN 21545 (holotype), maxillo-jugal articulation.** (A) Reconstruction with the jugal overlapping the jugal process of maxilla dorsally (as in *E. ranzii*); (B) reconstruction with the jugal overlapping the jugal process of maxilla laterally (as in *Di. macronyx*) to expose the last two maxillary teeth, but producing a step along the ventral margin of the antorbital fenestra. The right jugal and maxilla are used in the reconstruction. The incompletely exposed rostral termination of the premaxillary process of the right maxilla was integrated with the rostral termination of the premaxillary process of the left maxilla (colour of the part from the left maxilla is darker to show this integration). Abbreviations: aof, antorbital fenestra; en, external naris; j, jugal; mx, maxilla; or, orbit.

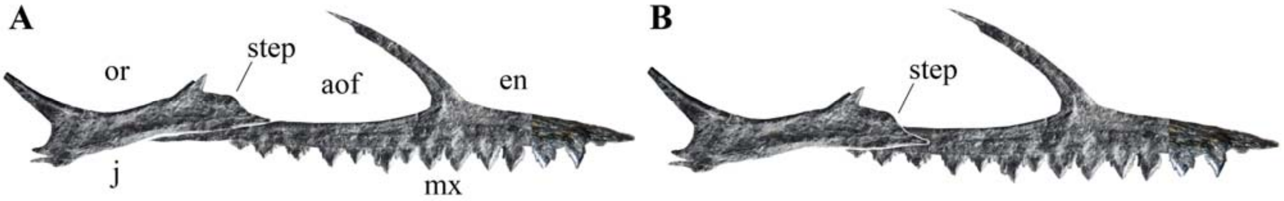

Figure S4: ***Seazzadactylus venieri*, MFSN 21545 (holotype), comparison between the maxillo-jugal bar bordering the antorbital fenestra ventrally in '*Raeticodactylus*' *filisurensis* and the reconstruction of the bar in MFSN 21545**. (A) the maxillo-jugal bar in the holotype of '*Raeticodactylus*' *filisurensis* (mirrored); (B) reconstruction of the maxillo-jugal bar of MFSN 21545 in which the maxillary process of the jugal and the jugal process of maxilla articulate to obtain a linear ventral margin of the antorbital fenestra like that seen in the holotype of '*Raeticodactylus*' *filisurensis*. Abbreviations: aof, antorbital fenestra; ap, ascending process of maxilla; en, external naris; j, jugal; jp, jugal process of maxilla; la, lacrimal; mx, maxilla; mxp, maxillary process of jugal; or, orbit. Not to scale.

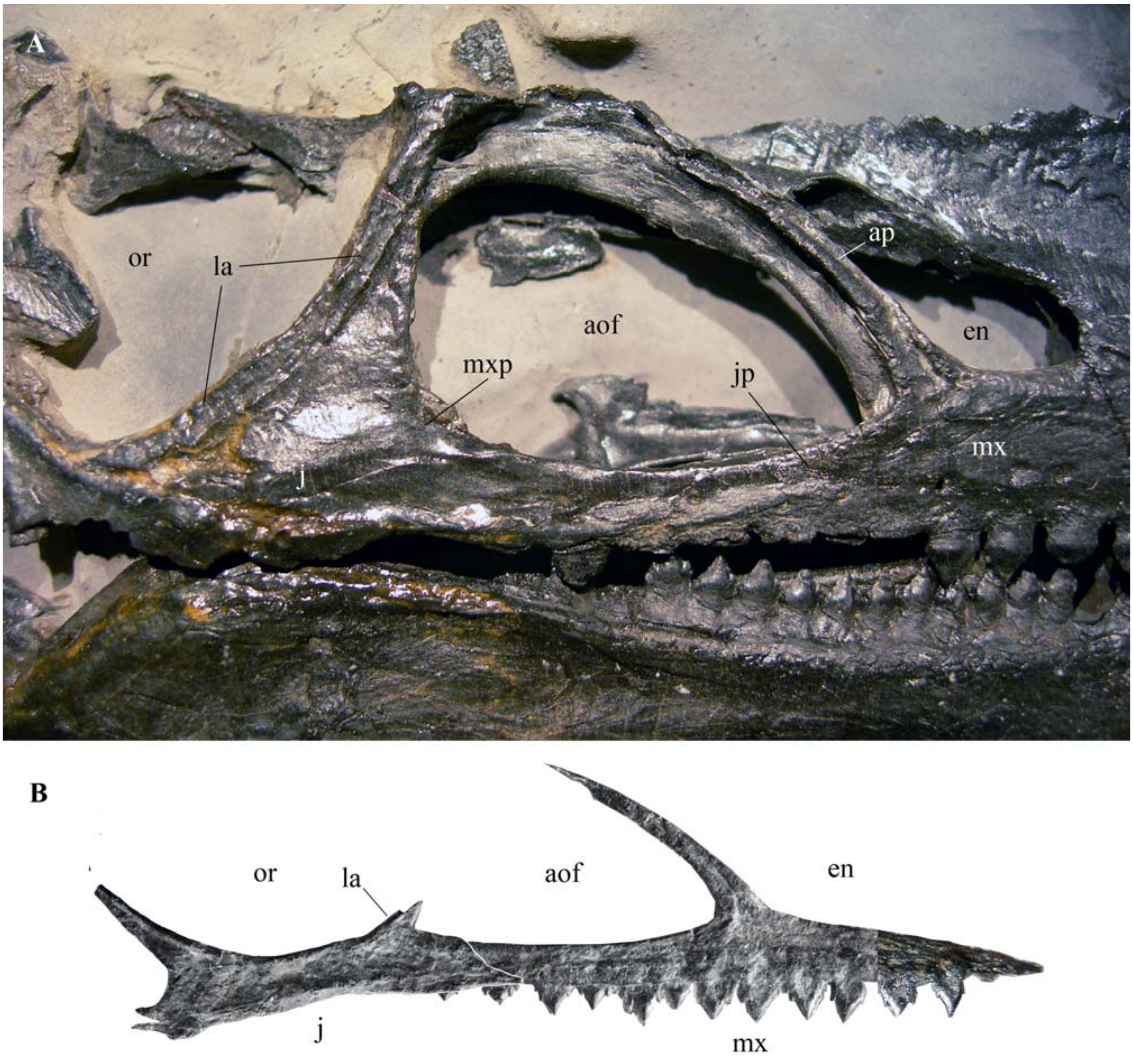

Figure S5: ***Seazzadactylus venieri*, MFSN 21545 (holotype), comparison of the sizes of the maxillary and mandibular teeth**. (A) Right maxilla; (B) right mandibular ramus. Numbers refer to the tooth positions. Scale bar equals 5 mm.

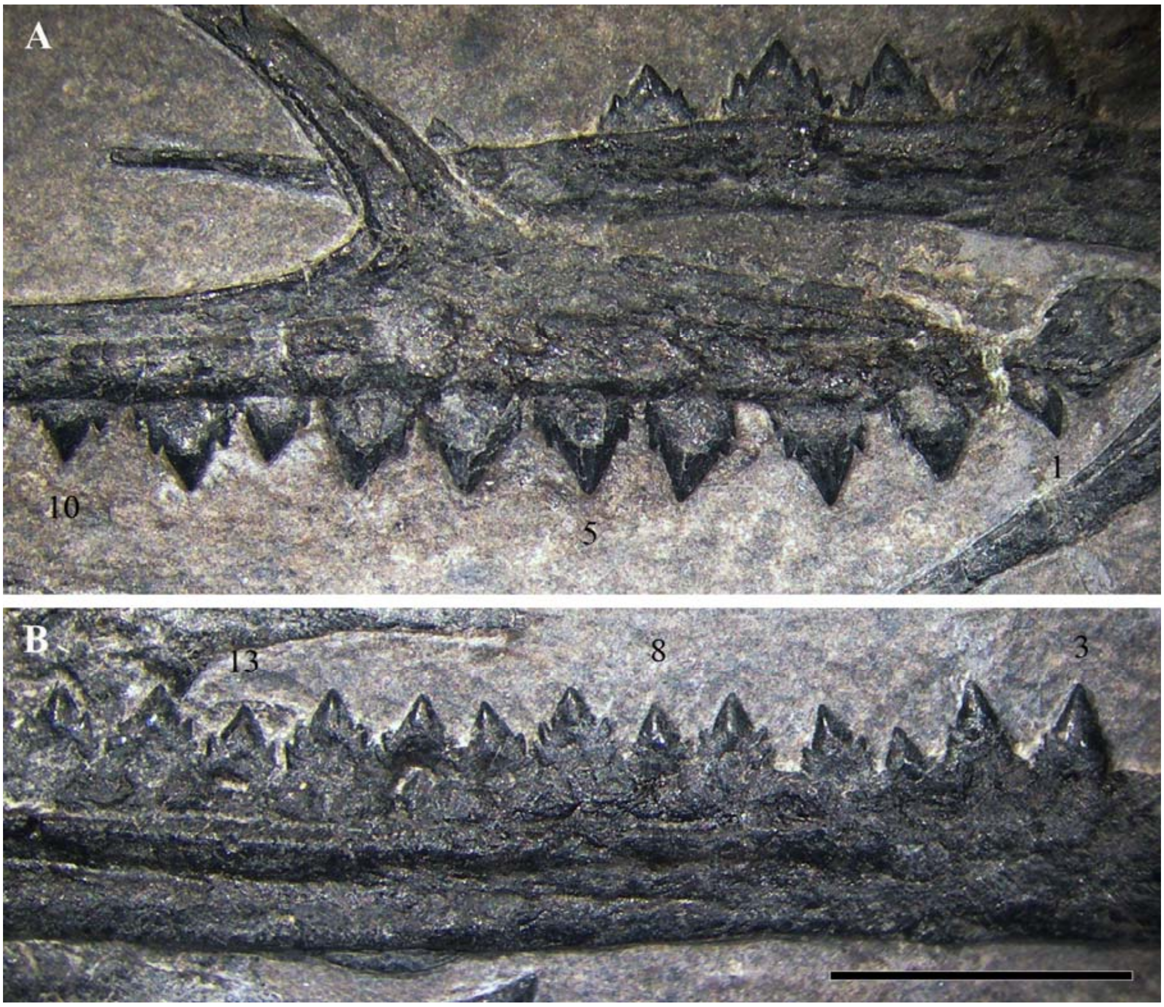

Figure S6: ***Seazzadactylus venieri*, MFSN 21545 (holotype), left radius and ulna.** Distal part of the two bones in cranial view. Abbreviations: dtr, distal tubercle; fu, furrow; ra, radius; u, ulna. Scale bar equals 1 mm.

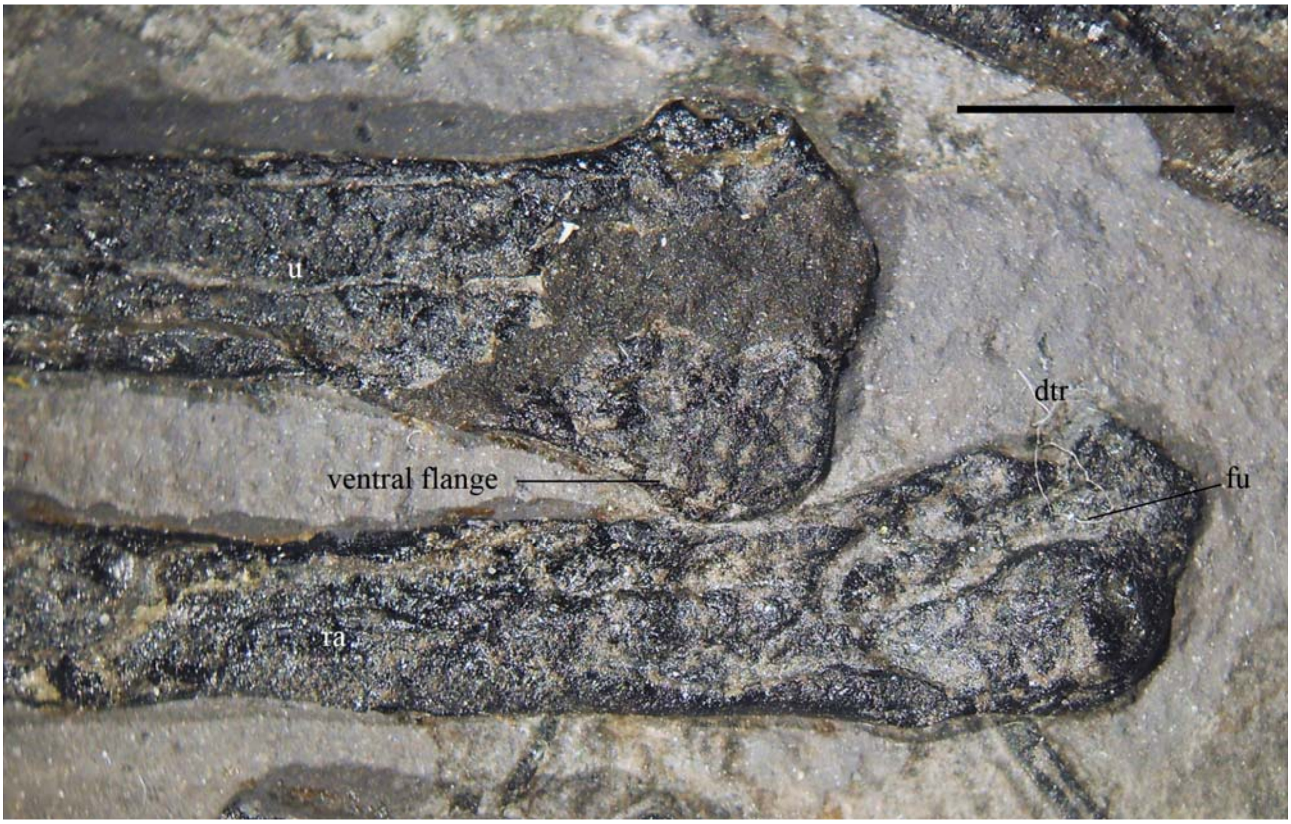

Figure S7: ***Seazzadactylus venieri*, MFSN 21545 (holotype), hind limb elements.** (A) Proximal part of the left femur in cranial view; (B) distal part of the right tibiotarsus in caudal view; (C) distal part of the left tibiotarsus in medial view. Abbreviations: cfe, proximal head of the femur (caput femoris); lco, lateral condyle; mec, medial epicondyle; mco, medial condyle. Scale bar equals 5 mm.

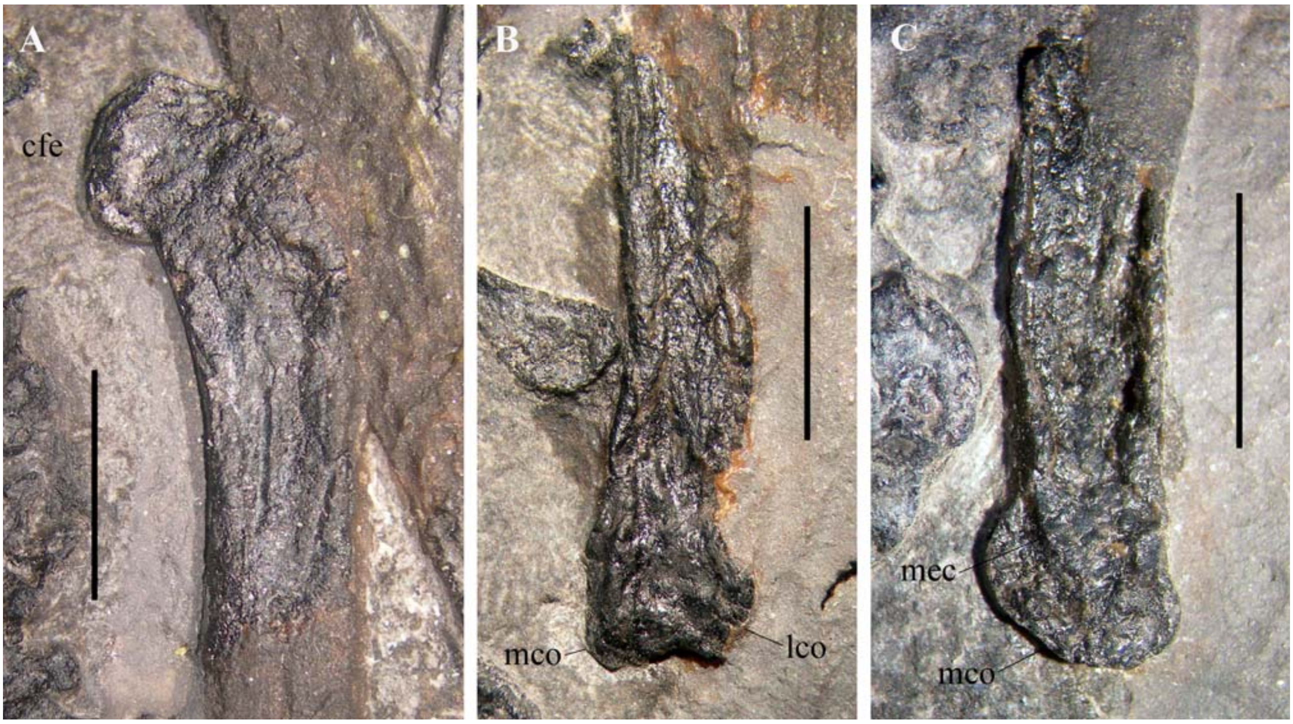

Figure S8: ***Seazzadactylus venieri*, MFSN 21545 (holotype), size comparison with the holotype of *Eudimorphodon ranzii* (MCSN 2888).** (A) Drawing of MFSN 21545; (B) drawing of MCSNB 2888 (based on Wild, 1979, modified). Scale bar equals 20 mm.

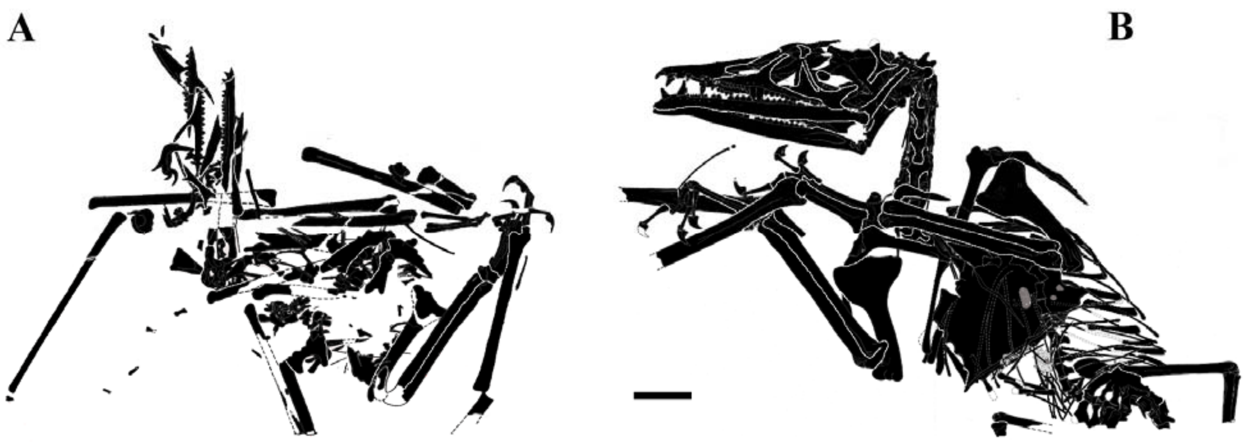

Supplemental tables

Table S1: **Long bone lengths of *Seazzadactylus venieri*, MFSN 21545 (holotype), and other Triassic pterosaurs.** 1, MFSN 21545 (bold); 2, MCSNB 2888, holotype of *Eudimorphodon ranzii*; 3, MFSN 1797, holotype of *Carniadactylus rosenfeldi*; 4, MPUM 6009, referred specimen of *Carniadactylus rosenfeldi*; 5, MGUH VP 3393, holotype of *Arcticodactylus cromptonellus*; 6, BNM 14524, holotype of '*Raeticodactylus' filisurensis*; 7, BSP 1994 I 51, holotype of *Austriadraco dallavecchiai*; 8, MCSNB 8950, still unnamed taxon; 9, MCSNB 2887, indeterminate pterosaur; 10, MCSNB 2886, holotype of *Peteinosaurus zambellii*; 11, MCSNB 3359, referred specimen of *Peteinosaurus zambellii*; 12, SMNS 56342, holotype of *Austriadactylus cristatus*; 13, MGC 332466, referred specimen of *Austriadactylus cristatus*; and 14, MFSN 1770, holotype of *Preondactylus buffarinii*. Data are mostly from Dalla Vecchia (2014). Abbreviations: co, coracoid; fe, femur; h, humerus; lj, mandibular ramus; mcI-IV, metacarpals I-IV; pt, pteroid; ra, radius; sk, skull; ti, tibia/tibiotarsus; u, ulna; wph1-4, wing phalanges 1-4. Symbols: *, measurements estimated or approximate, +, dubious identification.

	**1**	**2**	**3**	**4**	**5**	**6**	**7**	**8**	**9**	**10**	**11**	**12**	**13**	**14**
sk	**65***	90*	55*	41.5	-	95	-	-	-	-	-	110	72	56*
lj	**53.5**	74.5	-	34	-	84	50*	-	-	60*	-	-	-	54
co	**≥21**	32.6	22.5	14.5*	>6.5	-	18	10.8	15	-	23	-	-	
h	**44**	47	42*	26.3	18.15	82	40	26	28	-	39	70-75*	36	32
u	**53-55***	65	55	36	20.1	106	-	33.5	38	-	49.5	96.5	50	42
mcIV	**20.7**	29	21	10.5	8.4	-	-	9	-	16.5	17	33*	15.7	14.25
mcIII	**18**	-	19.2	~10	8.3	42+	-	-	-	-	15*	-	14.4	14
mcII	**18**	-	17.2	9.5-10	7.4	41+	-	<9	-	-	14.7*	-	14.2	14
mcI	**14**	-	13.7	~6.5	5.6	40+	-	<<9	-	-	14.3*	-	13*	9.5
pt	**12.4**	19.5	16	>5.7	-	-	-	-	~14	-	9.7	-	-	7.25
wph1	**60.5**	80*	64	37.5	18*	113	52.9	34	40	42.5	42.5	95*	40	35.5
wph2	**63.5**	-	58.2	33*	20.5	109	-	35.3	36.5	41*	42.5	101	-	39
wph3	**72**	-	63.2	36.2	20.5*	117*	-	36.2	-	-	46.5	103.5	-	39
fe	**>32**	41	37*	18.5*	19.7	56	38*	19.6	21.2	-	37	-	-	32.5
ti	**55***	50*	54.2	-	20.5*	84	57.7	25	28.5	51.5	48	-	-	44

Table S2: **Lengths of the phalanges of manual digits I-III of *Seazzadactylus venieri*, MFSN 21545 (holotype).** Measurements in parentheses refer to elements from the left side. Abbreviations: phI-III, manual digit I-III; uphI-III, ungual phalanx I-III. Symbols: *, measurements estimated or approximate; #, incomplete element.

phI-1	uphI	phII-1	phII-2	uphII	phIII-1	phIII-2	phIII-3	uphIII
8* (8.7)	6.7(6.2)	5#(5.3)	7#(8.7)	6.5#(7)	7(?)	4.7(?)	9(8.5#)	6.8(6.8)

Table S3: **Percent length of the long bones of some Triassic pterosaur specimens with respect to the same element in MFSN 21545, holotype of *Seazzadactylus venieri*.** The specimens are listed in order of decreasing sizes. Specimens: BNM 14524, holotype of '*Raeticodactylus' filisurensis*; BSP 1994 I 51, holotype of *Austriadraco dallavecchiai*; MCSNB 2887, indeterminate pterosaur; MCSNB 2888, holotype of *Eudimorphodon ranzii*; MCSNB 8950, still unnamed taxon; MFSN 1797, holotype of *Carniadactylus rosenfeldi*; MGUH VP 3393, holotype of *Arcticodactylus cromptonellus*; MPUM 6009, referred specimen of *Carniadactylus rosenfeldi.* Symbols and anatomical abbreviations as in Table S1, plus M, mean.

**specimen**	**lj**	**h**	**u**	**mcIV**	**wph1**	**wph2**	**wph3**	**ti**	**M**
BNM 14524	157	186	196	-	187	172	162*	144*	172
MCSNB 2888	139	107	120	140	-	-	-	85*	118
MFSN 1797	-	95	102	101	106	92	88	93*	97
BSP 1994 I 51	-	91	-	-	87	-	-	99	92
MCSNB 2887	-	64	70	-	66	57	-	49	61
MPUM 6009	64	60	67	51	62	52*	50	-	58
MPUM 8950	-	59	62	43	56	56	50	43	53
MGUH VP 3393	-	41	37	41	30*	32	28*	35*	35

Table S4: **Ratios of long bone lengths of Triassic pterosaurs.** 1, MFSN 21545, holotype of *Seazzadactylus venieri* (bold); 2, MCSNB 2888, holotype of *Eudimorphodon ranzii*; 3, MFSN 1797, holotype of *Carniadactylus rosenfeldi*; 4, MPUM 6009, referred specimen of *Carniadactylus rosenfeldi*; 5, MGUH VP 3393, holotype of *Arcticodactylus cromptonellus*; 6, BNM 14524, holotype of '*Raeticodactylus*' *filisurensis*; 7, BSP 1994 I 51, holotype of *Austriadraco dallavecchiai*; 8, MCSNB 8950, still unnamed taxon; 9, MCSNB 2887, indeterminate pterosaur; 10, MCSNB 2886, holotype of *Peteinosaurus zambellii*; 11, MCSNB 3359, referred specimen of *Peteinosaurus zambellii*; 12, SMNS 56342, holotype of *Austriadactylus cristatus;* 13, MGC 332466, referred specimen of *Austriadactylus cristatus*; 14, MFSN 1770, holotype of *Preondactylus buffarinii*. Symbols and abbreviations as in Table S1.

	**1**	**2**	**3**	**4**	**5**	**6**	**7**	**8**	**9**	**10**	**11**	**12**	**13**	**14**
u/h	**1.20-1.25**	1.38	1.31	1.37	1.11	1.29	-	1.29	1.36	-	1.30	1.29-139*	1.39	1.31
h/mcIV	**2.13**	1.62	1.93	2.50	2.16	-	-	2.89	-	-	2.29	-	2.29	2.25
u/mcIV	**2.56-2.66**	2.24	2.62	3.43	2.39	-	-	3.72	-	-	2.91	2.92*	3.18	2.95
h/fe	**<1.47**	1.14	1.13	1.42	0.92	1.46	1.05*	1.33	1.32	-	1.05	-	-	0.98
h/ti	**0.80***	0.94*	0.77	-	0.88*	0.98	0.69	1.04	0.98	-	0.81	-	-	0.73
u/fe	**<1.8**	1.58	1.49	1.95	1.02	1.89	-	1.71	1.79	-	1.34	-	-	1.29
u/ti	**0.96-1.00***	1.30	1.02	-	0.98*	1.26	-	1.34	1.33	-	1.03	-	-	0.95
u/pt	**4.27-4.43**	3.33	3.44	<6.3	-	-	-	-	2.71-	-	5.10	-	-	5.79
ti/fe	**<1.72***	1.22*	1.46	-	1.04*	1.50	1.52*	1.27	1.34	-	1.30	-	-	1.35
fe/mcIV	**>1.45**	1.41	1.76	1.76	2.34	-	-	2.18	-	-	2.18	-	-	2.28
ti/mcIV	**2.66***	1.72*	2.58	-	2.44*	-	-	2.78	-	3.12	2.82	-	-	3.09
wph1/h	**1.38**	1.70*	1.52	1.43	0.95*	1.38	1.32	1.31	1.43*	-	1.09	1.27-1.36*	1.11	1.11
wph1/u	**0.96-1.10**	1.23*	1.16	1.04	0.90*	1.07	-	1.01	1.05*	-	0.86	0.98*	0.80	0.85
wph1/mcIV	**2.92**	2.75*	3.05	3.57	2.24*	-	-	3.78	-	2.57	2.50	2.88*	2.55	2.49
wph1/fe	**<2.02**	1.95*	1.73	2.03	0.91*	2.02	1.39	1.73	1.89*	-	1.15	-	-	1.09
wph1/ti	**1.10***	1.60*	1.18	-	0.89*	1.34	0.92	1.36	1.40*	0.82	0.88	-	-	0.81
wph2/wph1	**1.05**	-	0.91	0.88	1.14*	0.96	-	1.04	0.91*	0.96*	1.00	1.06*	-	1.10
wph3/wph2	**1.13**	-	1.09	1.10*	1.00*	1.07*	-	1.02	-	-	1.09	1.02	-	1.00
wph3/wph1	**1.19**	-	0.99	0.96*	1.14*	1.03*	-	1.06	1.03*	-	1.09	1.09	-	1.08

Phylogenetic analysis. Supplemental information

Scorings for *Seazzadactylus venieri*

001??03011   01?--?0?00   010--00020   1000-10011   01100-0000   0000?0????   1?1??11?01
11011??001   1000?010/1??   ???
